# Correlation of mRNA and protein levels: Cell type-specific gene expression of cluster designation antigens in the prostate

**DOI:** 10.1186/1471-2164-9-246

**Published:** 2008-05-23

**Authors:** Laura E Pascal, Lawrence D True, David S Campbell, Eric W Deutsch, Michael Risk, Ilsa M Coleman, Lillian J Eichner, Peter S Nelson, Alvin Y Liu

**Affiliations:** 1 Department of Urology, University of Washington, Seattle WA 98195, USA; 2 Institute for Stem Cell and Regenerative Medicine, University of Washington, Seattle WA 98195, USA; 3 Institute for Systems Biology, Seattle WA 98103, USA; 4 Department of Pathology, University of Washington, Seattle WA 98195, USA; 5 Fred Hutchinson Cancer Research Center, Seattle WA 98109, USA

## Abstract

**Background:**

Expression levels of mRNA and protein by cell types exhibit a range of correlations for different genes. In this study, we compared levels of mRNA abundance for several cluster designation (CD) genes determined by gene arrays using magnetic sorted and laser-capture microdissected human prostate cells with levels of expression of the respective CD proteins determined by immunohistochemical staining in the major cell types of the prostate – basal epithelial, luminal epithelial, stromal fibromuscular, and endothelial – and for prostate precursor/stem cells and prostate carcinoma cells. Immunohistochemical stains of prostate tissues from more than 50 patients were scored for informative CD antigen expression and compared with cell-type specific transcriptomes.

**Results:**

Concordance between gene and protein expression findings based on 'present' *vs*. 'absent' calls ranged from 46 to 68%. Correlation of expression levels was poor to moderate (Pearson correlations ranged from 0 to 0.63). Divergence between the two data types was most frequently seen for genes whose array signals exceeded background (> 50) but lacked immunoreactivity by immunostaining. This could be due to multiple factors, e.g. low levels of protein expression, technological sensitivities, sample processing, probe set definition or anatomical origin of tissue and actual biological differences between transcript and protein abundance.

**Conclusion:**

Agreement between these two very different methodologies has great implications for their respective use in both molecular studies and clinical trials employing molecular biomarkers.

## Background

Immunostaining and microarray analysis are techniques frequently used to characterize tissue phenotypes. Immunohistochemistry (IHC) is a method of assessing protein levels of gene expression that is based on the ability of antibodies to bind proteins expressed by cells in sections of frozen or formalin-fixed, paraffin-embedded tissues. IHC enables one to detect and localize a specific antigen to specific cell types. Gene arrays determine expression levels for thousands of genes simultaneously by detecting sequence segments or partial segments of mRNA in a sample. To fully understand the underlying mechanisms of biological processes, it is essential to determine whether observed changes in mRNA can also be seen in the translated protein, and to pinpoint what cell types are exhibiting these changes. Gene array analysis and immunostaining are powerful tools for determining gene and protein expression patterns in health and diseases. Establishing the extent of agreement between semi-quantitative immunostaining data and gene array data obtained from sorted cell populations and tissue specimens is important to account for possible discrepancies between these two very different methods.

Determining a direct relationship between protein and mRNA levels can be problematic, and previous efforts to find correlations have found variable success. A study comparing yeast proteomic and transcriptomic data showed that correlation was insufficient to predict protein expression levels from mRNA except for the most abundant proteins, suggesting that protein abundance may be a factor that influences the correlation between mRNA and protein [1]. However, a relationship between mRNA/protein correlation coefficient and protein abundance was not observed in a study of human lung adenocarcinomas [2] or in a study of MMP-2, MMP-9 and TIMP-1 in human prostate cancers [3]. For some genes, such as HER2/neu, expression levels assayed by RT-PCR, IHC and fluorescence in situ hybridization (FISH) in breast tumors show highly significant correlation among these techniques [4]. However, studies evaluating the overall concordance between protein and RNA expression levels have found wide variability. For example, transcript and protein concordance in the LNCaP prostate cancer cell line has been reported to vary from 32% [5] to 83.5% [6]. Highly significant correlations in mRNA changes and protein expression levels were found by Orntoft *et al*. in human carcinomas [7]. Studies such as these suggest that external factors as well as actual biological differences between mRNA and protein abundance might affect the relationships between the two data types.

The assessment of CD24 as a potential prostate cancer biomarker through RNA expression profiling and IHC analysis in previous studies further illustrates the difficulties in directly comparing gene and protein expression levels. Normalized CD24 transcript levels showed an average 2.69-fold increase in prostate cancer as determined by qPCR [8] and an increase in staining intensity as determined by IHC [9]. Two reports by Kristiansen et al. found differential CD24 gene expression in 38.5% of tumor cases as determined by Affymetrix GeneChip analysis [10] and 48% as examined by IHC [11]. It is unclear if the 10% disparity between these studies has biological significance, or if a component of the measured difference is due to technical attributes of the assays. Although, based on these studies, CD24 could potentially be an important prognostic prostate cancer tissue marker, the relationship between mRNA levels and resulting protein expression remains unclear.

Our laboratory has previously characterized benign and neoplastic prostate tissues by CD phenotype [9,12] and isolated the constituent cell types with magnetic cell sorting (MACS) for gene expression analysis [13]. In the present study, we compared the expression levels of a panel of 58 informative CD antigens in prostate tissue scored by immunostaining with expression levels of the respective mRNA by microarray-based quantitation of MACS-sorted and laser capture microdissected (LCM) cell populations. Our objective was to characterize the relationship between protein and mRNA levels measured by these two techniques.

## Results

### Immunolocalization and transcriptome data summary

**Figure 1:**
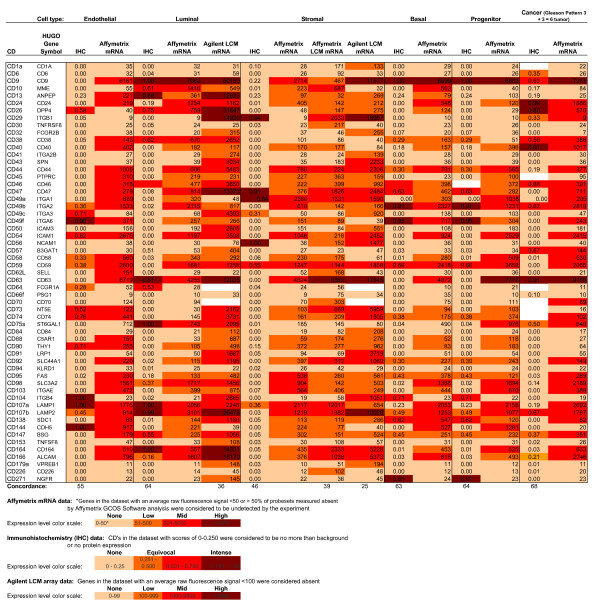
**CD expression summary**. CD expression levels of prostate tissue as scored by immunostaining (IHC) and gene array (mRNA) for each constituent cell type. Overall concordance between IHC and MACS-sorted cell gene array ranged from 46 to 67% and was determined as the percentage of agreement for each cell type based on 'present' or 'absent' call for the total number of IHC and mRNA determinations. Concordance between IHC and LCM Affymetrix gene array for stromal cells was 39%. Concordance between IHC and LCM Agilent gene array was 25% for stromal cells and 36% for luminal cells.

To determine correlations between disparate methods of assessing gene expression levels, we selected a cohort of CD antigens as a reference gene set and compared transcript abundance measurements acquired by microarray hybridization with protein abundance measurements determined by immunohistochemistry in specific cell types found in the human prostate gland. The microarray and immunostaining results for each cell type are summarized in Figure 1. All annotations are deposited in a public database [14]. The sample data conform to the Minimum Information Specification For In Situ Hybridization and Immunohistochemistry Experiments (MISFISHIE) standards [15], and describe the tissue, distribution of reaction product in the tissue, localization pattern within cell types, and provide an assessment of the level of protein expression based on the immunostaining data. Our initial criteria for assessing expression centered on using 'present' *vs*. 'absent' call for both Affymetrix and Agilent array analyses. Based on this qualification, CD antigen expression for MACS-sorted cell populations agreed with the following frequency to IHC data by cell type: endothelial = 55%, luminal = 64%, stromal = 46%, basal epithelial = 63%, stem = 64%, cancer = 68%. CD antigen expression for LCM cells analyzed by Affymetrix array agreed 39% for stromal cells with IHC data, and analyzed by Agilent array agreed 25% for stromal cells and 36% for luminal cells.

**Figure 2:**
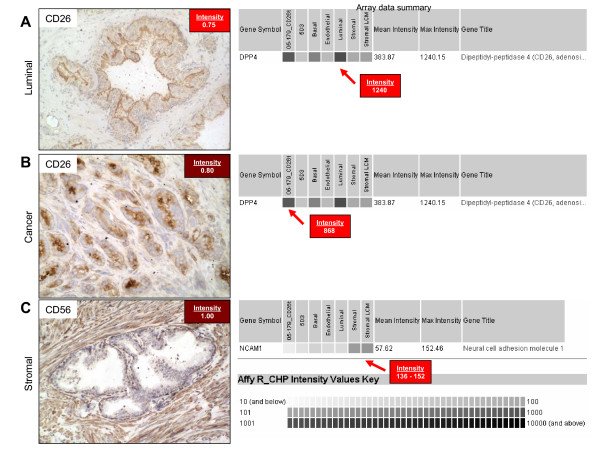
**Data-type concordance for CD26 and CD56**. CD immunoreactivity of prostate cells (immunoreaction product red-brown; pale blue hematoxylin nuclear counterstain) and corresponding gene expression data (darker shading of the boxes indicates higher mRNA levels). **A. **CD26 staining in normal prostate tissue was confined to luminal cells with a staining intensity of 0.75, this correlated well with array data summary where a maximum gene expression level of 1240 was found for luminal cells. **B. **CD26 staining in prostate cancer was restricted to cancer cells with a staining intensity of 0.80, this correlates with array data where a maximum gene expression level of 868 was found for the cancer cells. **C. **CD56 staining in normal prostate tissue was confined to stromal cells with a staining intensity of 1.00, this agreed with array data where maximum gene expression level of 136 was found for stromal cells. Original magnification: 200×.

We next sought to establish a more quantitative relationship between CD immunoreactivity of prostate cells and corresponding gene expression data (Figure 2). For example, staining for CD26 (DPP4) in normal (A) and cancer (B) prostate tissue shows clear luminal staining, which corresponds with intense gene expression measured by array in the luminal and cancer (05-179_CD26t) cell populations. The greyscale gradient indicates robust multi-array average (RMA) normalized Affymetrix signal intensity. Signals of 10 or less are represented as white and signals greater than or equal to 10,000 are represented as black. Higher Affymetrix signal (more black) indicates higher levels of gene expression. CD26 expression determined by Agilent array in LCM luminal cells was also in concordance (data shown in Figure 1). Immunostaining with CD56 (NCAM1) showed clear stromal staining and corresponding gene expression levels in both the MACS-sorted and LCM stromal cell populations. Agilent array analysis results for CD56 were 'absent' in luminal and 'present' in stromal cells as well (data shown in Figure 1).

**Figure 3:**
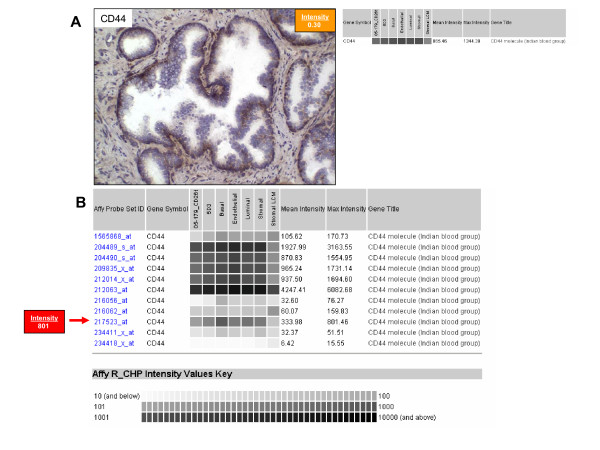
**Data-type discordance for CD44**. CD44 immunoreactivity of prostate cells and corresponding gene expression data. **A. **CD44 staining in normal prostate was confined to basal cells with a staining intensity of 0.30, this contrasted with array data where gene expression levels were similar across all cell types. Original magnification: 200×. **B. **Examination of individual probesets showed that 217523_at was most similar to immunostaining results with a maximum gene expression level of 683 for basal cells.

Data type discordance is typified by CD44 (Figure 3). Immunostaining with CD44 (G44-26, BD PharMingen, San Diego, CA), was confined to the basal cells (A), however, measured gene expression was high in all cell types (B). This antibody recognizes the epitope 1 of the standard variant of CD44 antigen. Affymetrix has 11 probe sets for CD44, showing variable expression profiles, of which only probe set 217523_at showed agreement with IHC results. CD44 has a wide range of splice variants [16] which may account for the discrepancy. Although microarray experiments detect mRNA, and it is mRNA that has the direct relationship with protein, the methodology and algorithms for Affymetrix data analysis are gene-based. Gene level annotation fails to discriminate multiple mRNAs transcribed from the same gene [17] such as is the case with CD44 [18]. Affymetrix probesets for CD44 were analyzed with Basic Local Alignment Search Tool (BLAST). Probesets 204489_s_at, 204490_s_at, 209835_x_at, and 212063_at map directly to multiple variants of CD44, whereas the other probesets map to individual variants. It is likely that gene level annotation compared to protein immunostaining will be less straightforward in cases such as CD44, where multiple protein isoforms are generated from a single gene.

**Figure 4:**
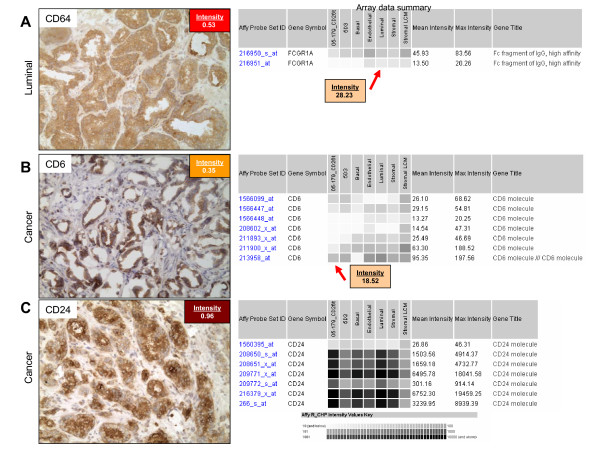
**Data-type discordance for CD64, CD6 and CD24**. CD immunoreactivity of prostate cells and corresponding gene expression data. **A. **CD64 staining of normal prostate was confined to luminal cells with a staining intensity of 0.53, contrasting with the array data where gene expression levels were absent across all cell types and for all probe sets. **B. **CD6 staining in prostate cancer was confined to cancer cells with a staining intensity of 0.35, in contrast with the array data where gene expression levels were absent across all cell types and for all probe sets with the exception of probe set 213958_at, where cancer, endothelial and luminal expression were present. **C. **CD24 staining in prostate cancer was confined to luminal cells with a staining intensity of 0.96, this contrasts with the array data where gene expression levels were present across all cell types and for all probesets with the exception of probeset 1560395_at, where expression levels were absent for all cell types. Original magnification: 200×.

Further examples of data discordance for CD64 (FCGR1A), CD6 and CD24 expression are shown in Figure 4. For CD64, immunostaining identified the luminal cell population, but measured gene expression was low or absent for all cell types regardless of probeset (A). This was also the case for CD6 (B). CD6 shows equivocal staining of luminal cells. Examination of CD24 immunostaining identifies the cancer cells, however measured gene expression was high across all cell types for some probe sets (i.e. 209771_x_at) and absent for others (i.e. 1560395_at). Gene expression in LCM samples was also discordant (data shown in Figure 1).

### Correlation of immunolocalization with array data

Correlations between IHC staining intensity and gene expression levels determined by Affymetrix array for MACS-sorted cell populations are summarized in Table 1. Statistical Pearson correlation ranged from -0.01 – 0.55 and Spearman Coefficients ranged from 0.00 – 0.51. There was no positive overall correlation of immunolocalization data with array data for endothelial, stromal or progenitor cells, and moderate positive correlation for luminal, basal and cancer. Scatter plots are shown in Additional File 1. It has been suggested that under conditions where expression differences are not dramatic, problems related to probe and probe set identity could lead to significant errors [19]. Using updated probe set definitions provided by Dai et al. [19], statistical correlations ranged from 0.09 – 0.57 and Spearman Coefficients ranged from 0.03 – 0.63 and are summarized in Table 2. Updated probe set definitions increased correlation for all cell types except for cancer cells (0.45 – 0.42). The greatest improvement was a 2-fold increase for progenitor (Spearman 0.22 – 0.41). Scatter plots are shown in Additional File 2. In summary, for either probe set definition, there was no positive correlation of immunolocalization with transcriptome data for endothelial or stromal cells, and moderate positive correlation for luminal, basal, and cancer. Updated probe set definitions increased correlation from none to moderate positive correlation for progenitor cells.

**Table 1:** Correlation between immunostaining intensity and mRNA level for each prostate cell type

	**Median**		**Mean**	
		
**Cell type**	**Spearman coefficient**	**Pearson**	**Spearman coefficient**	**Pearson**
CD31 Endothelial	0.25	0.28	0.26	0.28
CD26 Luminal	0.46	0.47	0.51	0.55
CD49a Stromal	-0.00	-0.01	0.06	0.07
CD104 Basal	0.35	0.31	0.42	0.34
ABCG2 Progenitor	0.22	0.17	0.34	0.23
CD26 Cancer	0.45	0.49	0.49	0.50

**Table 2:** Correlation between immunostaining intensity and mRNA level using updated probeset definitions for each prostate cell type

	**Median**		**Mean**	
		
**Cell type**	**Spearman coefficient**	**Pearson**	**Spearman coefficient**	**Pearson**
CD31 Endothelial	0.43	0.57	0.28	0.27
CD26 Luminal	0.57	0.61	0.57	0.63
CD49a Stromal	0.09	0.03	0.09	0.03
CD104 Basal	0.49	0.45	0.49	0.45
ABCG2 Progenitor	0.41	0.33	0.41	0.34
CD26 Cancer	0.42	0.49	0.42	0.49

### LCM confirmation

Correlations between IHC staining intensity and gene expression determined by Affymetrix and Agilent array for LCM cells are summarized in Table 3. Statistical Pearson correlation ranged from 0.26 – 0.57 and Spearman Coefficients ranged from 0.25 – 0.43. There was no positive correlation of immunolocalization data with array data for LCM stromal cells assayed by either Agilent or Affymetrix. Pearson correlation for LCM luminal cells was 0.57 and Spearman coefficient was 0.43, which were the same values as that for MACS-sorted luminal cells using updated probe set definitions. Pearson correlation between LCM stromal cell data and MACS-sorted stromal cell data assayed by Affymetrix array was 0.68, Spearman coefficient was 0.78. Pearson correlation between LCM stromal cell data analyzed by Agilent array and Affymetrix MACS-sorted stromal cell data was 0.65, Spearman coefficient was 0.55. Pearson correlation between LCM stromal cell data analyzed by Agilent array and Affymetrix LCM stromal cell data was 0.33, Spearman coefficient was 0.59. Scatter plots are shown in Additional File 3.

**Table 3:** Correlation between immunostaining intensity and mRNA level for LCM samples.

	**Mean**	
	
**Cell type**	**Spearman coefficient**	**Pearson**
CD26 Luminal	0.43	0.57
Agilent		
CD49a Stromal	0.26	0.26
Affymetrix		
CD49a Stromal	0.25	0.28
Agilent		

## Discussion

In this study, we analyzed the gene and protein expression of 58 informative CD antibodies in prostate tissue to determine the degree of concordance between immunohistochemical assessments of protein abundance and microarray-based measures of transcript levels. Based on previous characterization of prostate cancer by CD phenotype [13,20], we were interested in the correlation with corresponding cell type-specific transcriptome data and the relationship between MACS-sorted cell and laser capture microdissected populations. The CD antibodies used for comparison were selected based on their previously determined cell type specificity in prostate tissue [20]. For this study, there was good agreement between CD phenotype characterized by immunostaining and gene expression level based on 'absent' or 'present' call. Quantitative comparison of expression levels mirrored these results with correlation being lowest for stromal cells and highest for luminal cells and cancer cells. There was moderate positive correlation between measured gene expression level and immunostaining intensity for luminal, basal and cancer. There was moderate positive correlation for progenitor cells using updated probe set definitions.

Previous work in our lab has indicated that Affymetrix 'present' calls are approximately 90% accurate, however, 'absent' calls are only 40% accurate [21]. Most often genes classified by array analysis as absent were, in fact, expressed when measured by more sensitive RT-PCR methods, but were of very low abundance. With respect to characterizing CD expression by immunostaining, there were few cases where CD expression was scored by immunostaining and not by gene arrays. In fact, it was more likely that CD genes classified as present by array were not immunostained by that CD antibody. There are several possible explanations for these discrepancies. Biological reasons for poor correlation include post-transcriptional and post-translational modifications, as well as the possibility that proteins have very different half-lives [22,23]. Recent analyses indicate that protein concentrations correlate with the corresponding mRNA levels by only 20 – 40%, and that mRNA abundances are only a weak surrogate for corresponding protein concentration [24,25]. Further complicating matters is the potential for errors and noise in protein and mRNA experiments. Although we have previously documented the advantages of MACS-sorted cell over whole tissue transcriptomes [13] gene expression may be affected by collagenase tissue digestion and cell isolation processes. To examine this possibility we compared array results from MACS-sorted cells with LCM cells. Results for luminal cells were virtually the same for LCM and MACS-sorted cells, however for stromal cells, correlation increased about 3-fold (from 0.08 in MACS-sorted cells to 0.26 in LCM indicating that for stromal cells the non-specific detection is due to low levels of contaminating cells in the MACS-sort cells. Gene expression analysis is much more sensitive than immunohistochemistry, but it may also be that genes expressed are at levels not high enough for translated protein expression. Satisfactory immunostaining of surface membrane antigens requires preservation of protein tertiary structure in the region of the relevant epitopes [26] and it may be that some of these CD antibodies are less immunoreactive than others. Additionally, some mRNAs may cross-hybridize probes in the array that are designed to detect another mRNA, or probes that are designed to detect the mRNA of a particular gene may be relying on genomic expressed sequence tag (EST) information that is incorrectly associated with that gene. Clearly there are difficulties associated with some antigens such as those we found in CD44, CD64 (FCGR1A), CD6 and CD24 where probe set variability indicates that IHC is a better method for analysis at this time. The use of updated probe set definitions slightly increased correlations for all cell types indicating that inaccurate probe sets may account for some of the disparities found between the two techniques in this study. For studies involving the prostate, suggested targets for CD profiling using the combination of DNA array and IHC technologies are listed in Table 4.

**Table 4:** Suggested CD profiling targets with good agreement between data types for prostate characterizations.

**Cell type**	**CD antigens 'present'**	**CD antigens 'absent'**
CD31 Endothelial	31, 49f, 54, 58, 59, 73, 74, 90, 107a, 107b, 144	1a, 6, 10, 30, 32, 41, 43, 56, 66f, 70, 75s, 84, 91, 94, 179a, 226, 271
CD26 Luminal	9, 10, 13, 26, 38, 63, 75s, 98, 107a, 107b, 147, 164	1a, 6, 30, 32, 41, 43, 49c, 50, 56, 66f, 70, 74, 84, 88, 91, 94, 104, 153, 179a, 226, 271
CD49a Stromal	29, 47, 49a, 56, 59	1a, 6, 26, 30, 32, 41, 43, 50, 64, 66f, 70, 75s, 84, 91, 94, 104, 153, 179a, 271
CD104 Basal	9, 44, 47, 49b, 49f, 59, 74, 92, 95, 104, 107b, 138, 147, 271	1a, 6, 30, 32, 41, 43, 49c, 50, 56, 64, 66f, 70, 75s, 84, 91, 94, 153, 179a, 226
ABCG2 Progenitor	9, 44, 47, 49b, 49f, 59, 74, 92, 95, 138, 147	1a, 6, 30, 10, 13, 26, 30, 32, 41, 43, 49c, 50, 56, 64, 66f, 70, 84, 91, 94, 153, 179a, 226
CD26 Cancer	9, 24, 26, 29, 38, 40, 46, 49b, 63, 75s, 147	10, 13, 30, 32, 41, 43, 49c, 50, 56, 62L, 66f, 84, 88, 90, 91, 94, 104, 144, 153, 179a, 226, 271

'Present' denotes the list of CD antigens expressed by the specific cell type and detected as by both array and IHC. 'Absent' cites the list of CD antigens undetected by both array and IHC.

Many cross-platform studies have been conducted on DNA microarrays. These previous studies have discovered several factors that effect data comparison across different microarray platforms. In addition to our findings, updated annotation through RefSeq or UniGene databases has repeatedly been shown to increase cross-platform content and consistency [27-31]. Biological variability, and inter-laboratory variation have also been previously implicated as sources of cross-platform data discrepancies [32-34]. For this study, although conducted by two different laboratories, the correlation between the two platform types (Affymetrix and Agilent) was only different for prostate stromal cells. Therefore, it is probable that these discrepancies were due to varying sampling methods (e.g. MACS-sorting of digested tissue *vs*. LCM of frozen tissue sections). Additionally, discrepancy could be due to the differing tissue histology of the prostate and therefore possible differing purity for stromal *vs*. luminal cell types. The prostate stroma is fairly heterogenous, consisting of smooth muscle cells and fibroblasts, with embedded blood vasculature, peripheral nerves and ganglia, and tissue infiltrating white blood cells; whereas the prostate luminal layer contains luminal secretory cells and rare neuroendocrine cells.

## Conclusion

In summary, CD molecules can be used to isolate cell populations from prostate tissue. These cell populations retain to a high degree their CD phenotype as determined by immunostaining in intact tissue. Observed discrepancies were mostly in expression levels detected by microarray and not immunohistochemistry; these differences are possibly due in part to varying sampling methods, problems related to probe and probe set identity as well as actual biological differences. It is hoped that continued improvements in cell sorting techniques and probe set development could further elucidate the relationship between mRNA levels and corresponding protein expression. In addition to comparison of immunohistochemistry and microarray data, there is further need for cross-comparison of results utilizing techniques such as in situ hybridization, western blotting and high throughput proteome analysis. Future studies analyzing mRNA and protein correlation will be required to determine the potential bias of various experimental methodologies as well as how mRNA expression differences are related to protein expression.

## Methods

### Prostate tissue

The methods of tissue collection, immunostaining and expression data collection and analysis used in this study have been published previously [13,20,35]. Briefly, tissue samples for IHC and Affymetrix array analysis consisted of both cancer-enriched and cancer-free samples obtained from 55 radical prostatectomies. Upon receipt of prostatectomy specimens, 3-mm thick transverse sections were made of the prostate after inking the exterior surface (the surgical margin). The same approach was used for both cancer-free and cancer-enriched (where at least 85% of the cells in the corresponding frozen section were cancer cells) samples. Between 4 and 6 blocks of tissue from the posterior aspect (the peripheral zone) of each prostate were frozen. Sections from the frozen blocks of tissue provided a histological template for characterizing the cell composition of the adjacent non-frozen tissue blocks. Cancer-free samples, weighing between 2 and 10 g, were harvested primarily from the anterior aspect of the prostate (transition zone) as described [12,13,35]. Cancer-enriched samples, weighing at least 100 mg, were dissected from the opposing aspect of the non-fixed section adjacent to the block of tissue that had been frozen. Tissue specimens for cell sorting were digested by collagenase [36,37].

As an external control, IHC and array data from MACS-sorted cells were compared to LCM cells from prostate stromal and luminal cells analyzed by hybridization to long-oligonucleotide microarrays (Agilent Corporation). Additionally, LCM stromal cells were analyzed by Affymetrix array to control for potential platform variation. Tissue specimens for LCM were obtained from prostate needle biopsies from 14 men identified from a biospecimen repository with no evidence of cancer as well as a second or third set of biopsies that also showed no evidence of carcinoma. Each biopsy specimen was immediately embedded in OCT and flash frozen in isopentane immersed in liquid nitrogen. All protocols for tissue acquisition and processing were under approval by the University of Washington Institutional Review Board following a standard protocol.

### Immunohistochemistry

Blocks of unfixed prostate tissue were harvested at surgery. Serial 5-μm sections were prepared from randomly selected frozen blocks, fixed in cold acetone, and processed for immunohistochemistry. We used frozen sections since a majority of the anti-CD antibodies we used do not immunoreact with antigens in formalin-fixed, paraffin-embedded tissue. Immunohistochemistry was performed as described previously [20]. The primary antibodies used were mouse monoclonal CD antibodies (BD-PharMingen, San Diego, CA) diluted to 8 ng/μl or less. Antigen was localized using biotinylated anti-mouse IgG or IgM (Vector Labs, Burlingame, CA) as the secondary antibody and diaminobenzidine tetrahydrochloride as the chromogen. The sections were counterstained with hematoxylin.

The percentage of cells of a specific histological phenotype that expressed the antigen was estimated in five randomly selected fields at a final magnification of 40× (ocular 10×; objective 4×). Staining intensity was evaluated by two parameters (staining intensity and percentage of cells exhibiting each level of intensity). Intensity of reaction product was based on a 3-point scale – none, faint/equivocal and intense. These categories of staining intensity were defined as follows. *Intense*: immunoreaction deposit is distinctly more optically dense than background and than tissue that does not express the antigen. *Equivocal*: immunoreaction deposit is either similar enough in optical density to the background and/or to tissue that does not express the antigen, or is so focal, i.e. < 5% of cells, that there is reasonable uncertainty regarding whether the cells express the antigen. *None*: there is either no immunoreaction deposit or reaction product is no more optically dense than background.

A single value *A *was calculated for each immunostain by cell type using the following formula:

$$A=\frac{\text{0(\% no stain)}+\text{WF(\% faint/equivocal stain)}+\text{1(\% convincing stain)}}{\text{100}}$$

WF = weighting factor, usually 0.2

The value of the weighting factor can be changed on our web site by independent observers of the images. We did this since we thought that observers of the images should be able to alter the value assigned to the weighting factor based on whether they thought that faint/equivocal immunostained cells either did or did not truly represent immunolocalization of each respective CD antigen. For the present study we used a weighting factor of 0.2.

An average score for each cell type for each CD antigen was calculated using the following equation:

$$Score=\frac{1}{n}\sum_{i=1}^{n}A_{i}$$

where:

*n *= number of immunostains for each CD by cell type

*A *= staining value for each immunostain by cell type

Average scores in the range of 0.751 to 1.0 were categorized as *intense*, 0.251 to 0.750 as *equivocal *or 0 to 0.250 as *none*. Immunostained sections were imaged with an Olympus BX41 microscope (Olympus, Melville, NY) equipped with a MicroFire digital camera (Optronics, Goleta, CA). Composite images were constructed with Photoshop CS (Adobe Systems, San Jose, CA) and are publicly available online [38].

### Affymetrix gene expression profiling of MACS-sorted cell populations and laser captured stromal cells

Transcriptomes of the following prostate cell types have been previously determined: CD104^+ ^basal epithelial cells, CD26^+ ^luminal epithelial cells, CD49a^+ ^stromal fibromuscular cells, and CD31^+ ^endothelial cells [13], ABCG2^+ ^stem cells [39] and CD26^+ ^cancer cells [in preparation]. The CD molecules cited above were the ones used for sorting each specific cell type for gene expression profiling. The expression of genes documented in the literature as cell-type specific was compared to Affymetrix datasets; analyses showed that the transcriptomes were representative of their respective cell type (p < 0.05) [13]. Five separate biological replicates of each MACS-sorted cell populations (except the CD26^+ ^cancer cells which comprised of one biological sample) and three separate biological replicates of laser captured stromal cells were assayed to produce a dataset using the Human Genome U133 Plus 2.0 GeneChips (Affymetrix, Santa Clara, CA). The U-133 Plus 2.0 array contains probesets representing 54,675 genes, splice variants, and ESTs. The GeneChips were prepared, hybridized, and scanned according to the protocols provided by Affymetrix. Briefly, 200 ng of RNA was reverse transcribed with poly (dT) primer containing a T7 promoter, and the cDNA was made double-stranded. In vitro transcription was performed to produce unlabeled cRNA. Next, first-strand cDNA was produced with random primer. cDNA was made double-stranded with poly (dT) primer/T7 promoter. Finally, in vitro transcription was performed with biotinylated ribonucleotides. The biotin-labeled cRNA was hybridized to the GeneChips. The chips were washed and stained with streptavidin-PE using an Affymetrix FS-450 fluidics station. Data was collected with an Affymetrix GeneChip Scanner 3000.

### LCM and RNA isolation of luminal and stromal cells for Agilent array analysis

Approximately two thousand luminal epithelial or stromal cells from histologically benign glands were captured using the Veritas Microdissection System (Molecular Devices Corporation, Sunnyvale, CA) as previously described [40]. Frozen sections (8 μm) were cut from tissue embedded in OCT (Miles, Inc. Diagonostic Division, Elkhart, IN) and fixed in cold 95% ethanol. After brief (5–10 seconds) staining with hematoxylin using the HistoGene staining solution (Arcturus Engineering, Mountain View, CA), the sections were dehydrated in 100% ethanol, followed by sequential ethanol-xylene solutions. Three stromal cell samples and 14 luminal cell samples were captured. Digital photographs were taken of tissue sections before, during, and after LCM and assessed independently by two investigators to confirm that the laser-captured cells were stromal and luminal cells, respectively.

Captured individual specimens were lysed and RNA was isolated using Arcturus PicoPure RNA Isolation. Samples were treated with RNase-free DNase (Qiagen, Inc., Valencia, CA). This RNA was converted to a cDNA library and amplified using the TransPlex WTA (Rubicon Genomics, Ann Arbor, MI) using Titanium Taq polymerase (Clontech Laboratories, Inc., Mountain View, CA) in the presence of amino-allyl deoxyuridine triphosphate (dUTP) for postamplification labeling. Real-time PCR amplifications were terminated at plateau phase, as measured by fluorescence incorporation, to preserve maximum representation. Concentration and purity was determined using the ND-1000 spectrophotometer (Nanodrop Technologies, Wilmington, DE). To provide a reference standard RNA for use on 2-color microarrays, we isolated total RNA from LNCaP, DU145, PC3, and CWR22 cell lines (American Type Culture Collection, Manassas, VA) growing at log phase in dye-free RPMI-1640 medium supplemented with 10% fetal bovine serum (FBS; Life Technologies, Rockville, MD). RNA was purified using Trizol (Life Technologies). The RNA was then further purified by Qiagen RNeasy maxi and treated with Qiagen RNase-free DNase. This total RNA was then amplified using the TransPlex WTA similar to the experimental samples.

### Gene expression profiling on Agilent microarrays

Two μg of WTA-amplified amino-allyl cDNA from each of 14 luminal and 3 stromal samples were labeled with Cy3 fluorescent dye (cell line reference cDNA was labeled with Cy5) as described previously [40], and hybridized to Agilent 44 K whole human genome expression oligonucleotide microarray slides (Agilent Technologies, Inc., Santa Clara, CA) following the manufacturer's suggested protocols. Fluorescence array images were collected for both Cy3 and Cy5 using an Agilent fluorescent scanner, and Feature Extraction software (Agilent Technologies, Santa Clara, CA, USA) was used to grid, extract image intensities, and normalize data. Spots of poor quality, as determined by the software, were removed from further analysis. Data from the 14 luminal or 3 stromal samples were combined and the average signal values were used for comparative analyses. For genes with multiple probes on the array, the data from the probe with the highest sequence specificity was chosen.

### Data comparison and statistical analysis

Immunohistochemistry data was compared to Affymetrix array data for the following prostate cell types: MACS-sorted basal cells, luminal cells, stromal fibromuscular cells, endothelial cells, stem cells and cancer cells from a primary tumor of Gleason 3+3 = 6. Additionally IHC data was compared to Affymetrix array data for LCM stromal and Agilent array data for LCM stromal and epithelial cells. Data was analyzed for concordance and correlation of expression levels. Data concordance was defined as agreement between data types based on 'present' or 'absent' calls, i.e., if both techniques detected a gene, they were considered to be in agreement. Genes in the Affymetrix array dataset with an average raw fluorescence signal < 50 were considered to be undetected by the experiment and were classified as 'absent' by GCOS version 1.0 software (Affymetrix) [13]. Genes were also classified as undetected if the majority (> 50%) of individual probe sets were classified as 'absent'. All other hybridization patterns were classified as 'present'. Genes with signal 51–500 were considered to have 'low' expression, those with signal 501–5000 were considered to have 'medium' expression, and those with signal > 5000 were considered to have 'high' expression. Genes in the Agilent array dataset with an average raw fluorescence signal < 100 were considered to be 'absent', those with signal 100–999 were considered to have 'low' expression, those with signal 1000–9999 were considered to have 'medium' expression, and those with signal > 9999 were considered to have 'high' expression. Immunohistochemistry data having a staining level of *intense *(score range 0.751 – 1.0) or *equivocal *(score range 0.251 – 0.750) were classified as in agreement with genes classified as 'present'. Immunostaining results of *none *(score range 0 – 0.250) were considered equivalent to 'absent'.

The correlation between mRNA expression levels and immunostaining intensity for each CD antibody was described by Pearson product-moment and Spearman nonparametric correlation measures and tests. Correlations were calculated for mean and median values. Median was included as a more robust measurement in the presence of outlier or skewed values especially for the array data. Individual Affymetrix probes within a probe set were originally designated to hybridize with the same unique mRNA transcript. However, increasing knowledge of genomic sequences has shown that a substantial number of the manufacturer's original probe groupings and mappings are inaccurate and must be corrected [41]. To address the possibility that disparity in expression level was due to probe set definition, correlation was additionally determined using updated custom CDF definitions [19]. Dai et al., have reorganized probe set definitions based on the latest genome and transcriptome information [17]. These updated definitions based on Version 8 Entrez Gene mappings were downloaded and used in addition to the Affymetrix probe set definitions. To control for possible gene expression changes induced by the cell sorting process, LCM data for stromal cells were also compared for correlation. Additionally, LCM data for luminal and stromal cells assayed by Agilent array analysis was also used for correlation calculations. Correlations were calculated using both original and updated probe set definitions and were categorized as follows: no positive correlation (0.00 to 0.30), moderate positive correlation (0.31 to 0.79), and strong positive correlation (0.80 to 0.99).

## Authors' contributions

LEP participated in study design, experiments, and drafting of the manuscript. Data analysis and database design and programming were performed by DSC with input from EWD. MR, LJE, IMC and PSN carried out LCM experiments and data analysis. AYL conceived of study and assisted in study design and coordination. All authors have read and approved the final manuscript.

## Supplementary Material

Additional file 1 Scatter plot of IHC staining intensity and gene expression levels determined by Affymetrix array for MACS-sorted cell populations. Statistical Pearson correlation ranged from -0.01 – 0.55 and Spearman Coefficients ranged from 0.00 – 0.51. There was no positive overall correlation of immunolocalization data with array data for endothelial, stromal or progenitor cells, and moderate positive correlation for luminal, basal and cancer. Click here for file

Additional file 2 Scatter plot using updated probe set definitions. Statistical Pearson correlations ranged from 0.09 – 0.57 and Spearman Coefficients ranged from 0.03 – 0.63 using updated probe set definitions. Updated probe set definitions increased correlation from none to moderate positive correlation for progenitor cells. Click here for file

Additional file 3 Scatter plot of IHC staining intensity and gene expression levels determined by Affymetrix and Agilent array for LCM cells. Statistical Pearson correlation ranged from 0.26 – 0.57 and Spearman Coefficients ranged from 0.25 – 0.43. There was no positive correlation of immunolocalization data with array data for LCM stromal cells assayed by either Agilent or Affymetrix. Pearson correlation for LCM luminal cells was 0.57 and Spearman coefficient was 0.43, which were the same values as that for MACS-sorted luminal cells using updated probe set definitions. Pearson correlation between LCM stromal cell data and MACS-sorted stromal cell data assayed by Affymetrix array was 0.68, Spearman coefficient was 0.78. Pearson correlation between LCM stromal cell data analyzed by Agilent array and Affymetrix MACS-sorted stromal cell data was 0.65, Spearman coefficient was 0.55. Pearson correlation between LCM stromal cell data analyzed by Agilent array and Affymetrix LCM stromal cell data was 0.33, Spearman coefficient was 0.59. Click here for file
