# Supplementary material for: Correlation of mRNA and protein levels: Cell type-specific gene expression of cluster designation antigens in the prostate
Source: BMC Genomics. 2008 May 23;9:246. doi: 10.1186/1471-2164-9-246 (PMC2413246; doi:10.1186/1471-2164-9-246)
Supplement: Additional file 1 — Scatter plot of IHC staining intensity and gene expression levels determined by Affymetrix array for MACS-sorted cell populations. Statistical Pearson correlation ranged from -0.01 – 0.55 and Spearman Coefficients ranged from 0.00 – 0.51. There was no positive overall correlation of immunolocalization data with array data for endothelial, stromal or progenitor cells, and moderate positive correlation for luminal, basal and cancer. [file 1471-2164-9-246-S1.pdf]

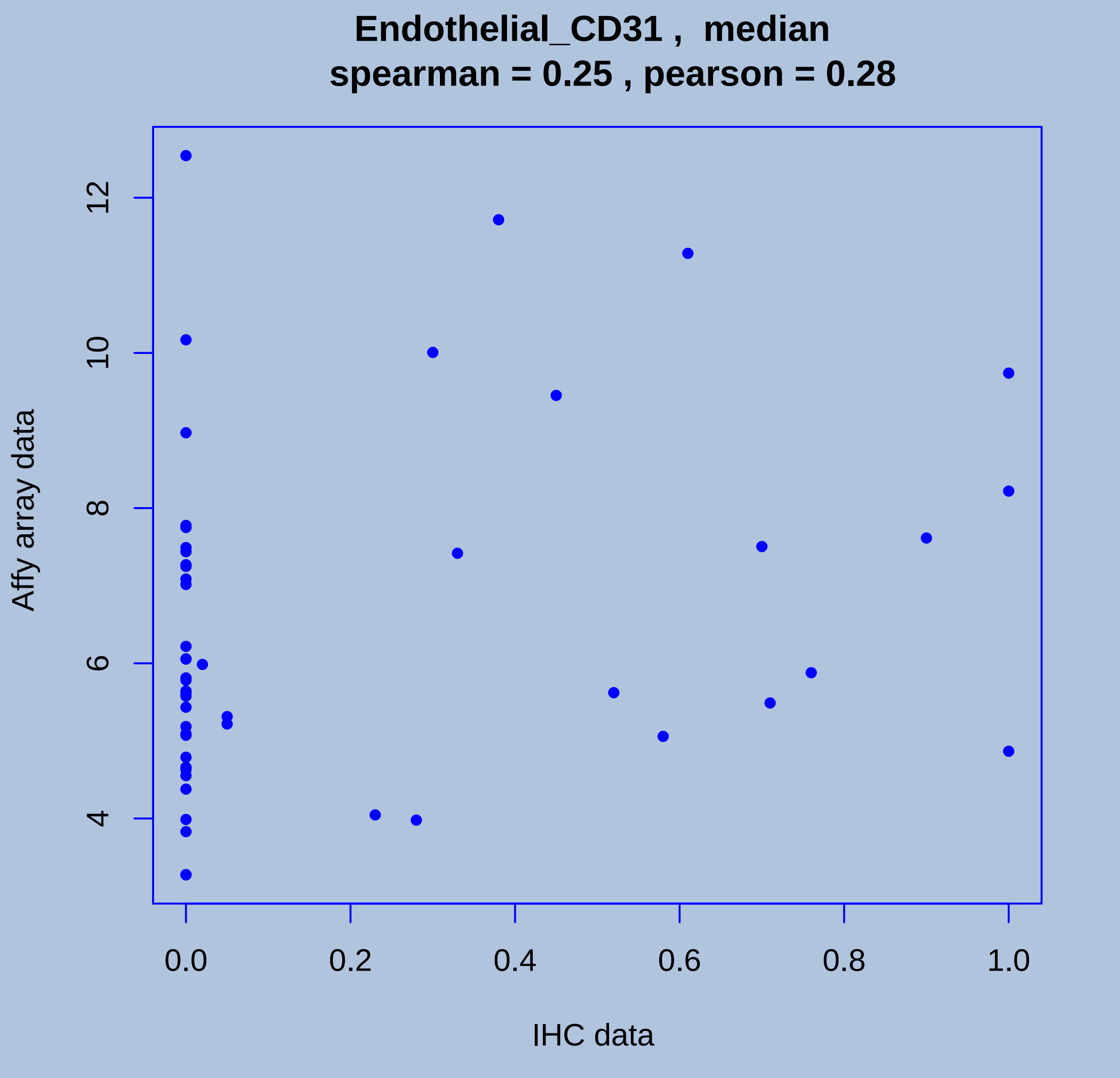
Endothelial_CD31 , median
spearman = 0.25 , pearson = 0.28
Affy array data
IHC data
4
6
8
10
12
0.0
0.2
0.4
0.6
0.8
1.0

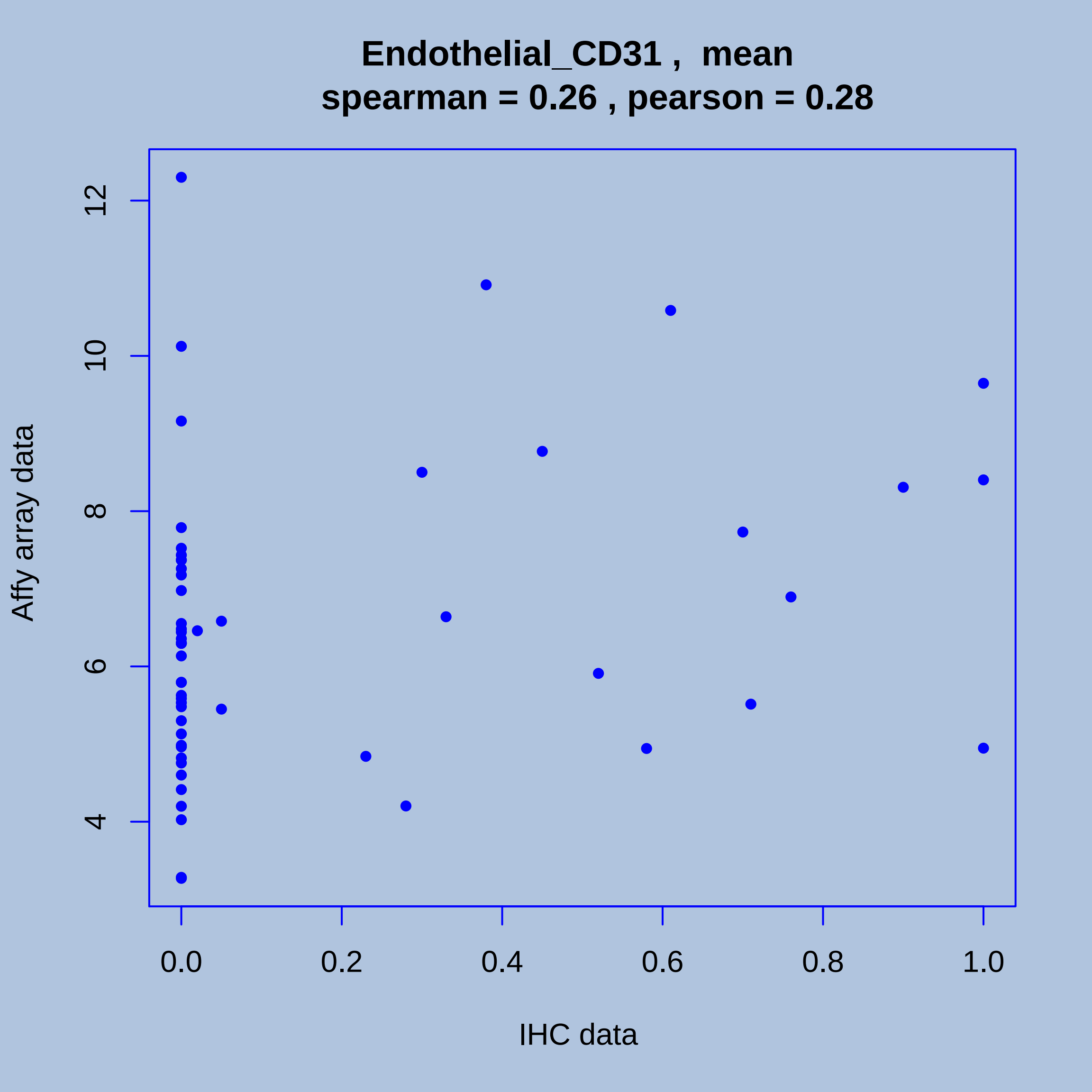

Endothelial_CD31 , mean
spearman = 0.26 , pearson = 0.28
Affy array data
IHC data
4
6
8
10
12
0.0
0.2
0.4
0.6
0.8
1.0

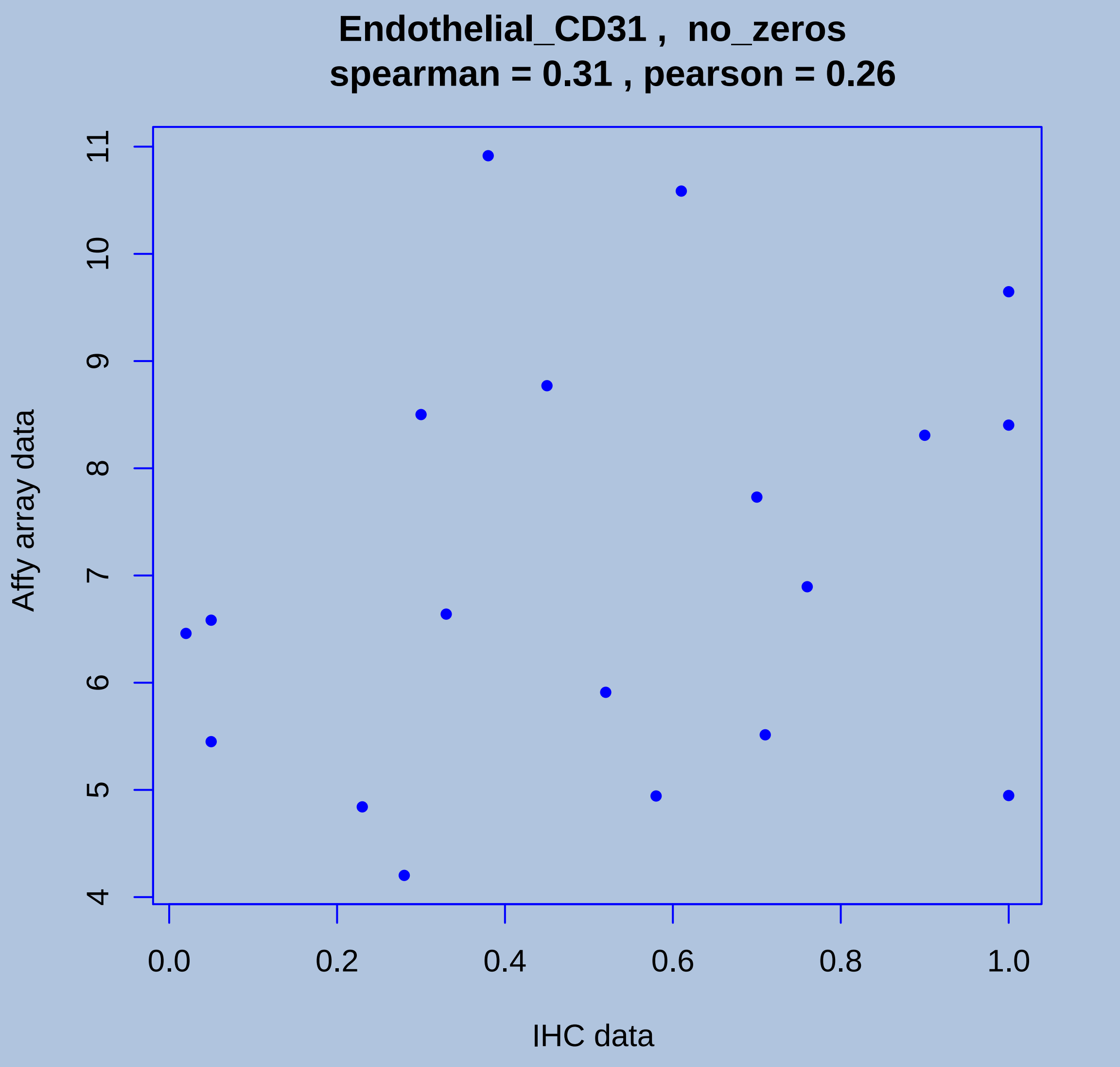
Endothelial_CD31 , no_zeros
spearman = 0.31 , pearson = 0.26
Affy array data
IHC data
4
5
6
7
8
9
10
11
0.0
0.2
0.4
0.6
0.8
1.0

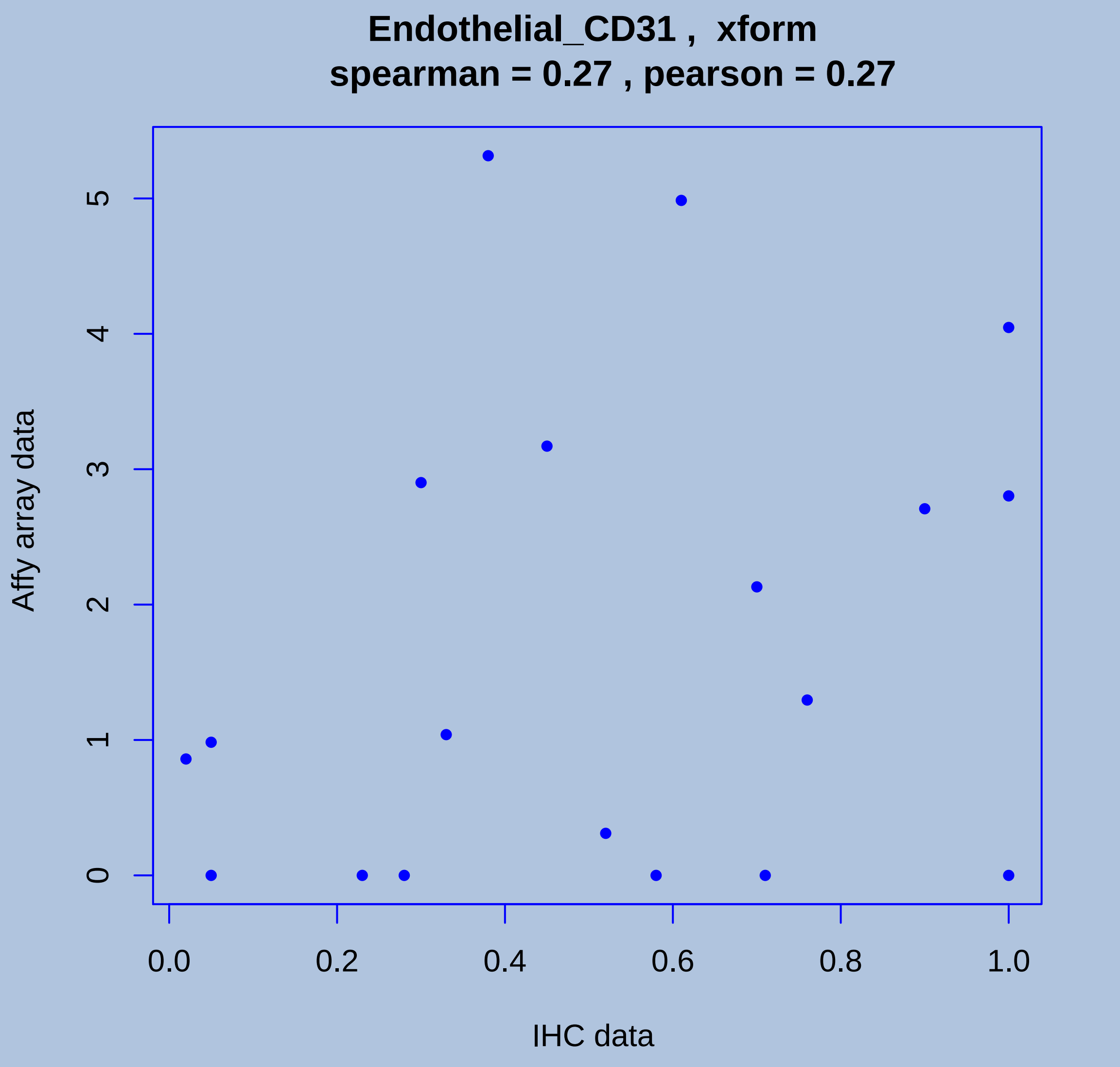
Endothelial_CD31 , xform
spearman = 0.27 , pearson = 0.27
Affy array data
IHC data
0
1
2
3
4
5
0.0
0.2
0.4
0.6
0.8
1.0

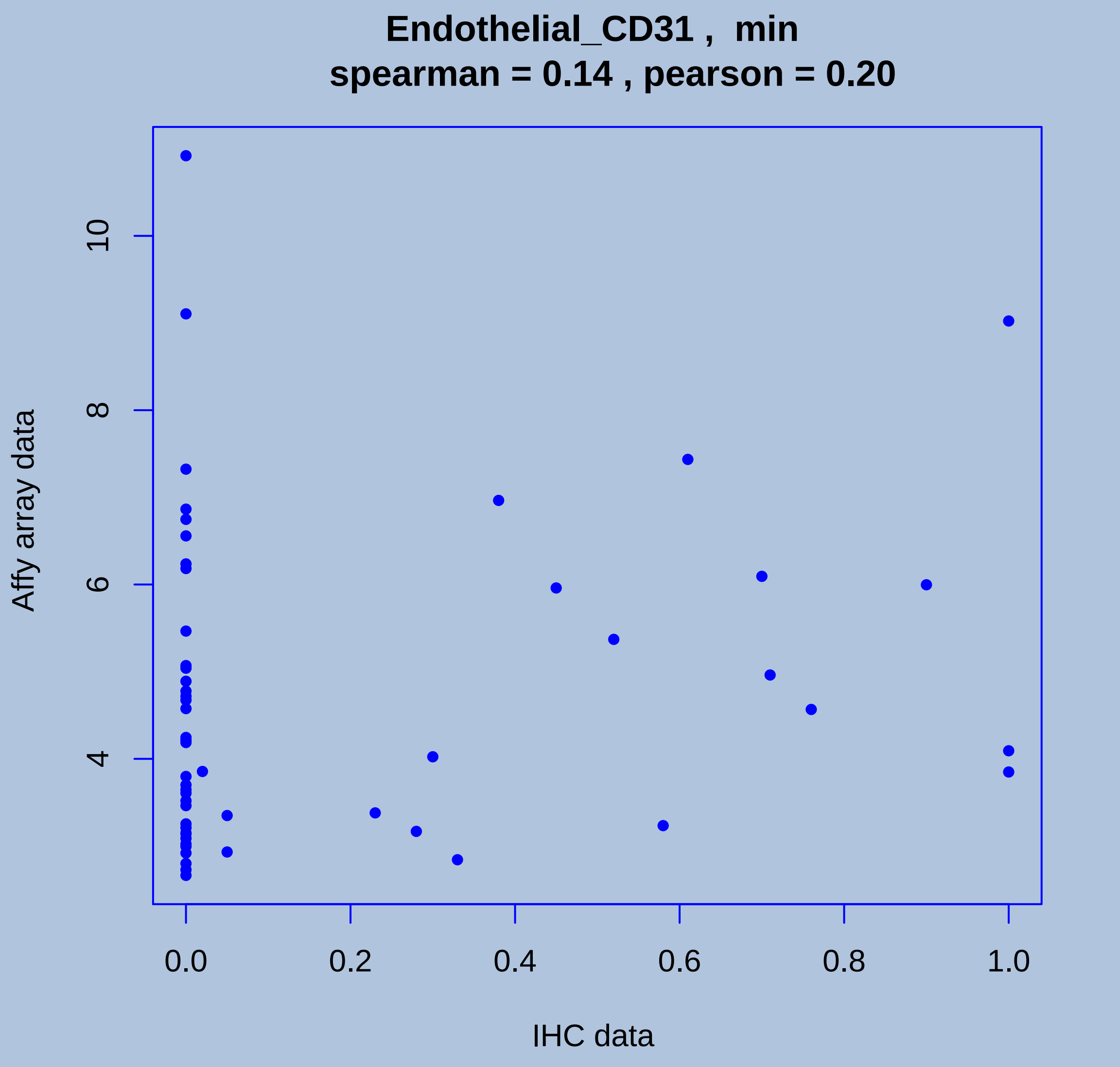
Endothelial_CD31 , min
spearman = 0.14 , pearson = 0.20
Affy array data
4
6
8
10
0.0
0.2
0.4
0.6
0.8
1.0
IHC data

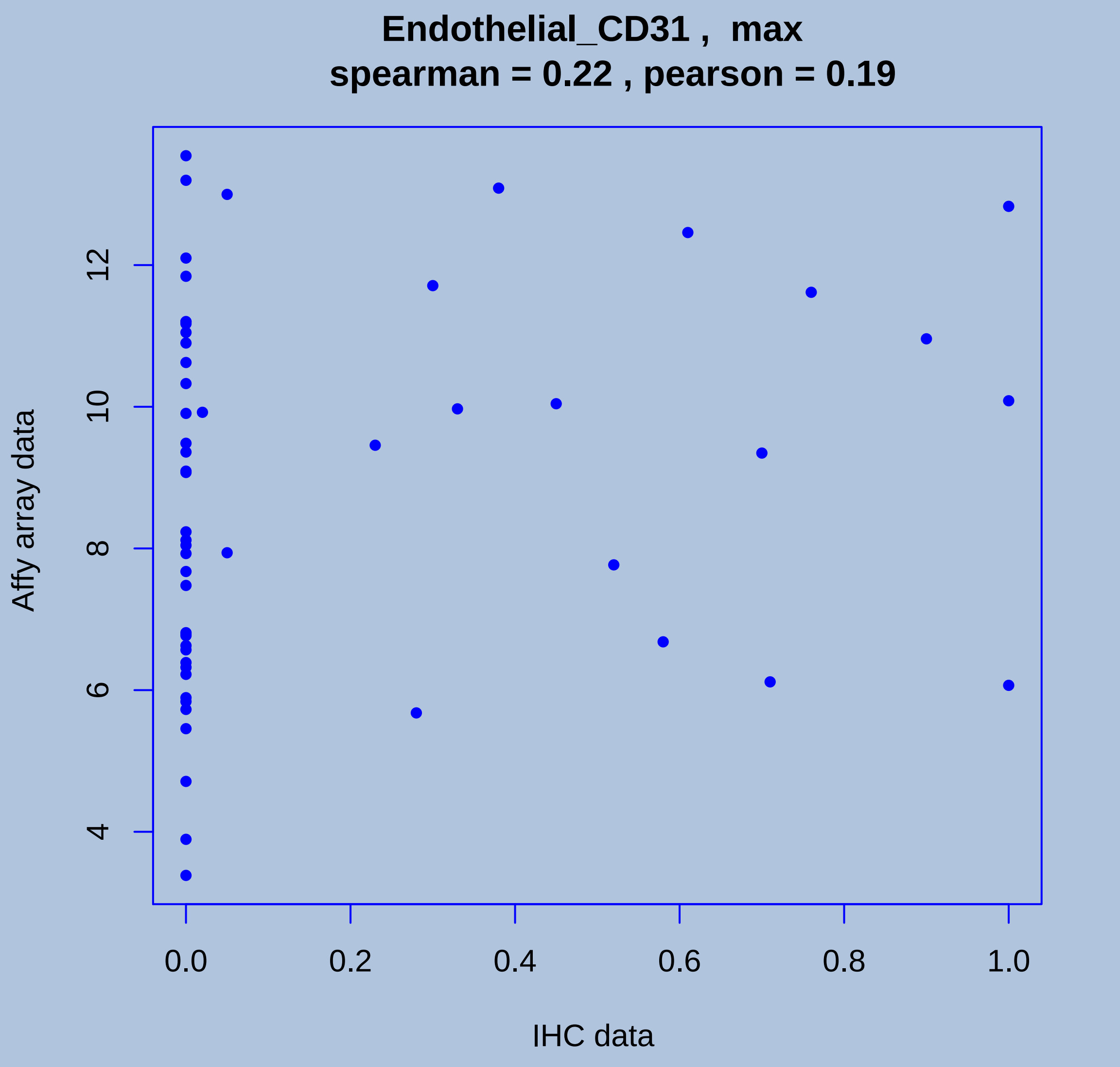
Endothelial_CD31 , max
spearman = 0.22 , pearson = 0.19
Affy array data
IHC data
4
6
8
10
12
0.0
0.2
0.4
0.6
0.8
1.0

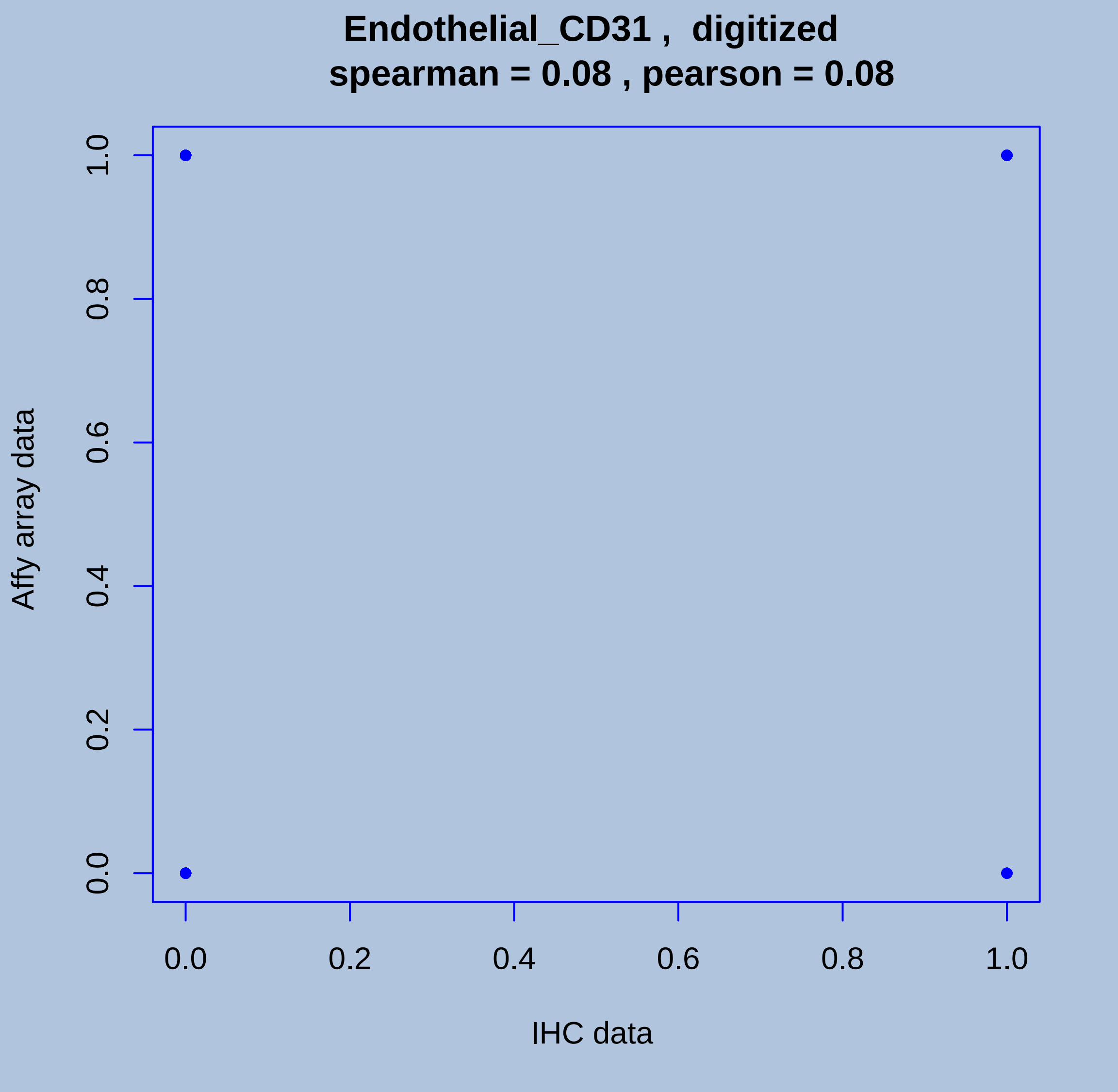

Endothelial_CD31 , digitized
spearman = 0.08 , pearson = 0.08
Affy array data
IHC data
0.0
0.2
0.4
0.6
0.8
1.0

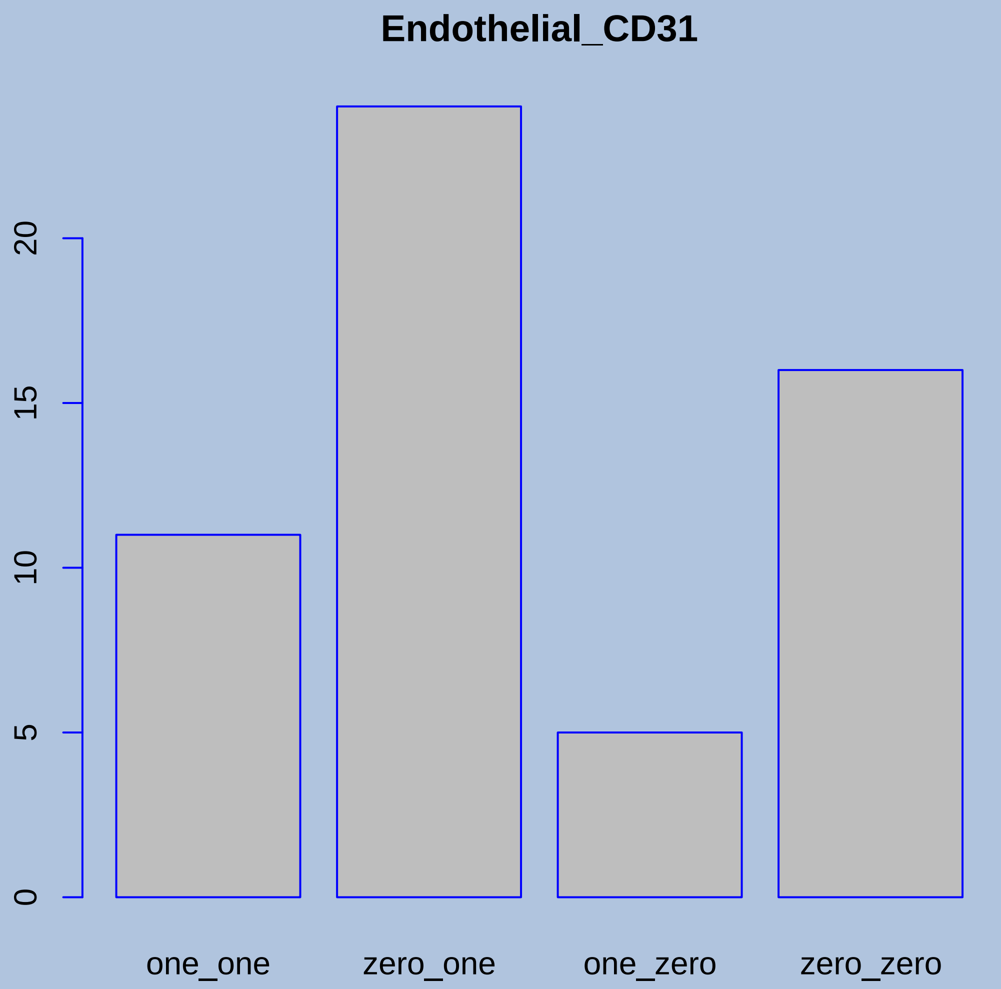
Endothelial_CD31
0
5
10
15
20
one_one
zero_one
one_zero
zero_zero

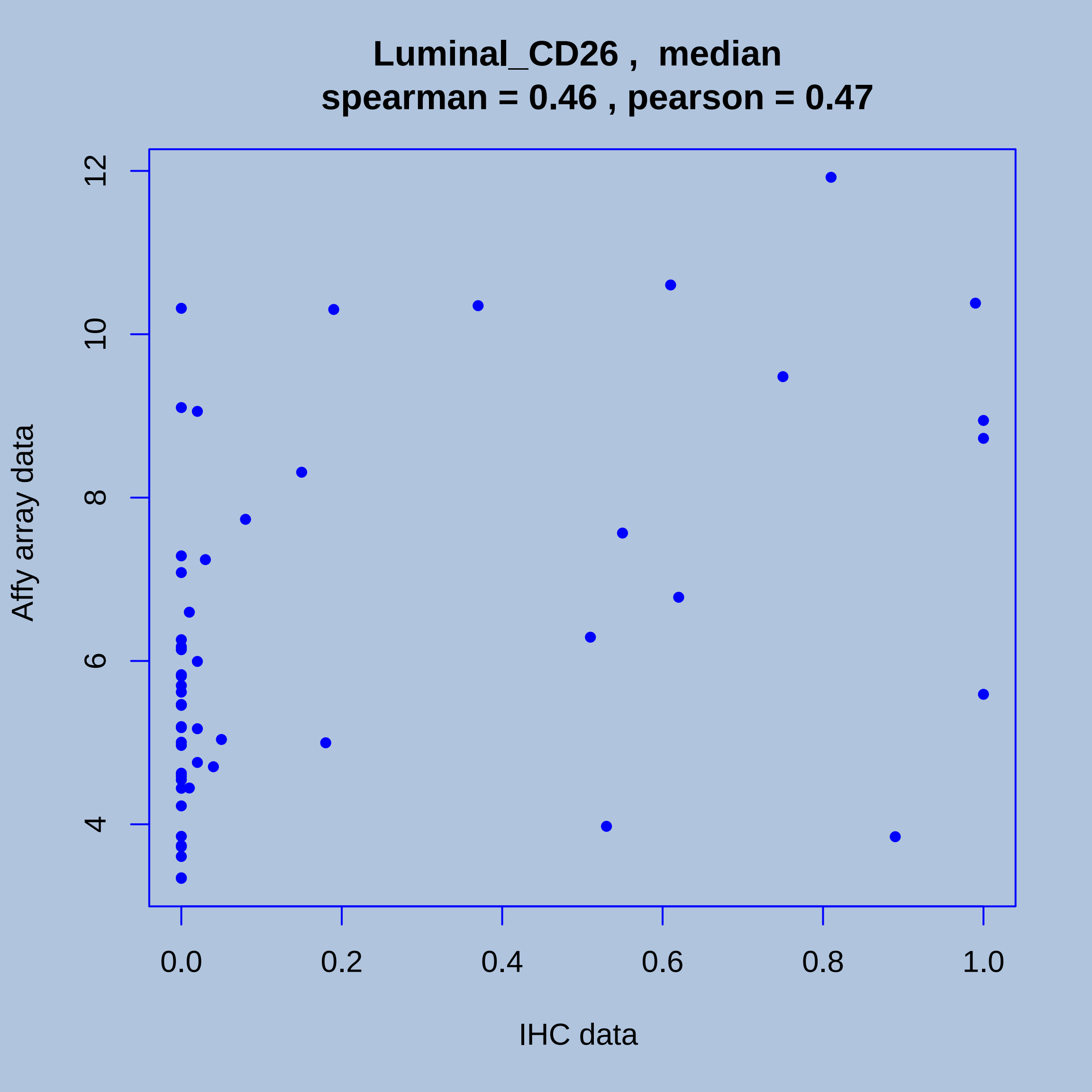

Luminal_CD26 , median
spearman = 0.46 , pearson = 0.47
Affy array data
IHC data
0.0
0.2
0.4
0.6
0.8
1.0
4
6
8
10
12

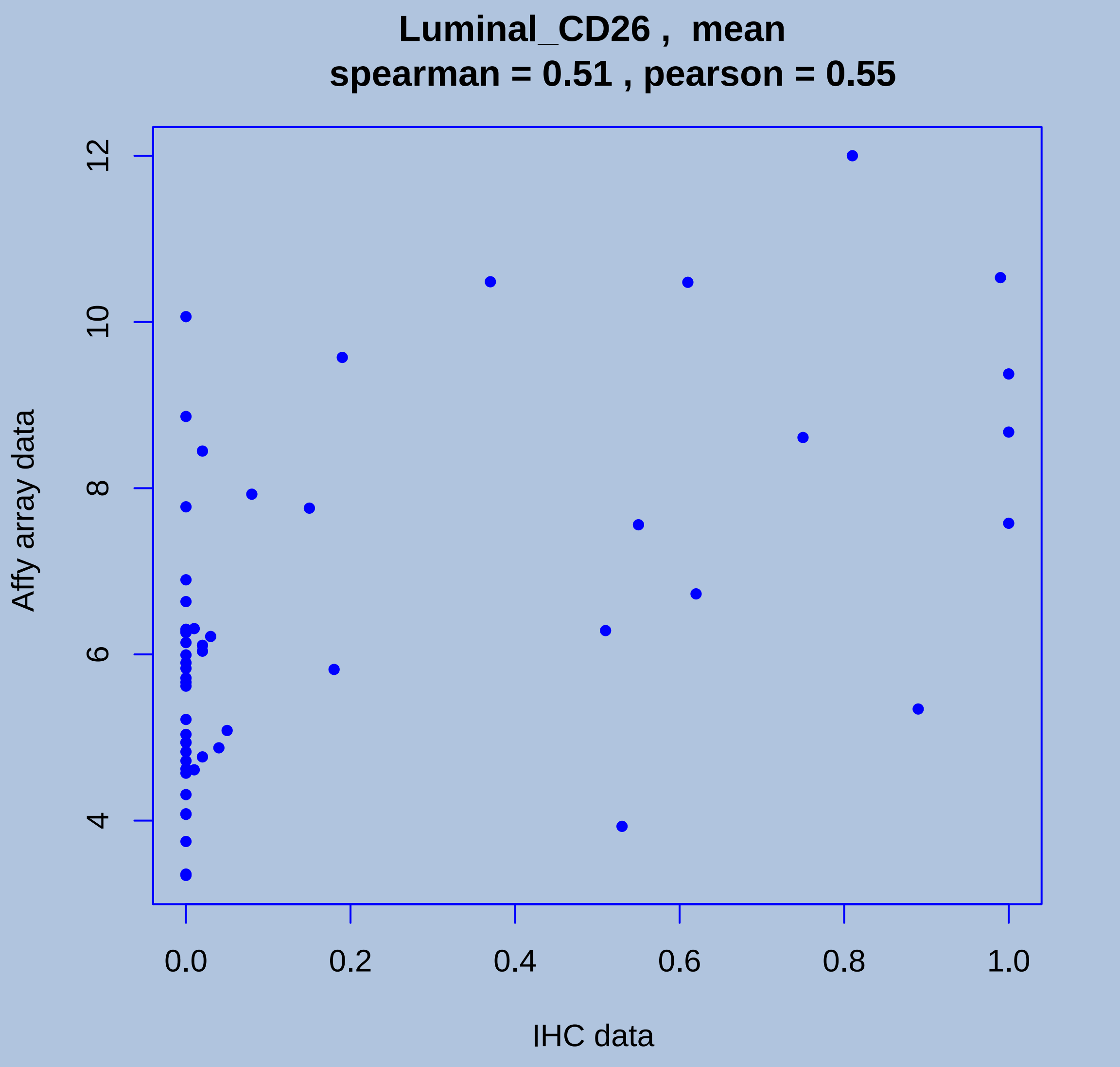
Luminal_CD26 , mean
spearman = 0.51 , pearson = 0.55
Affy array data
IHC data
4
6
8
10
12
0.0
0.2
0.4
0.6
0.8
1.0

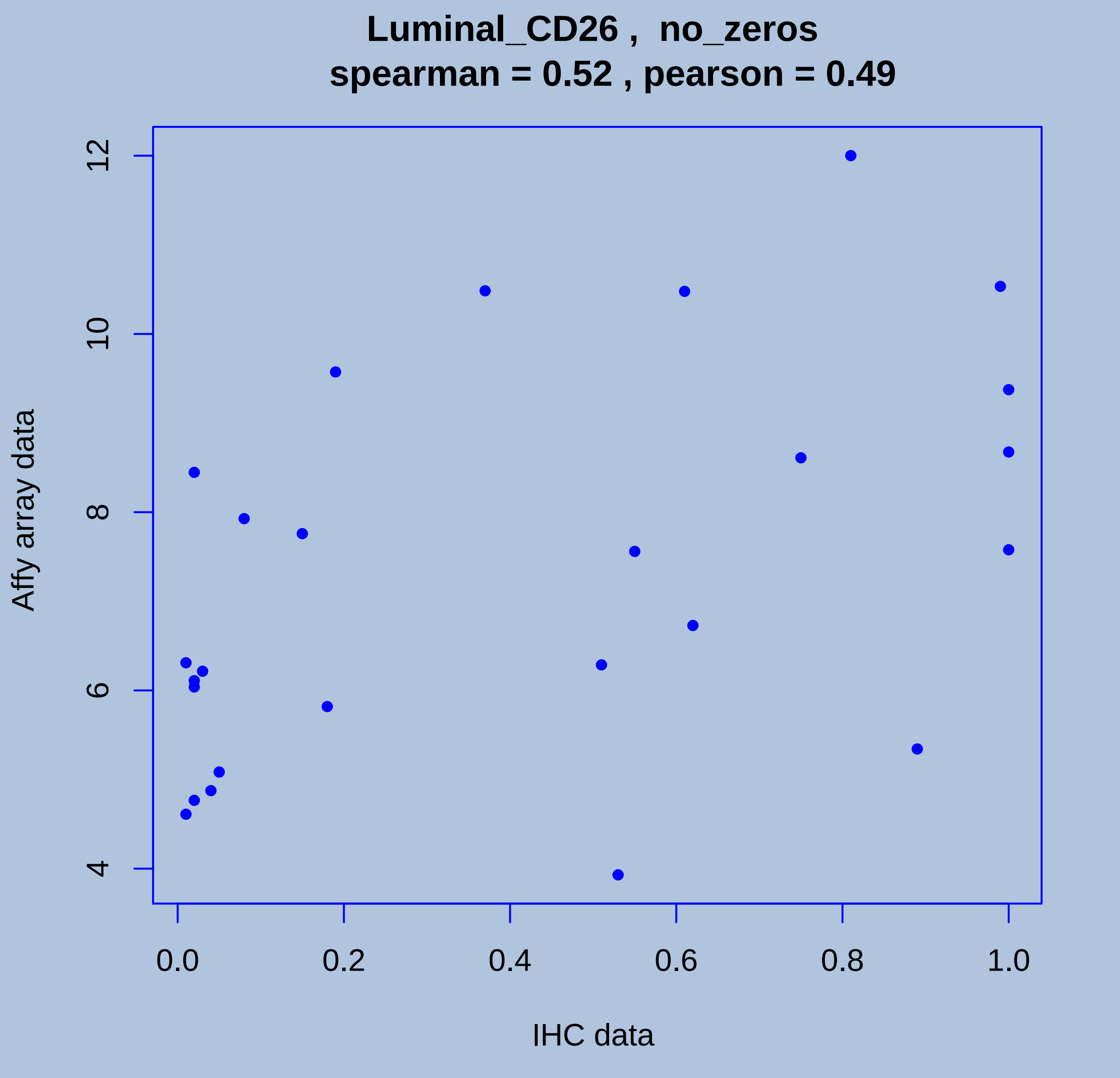
Luminal_CD26 , no_zeros
spearman = 0.52 , pearson = 0.49
Affy array data
IHC data
12
10
8
6
4
0.0
0.2
0.4
0.6
0.8
1.0

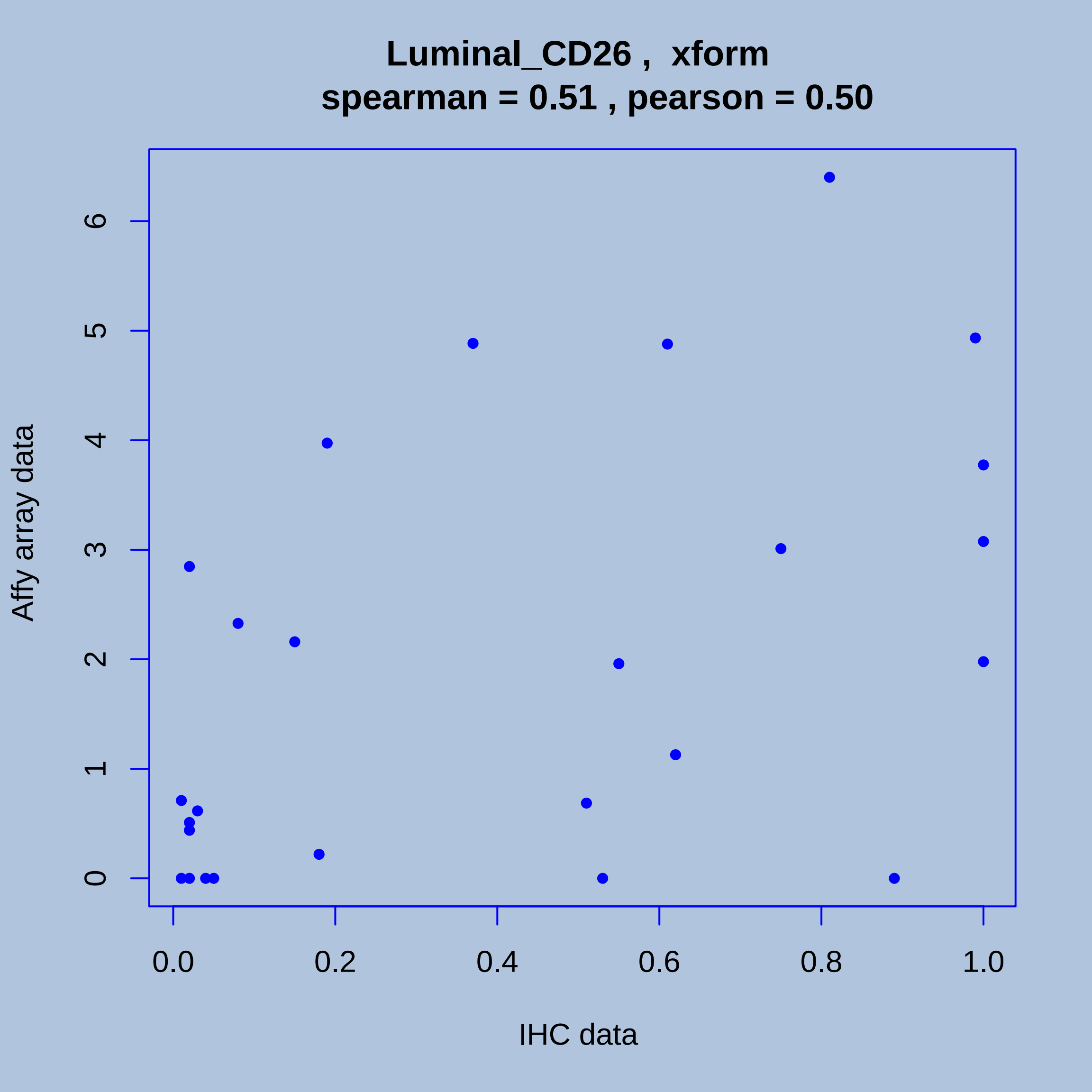
Luminal_CD26 , xform
spearman = 0.51 , pearson = 0.50
Affy array data
IHC data
0
1
2
3
4
5
6
0.0
0.2
0.4
0.6
0.8
1.0

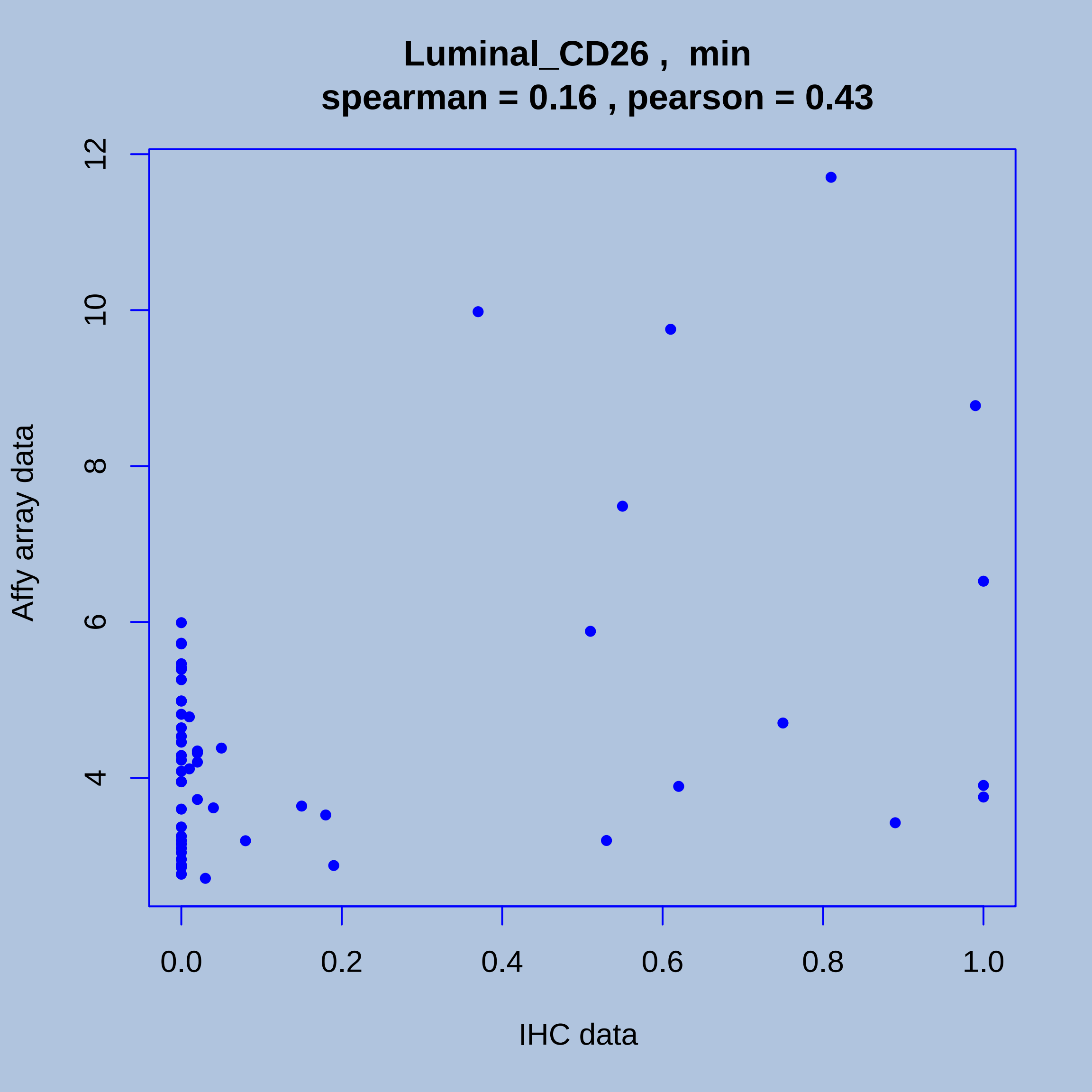

Luminal_CD26 , min
spearman = 0.16 , pearson = 0.43
Affy array data
IHC data
4
6
8
10
12
0.0
0.2
0.4
0.6
0.8
1.0

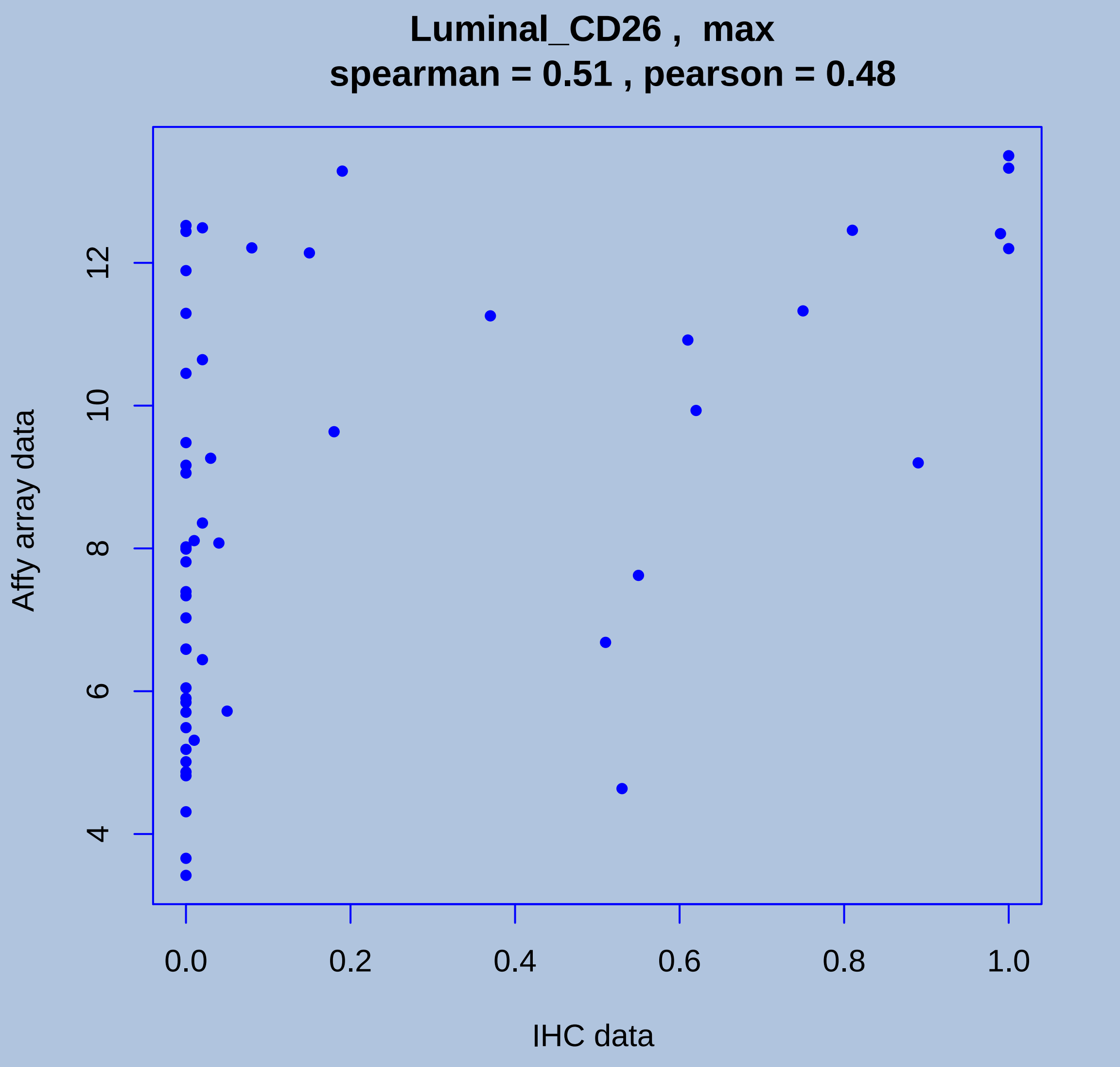
Luminal_CD26 , max
spearman = 0.51 , pearson = 0.48
Affy array data
4
6
8
10
12
0.0
0.2
0.4
0.6
0.8
1.0
IHC data

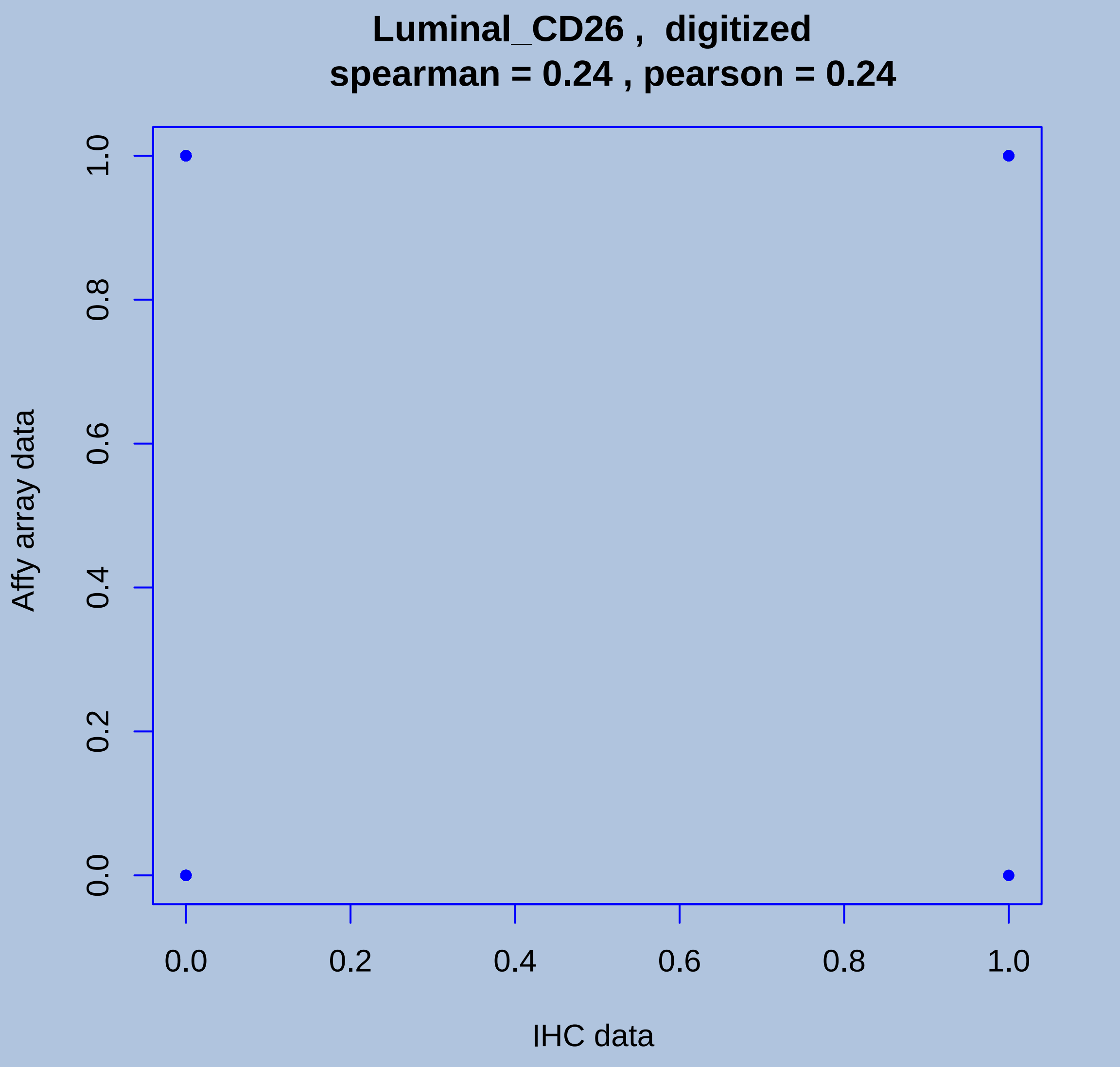
Luminal_CD26 , digitized
spearman = 0.24 , pearson = 0.24
Affy array data
0.0
0.2
0.4
0.6
0.8
1.0
IHC data

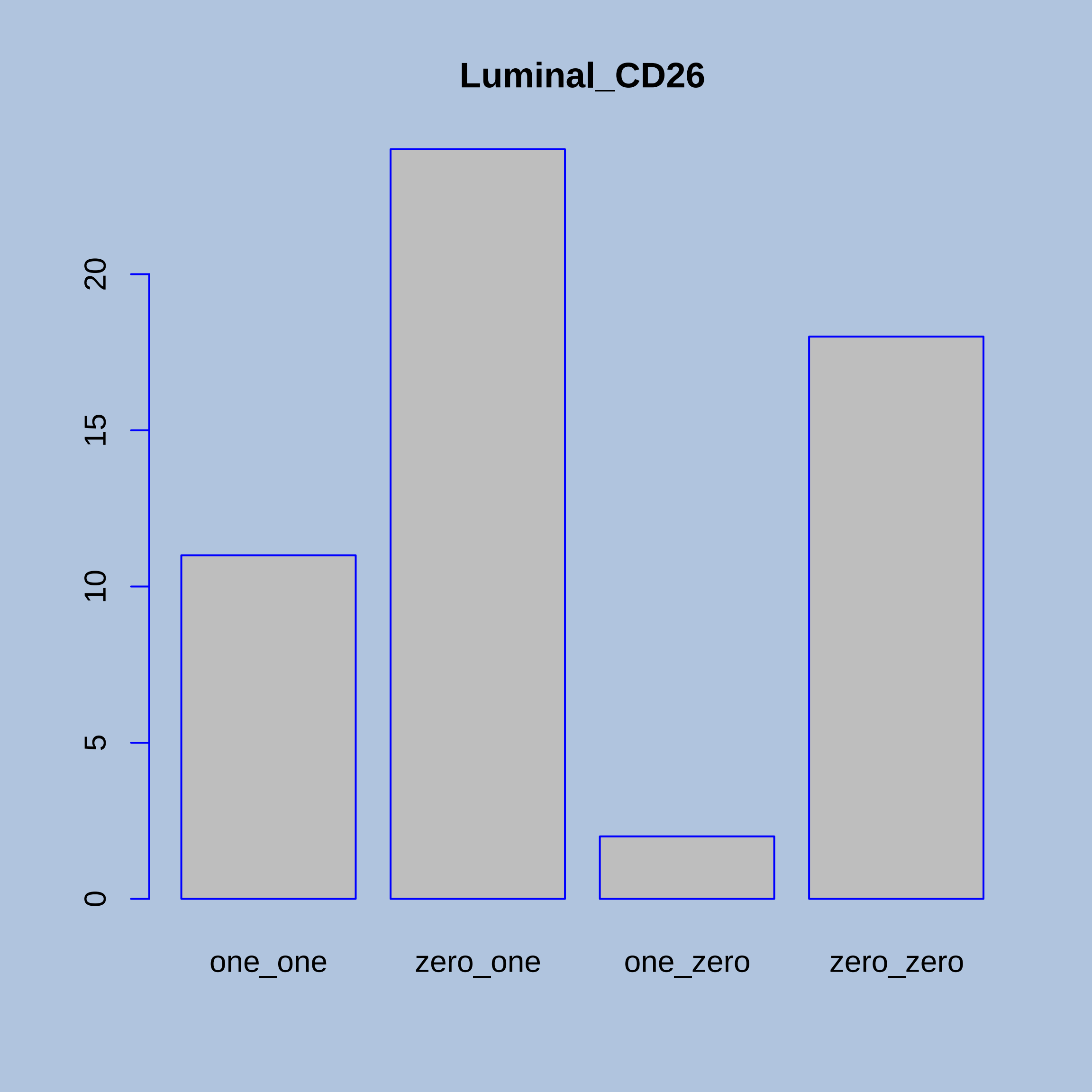
Luminal_CD26
0
5
10
15
20
one_one
zero_one
one_zero
zero_zero

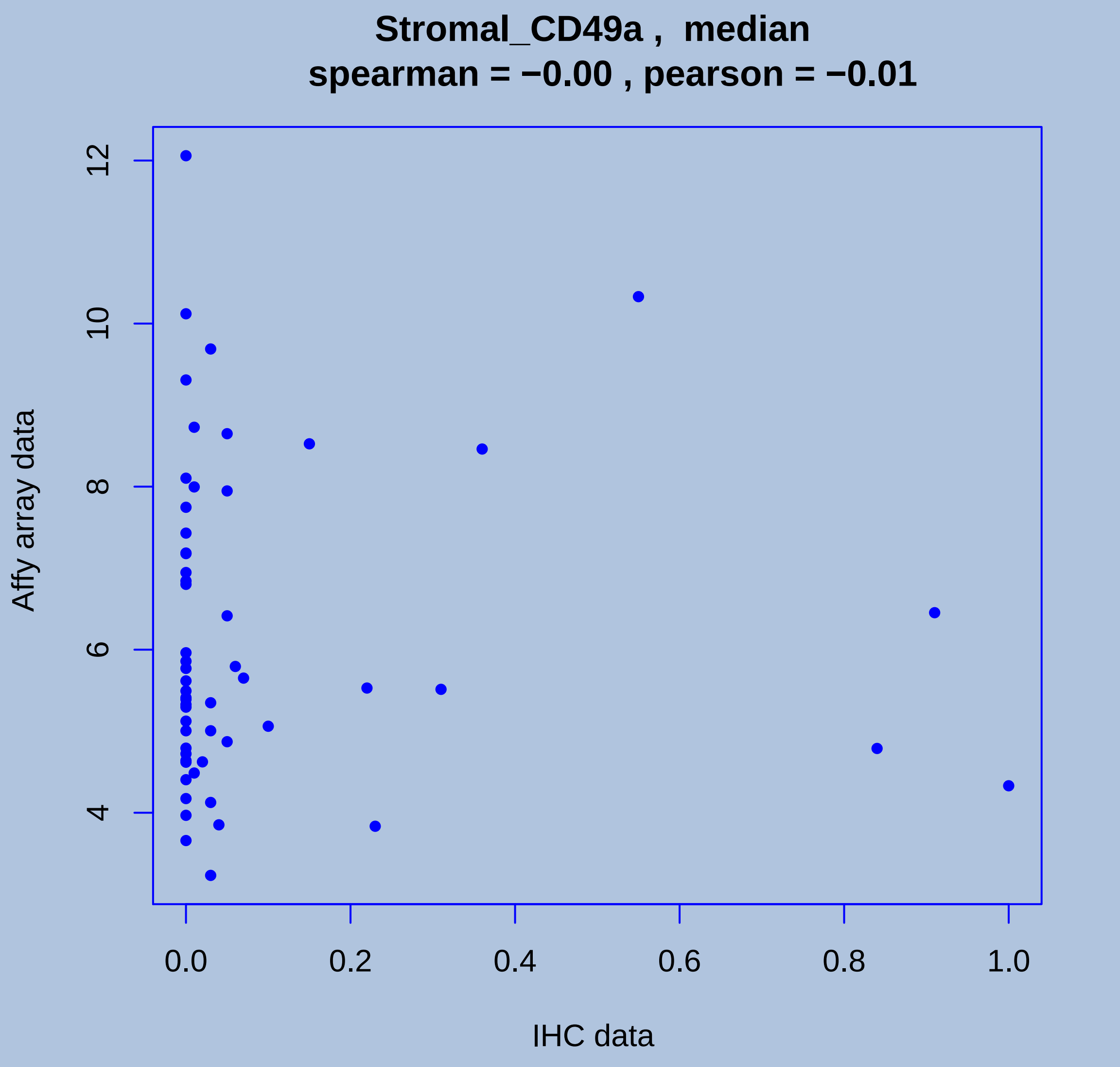
Stromal_CD49a , median
spearman = −0.00 , pearson = −0.01
Affy array data
IHC data
4
6
8
10
12
0.0
0.2
0.4
0.6
0.8
1.0

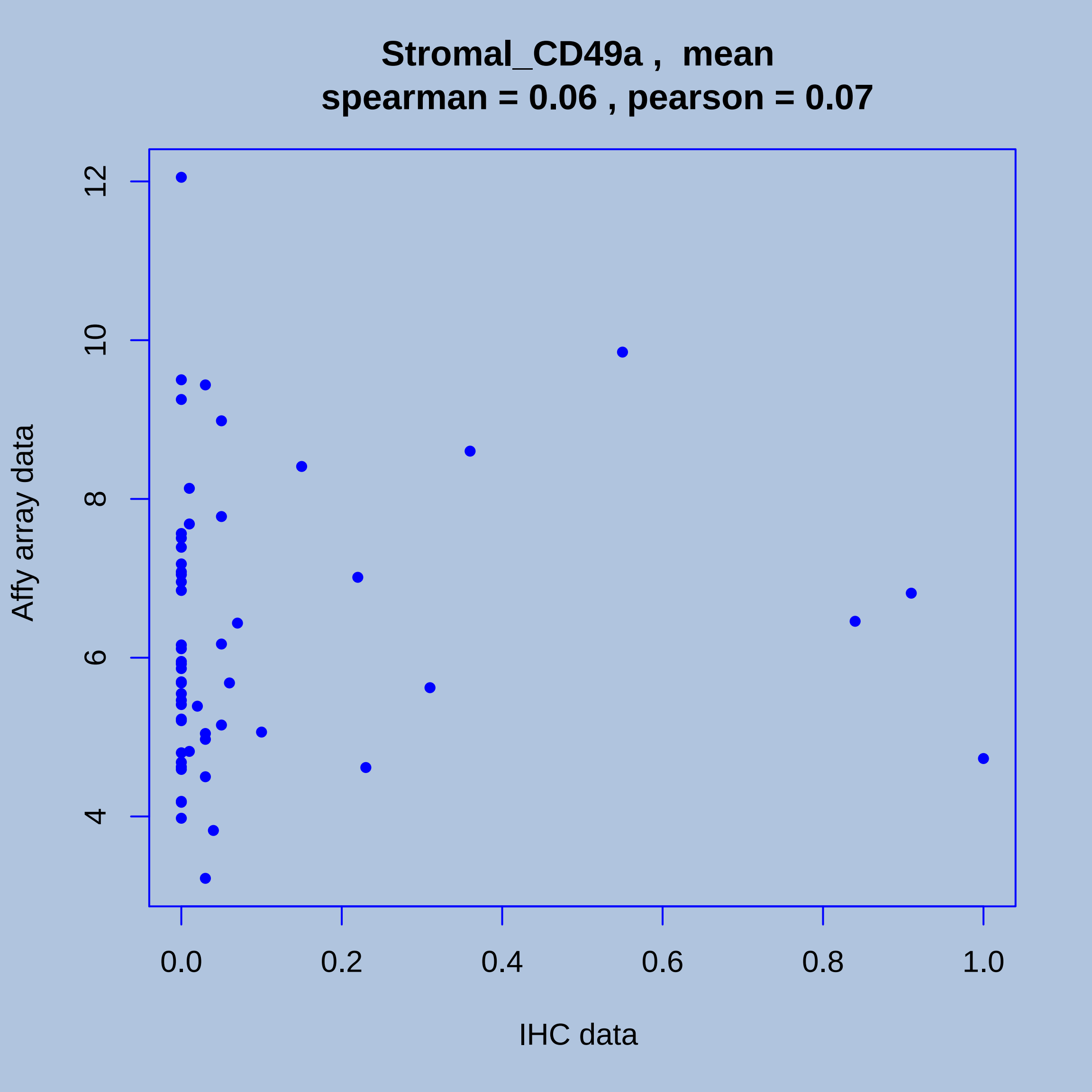
Stromal_CD49a , mean
spearman = 0.06 , pearson = 0.07
Affy array data
IHC data
4
6
8
10
12
0.0
0.2
0.4
0.6
0.8
1.0

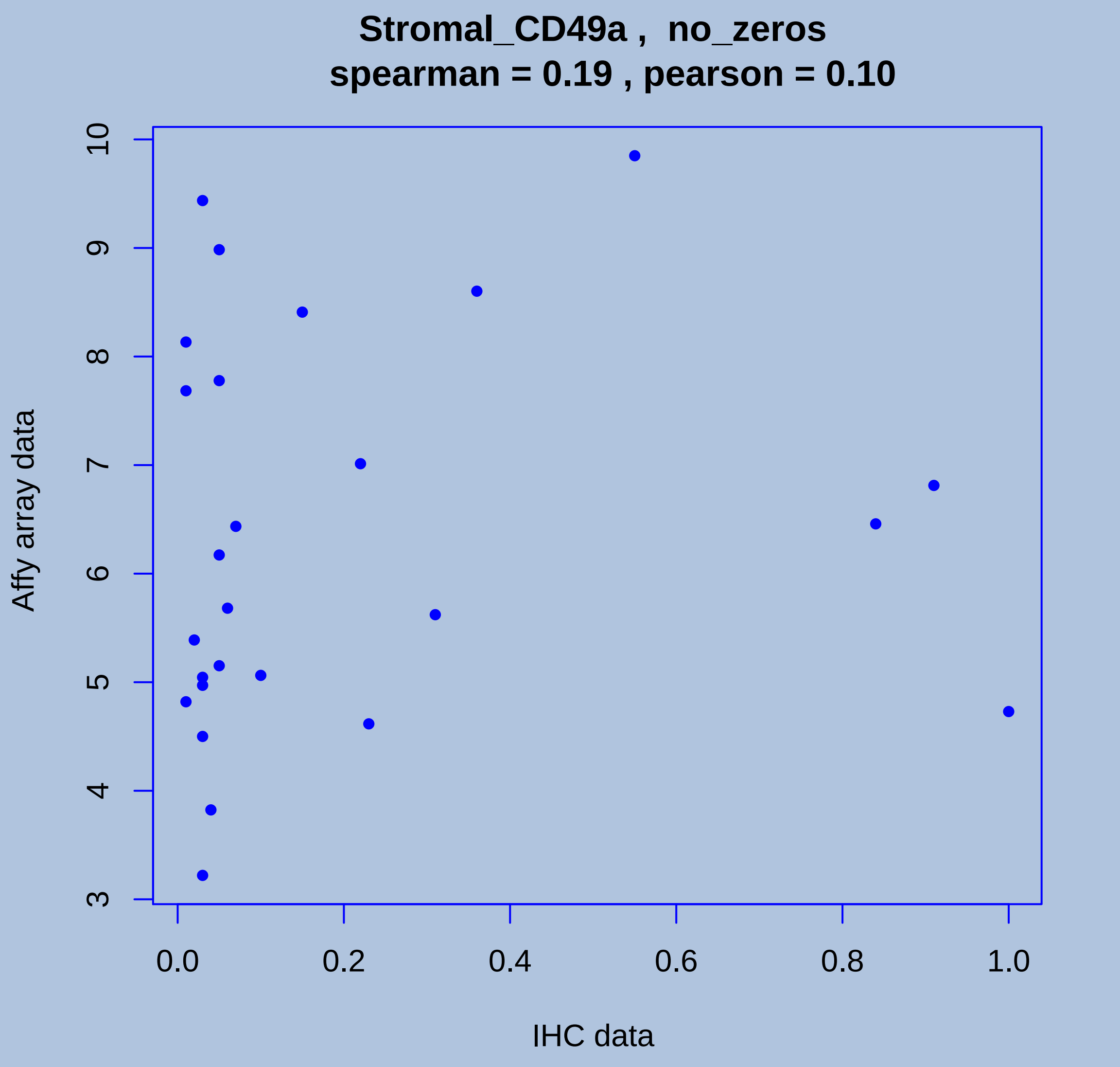

Stromal_CD49a , no_zeros
spearman = 0.19 , pearson = 0.10
Affy array data
IHC data
3
4
5
6
7
8
9
10
0.0
0.2
0.4
0.6
0.8
1.0

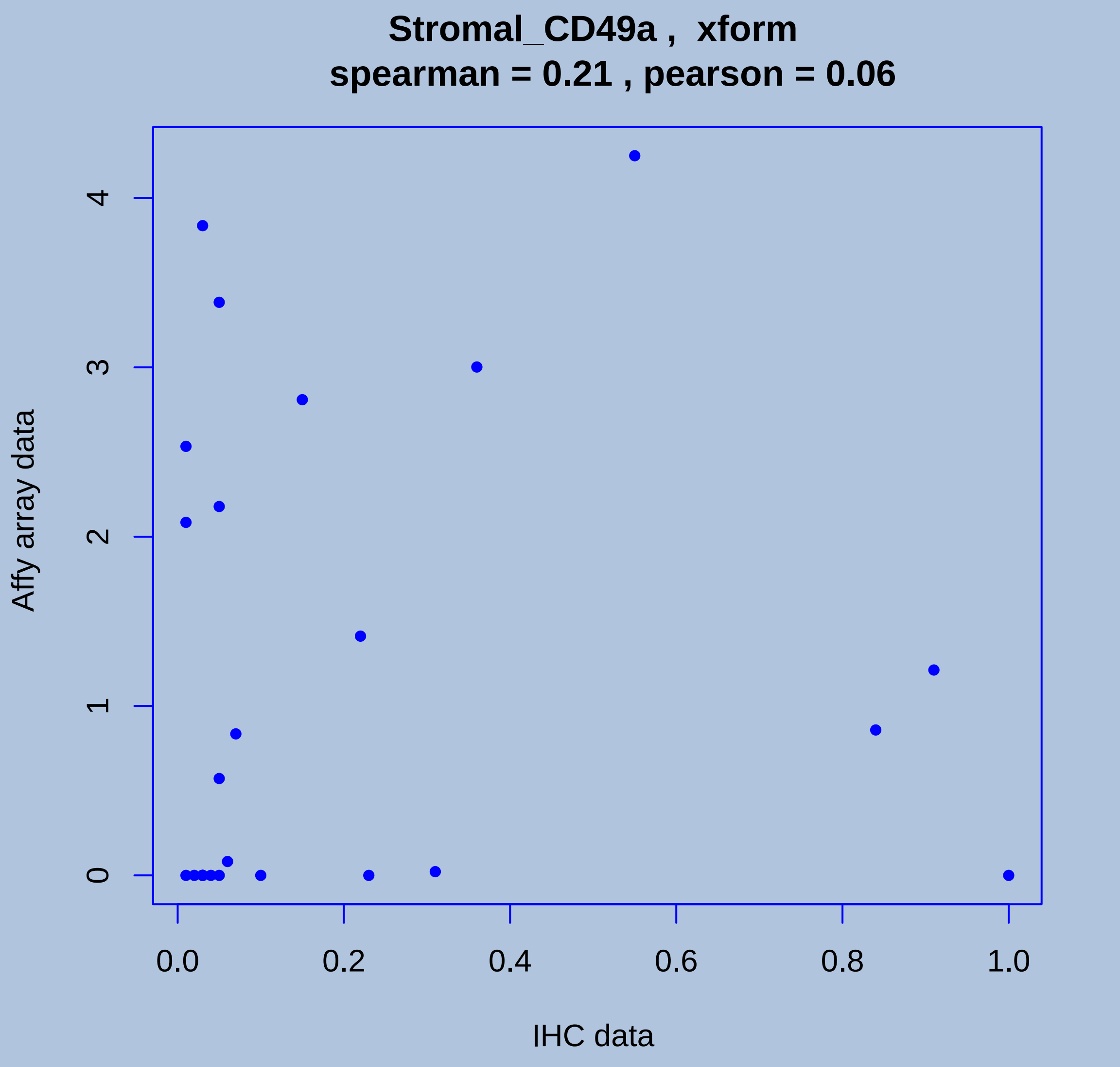
Stromal_CD49a , xform
spearman = 0.21 , pearson = 0.06
Affy array data
IHC data
0
1
2
3
4
0.0
0.2
0.4
0.6
0.8
1.0

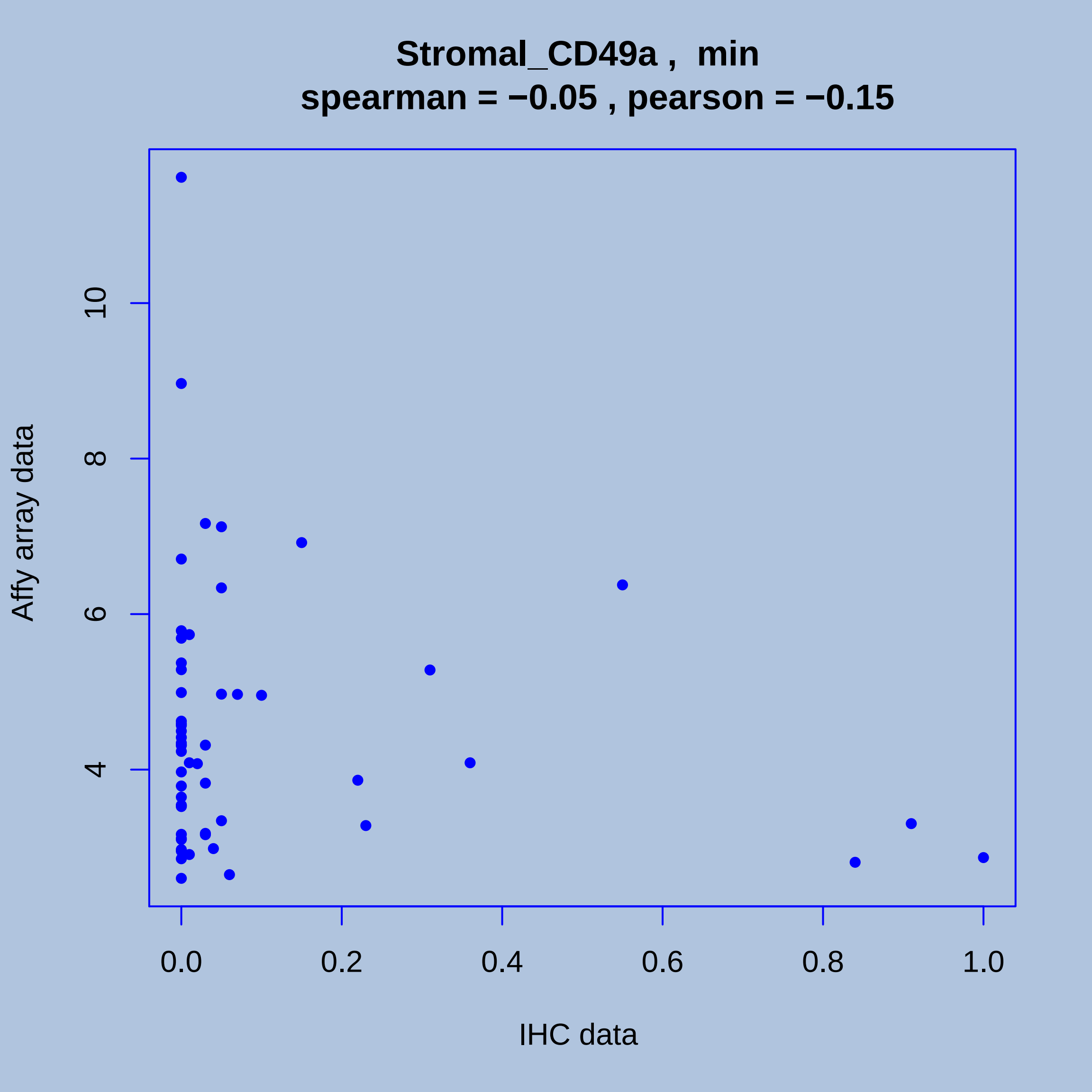
Stromal_CD49a , min
spearman = −0.05 , pearson = −0.15
Affy array data
IHC data
4
6
8
10
0.0
0.2
0.4
0.6
0.8
1.0

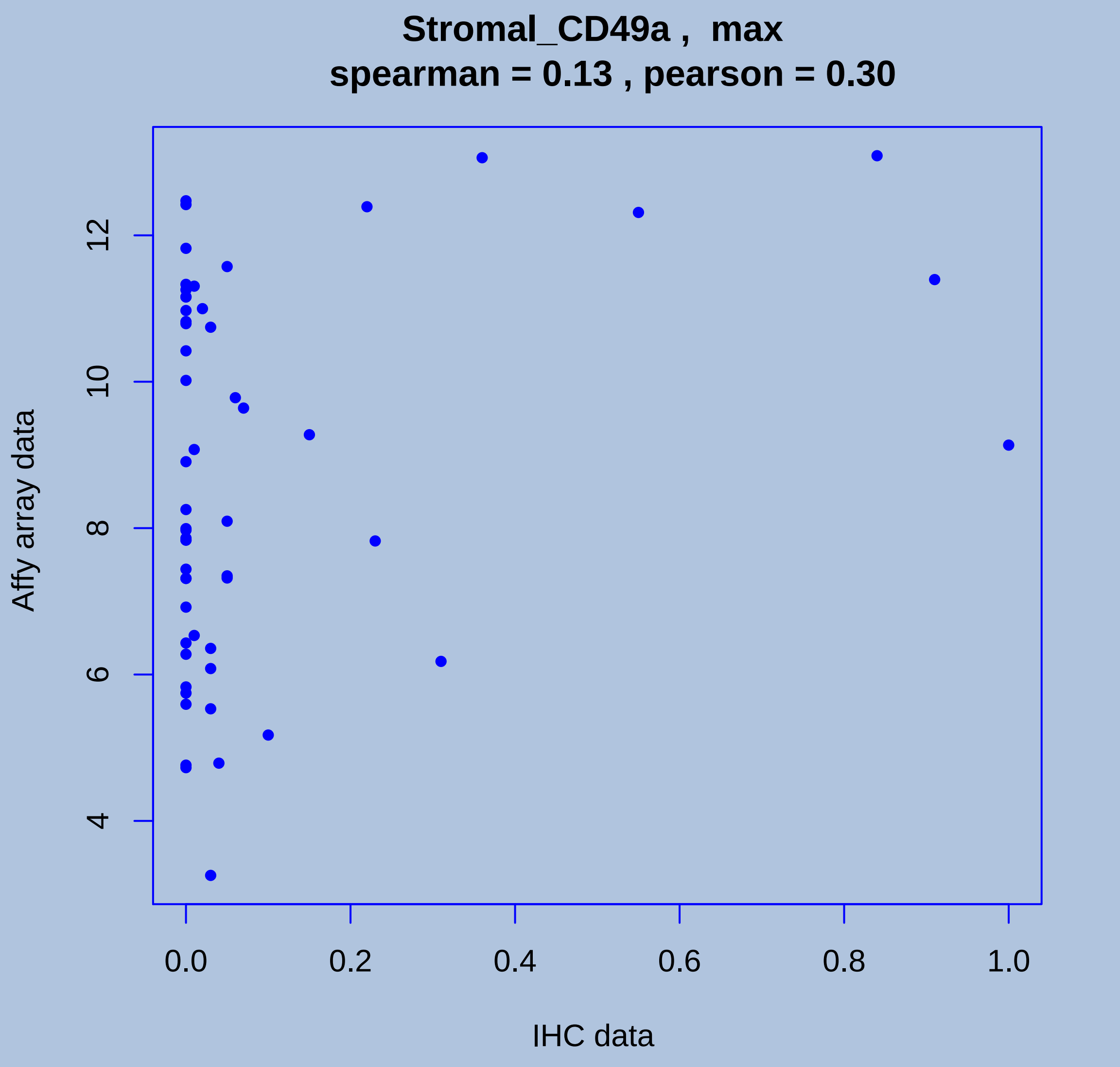

Stromal_CD49a , max
spearman = 0.13 , pearson = 0.30
Affy array data
4
6
8
10
12
0.0
0.2
0.4
0.6
0.8
1.0
IHC data

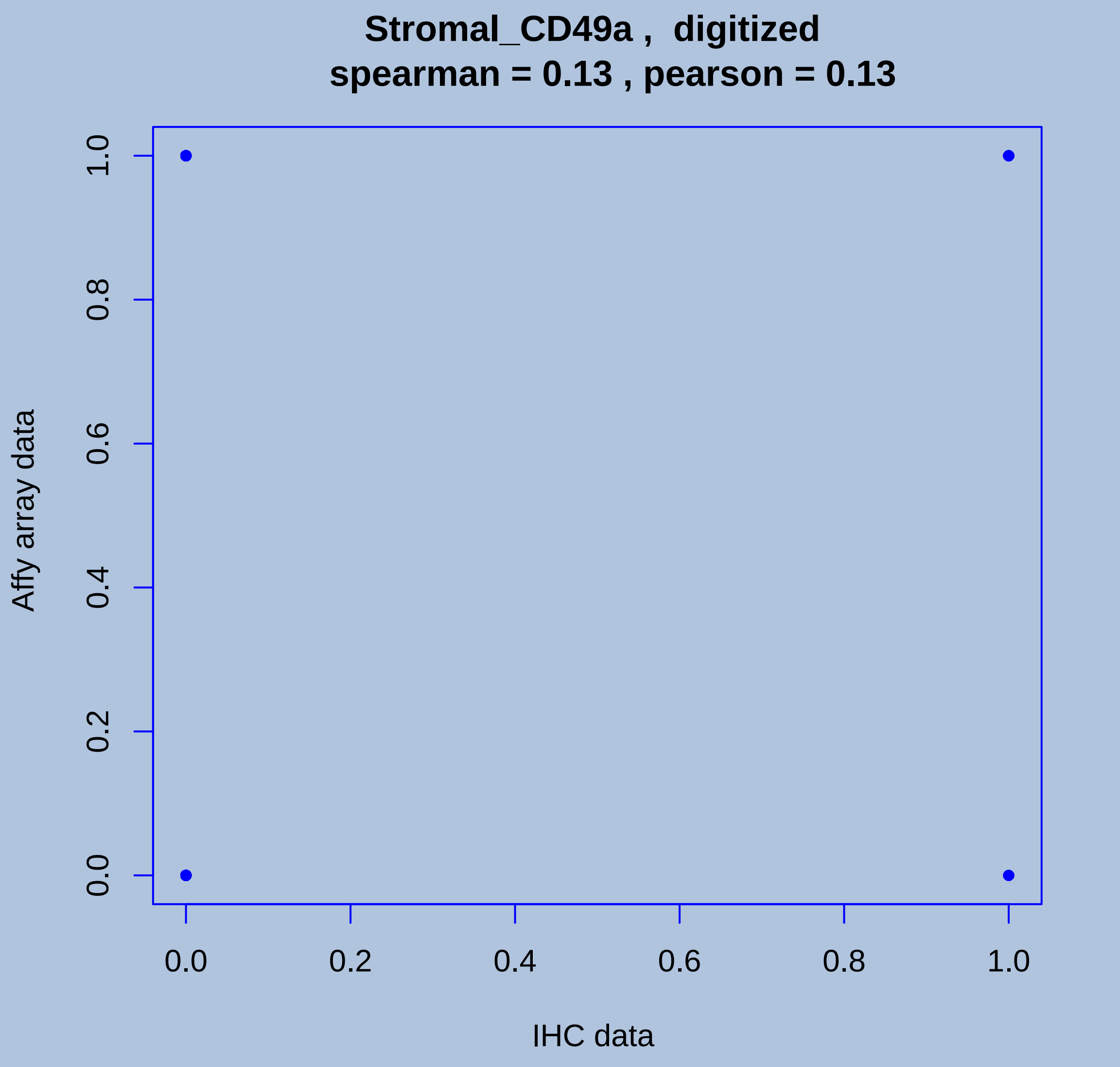
Stromal_CD49a , digitized
spearman = 0.13 , pearson = 0.13
Affy array data
IHC data
0.0
0.2
0.4
0.6
0.8
1.0

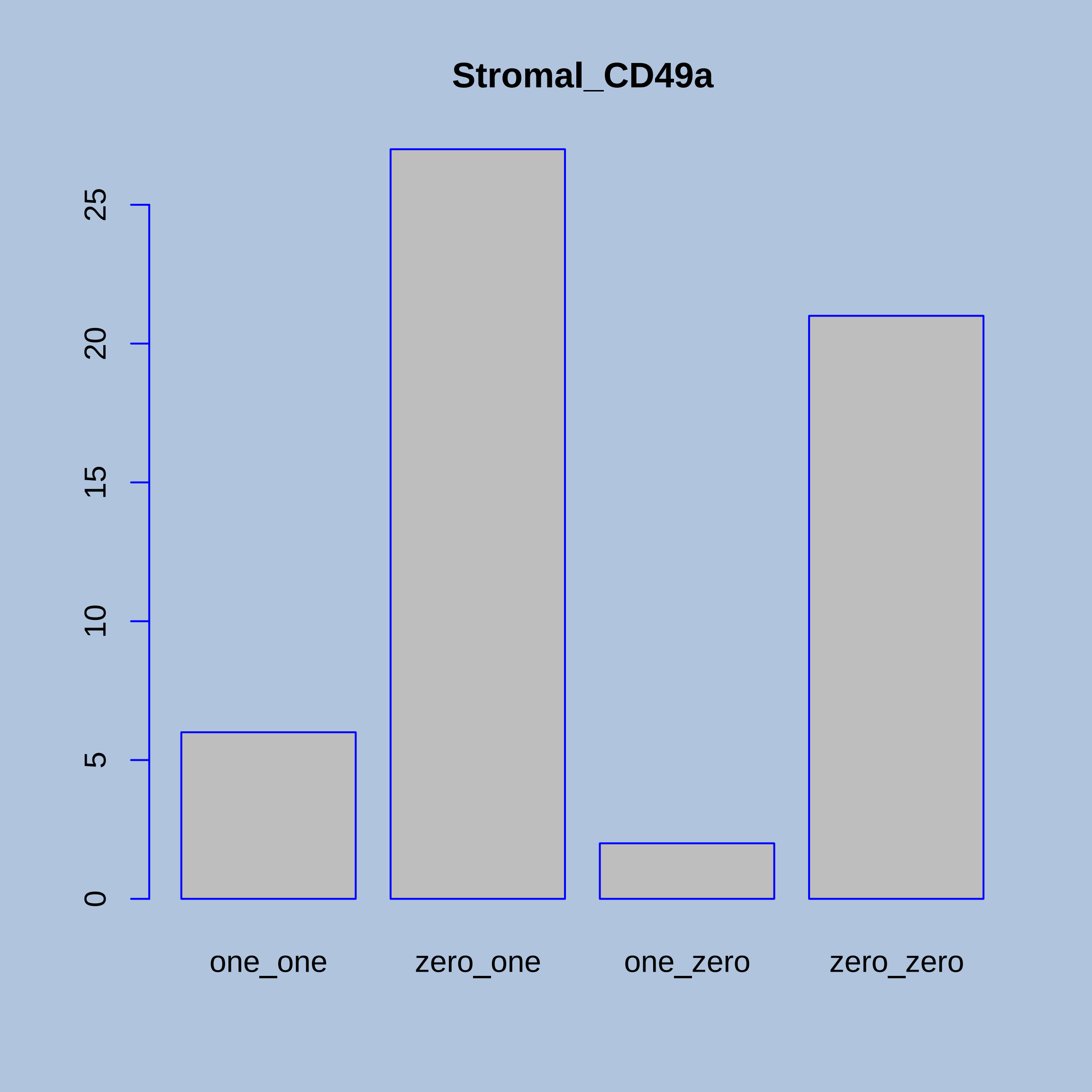
Stromal_CD49a
0
5
10
15
20
25
one_one
zero_one
one_zero
zero_zero

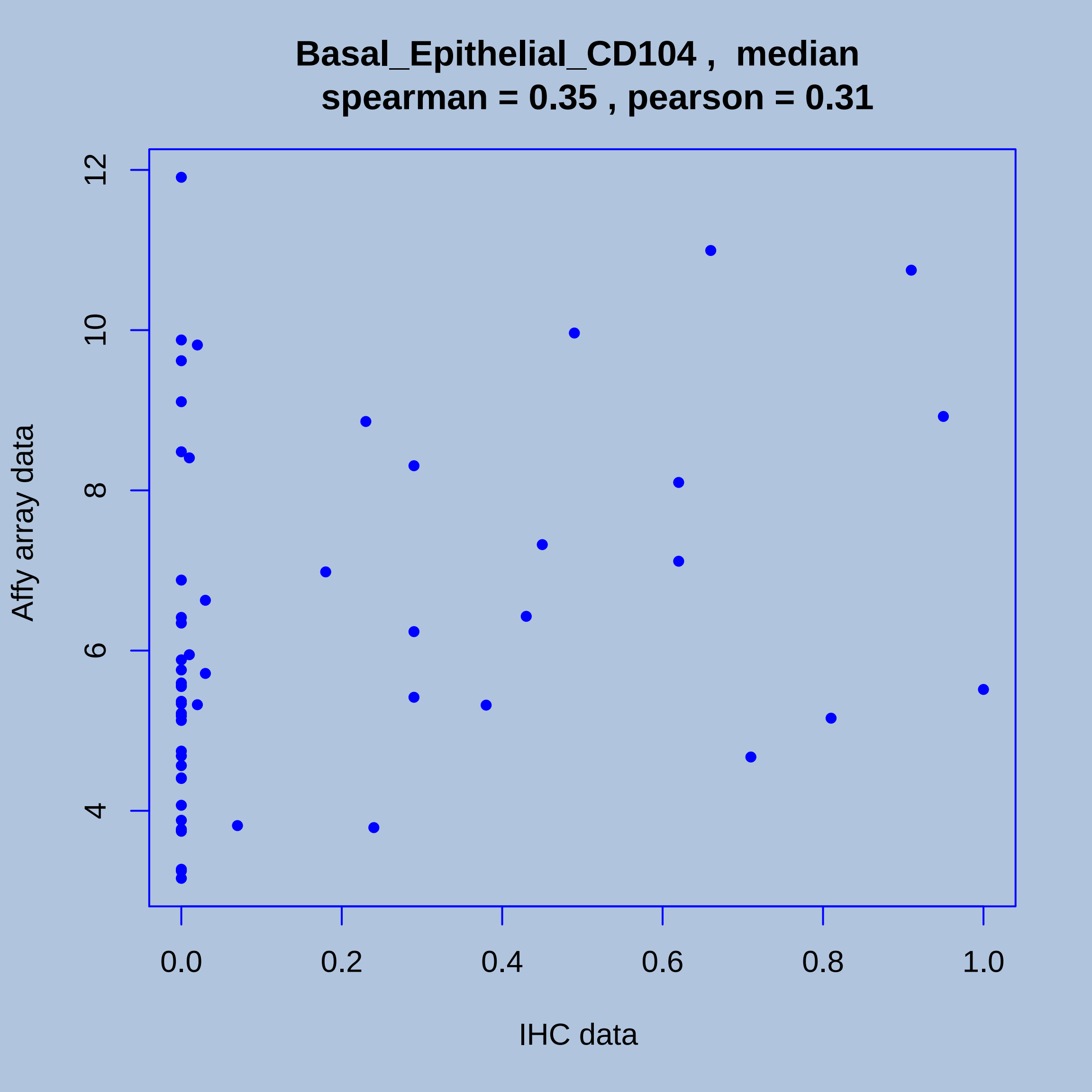
Basal_Epithelial_CD104 , median
spearman = 0.35 , pearson = 0.31
Affy array data
IHC data
4
6
8
10
12
0.0
0.2
0.4
0.6
0.8
1.0

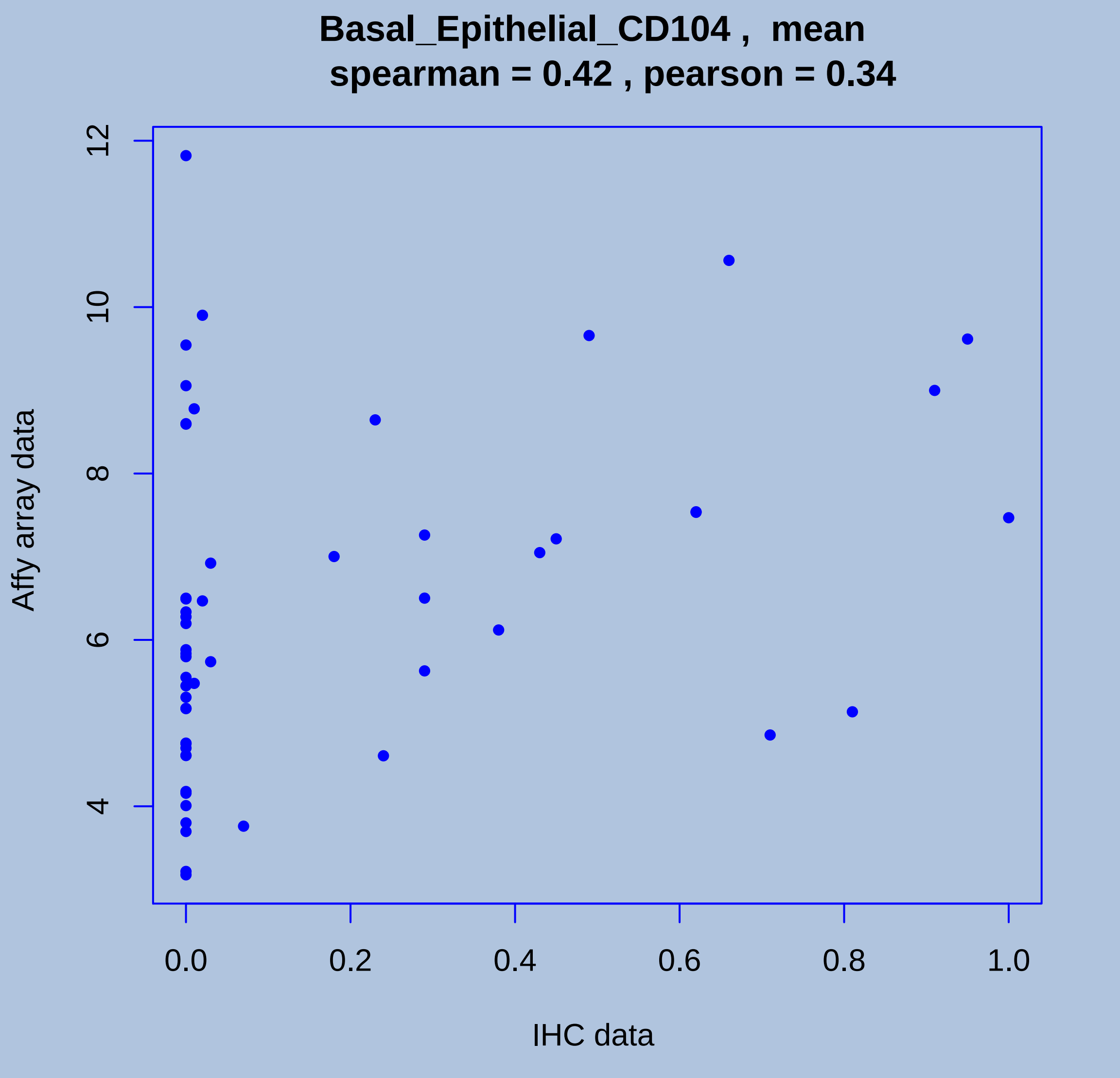

Basal_Epithelial_CD104 , mean
spearman = 0.42 , pearson = 0.34
Affy array data
IHC data
4
6
8
10
12
0.0
0.2
0.4
0.6
0.8
1.0

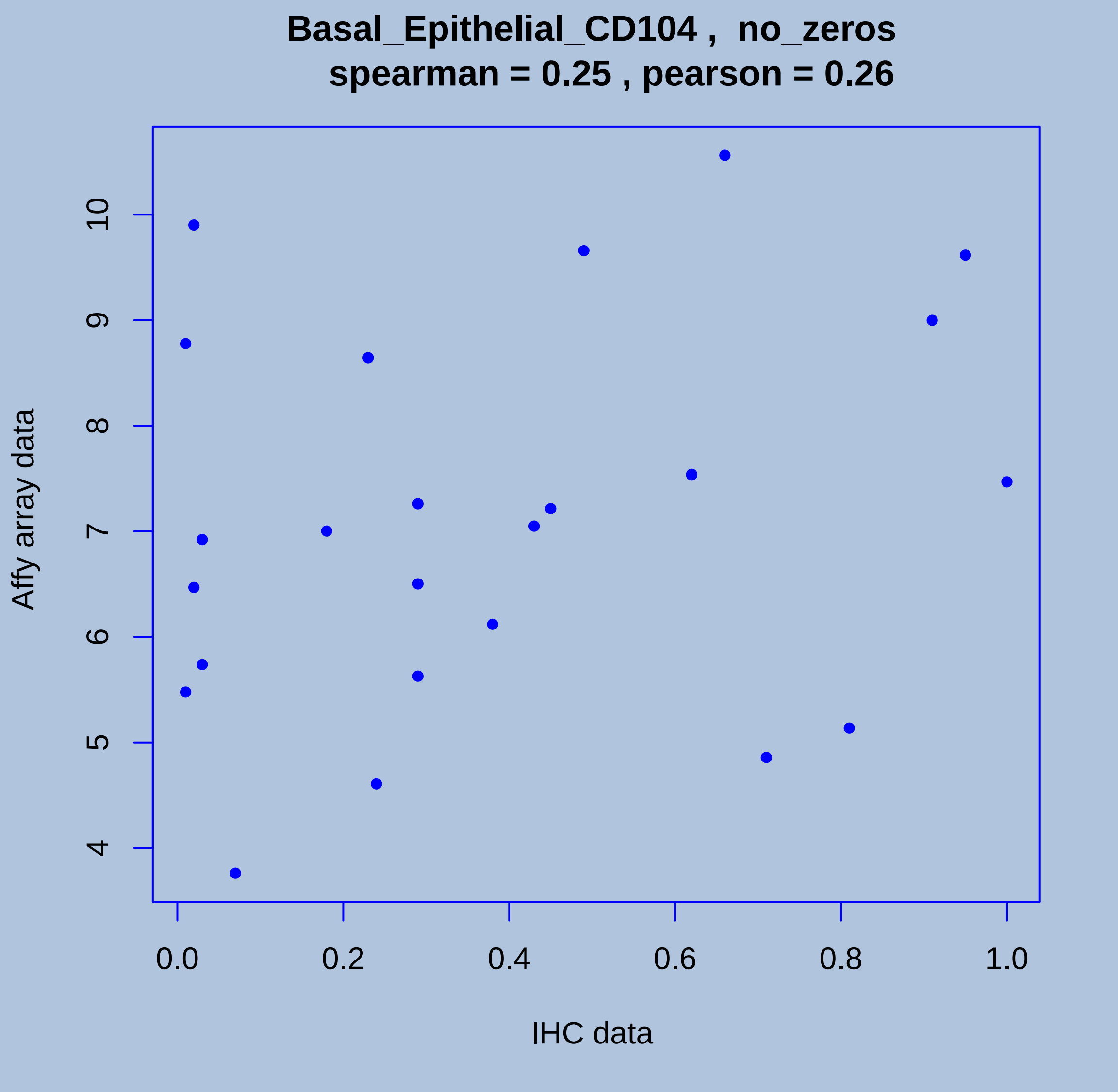
Basal_Epithelial_CD104 , no_zeros
spearman = 0.25 , pearson = 0.26
Affy array data
IHC data
4
5
6
7
8
9
10
0.0
0.2
0.4
0.6
0.8
1.0

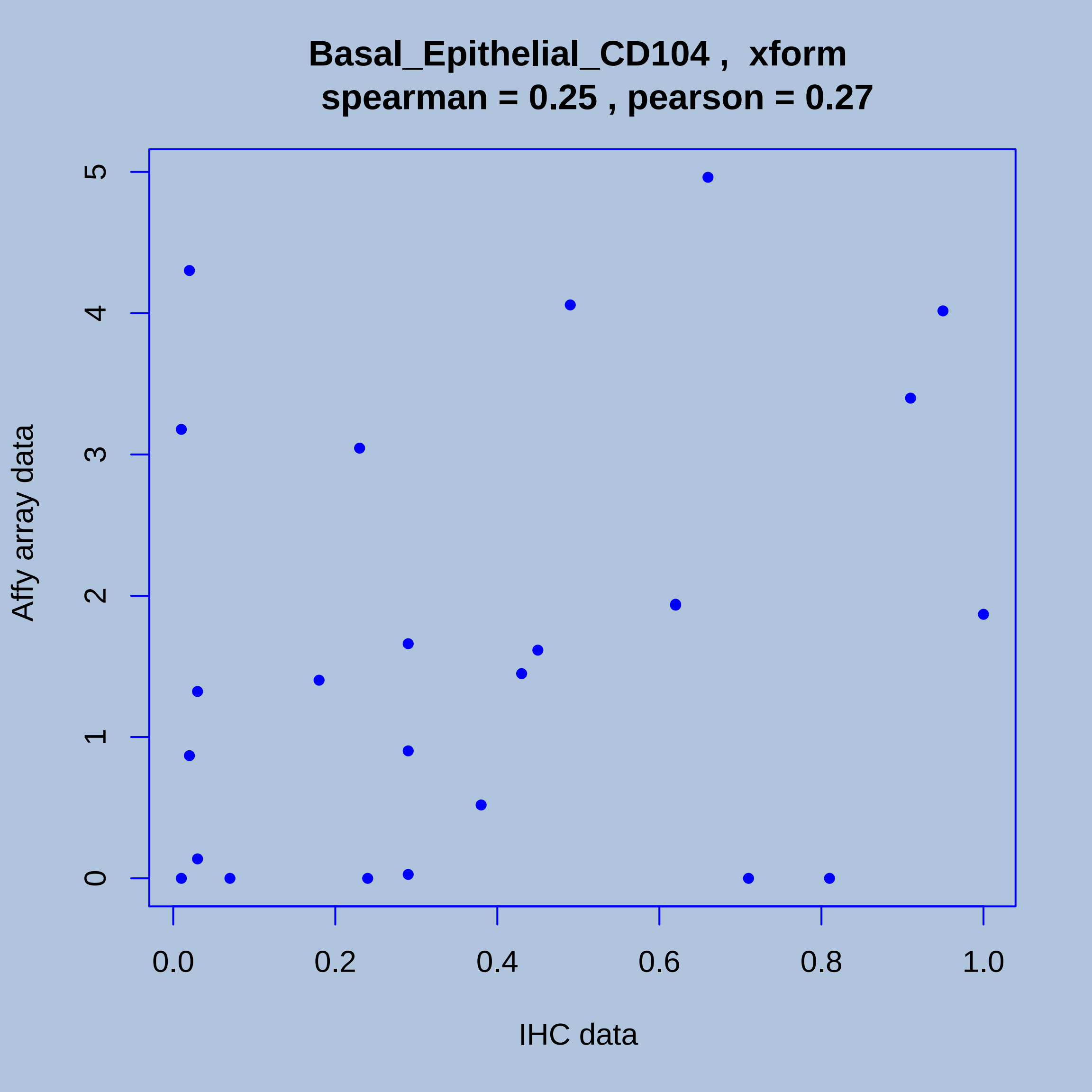
Basal_Epithelial_CD104 , xform
spearman = 0.25 , pearson = 0.27
Affy array data
0
1
2
3
4
5
0.0
0.2
0.4
0.6
0.8
1.0
IHC data

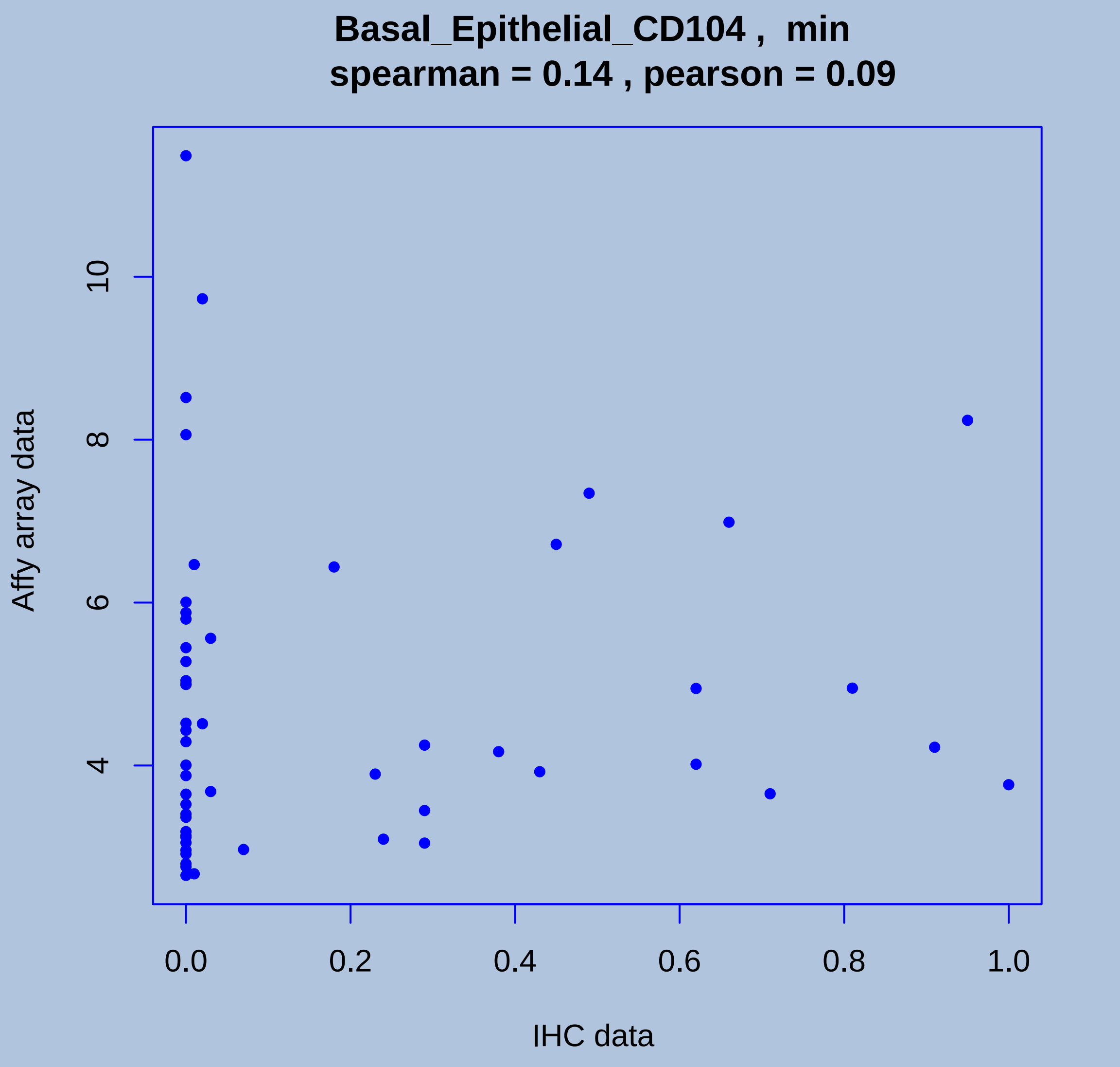
Basal_Epithelial_CD104 , min
spearman = 0.14 , pearson = 0.09
Affy array data
IHC data
4
6
8
10
0.0
0.2
0.4
0.6
0.8
1.0

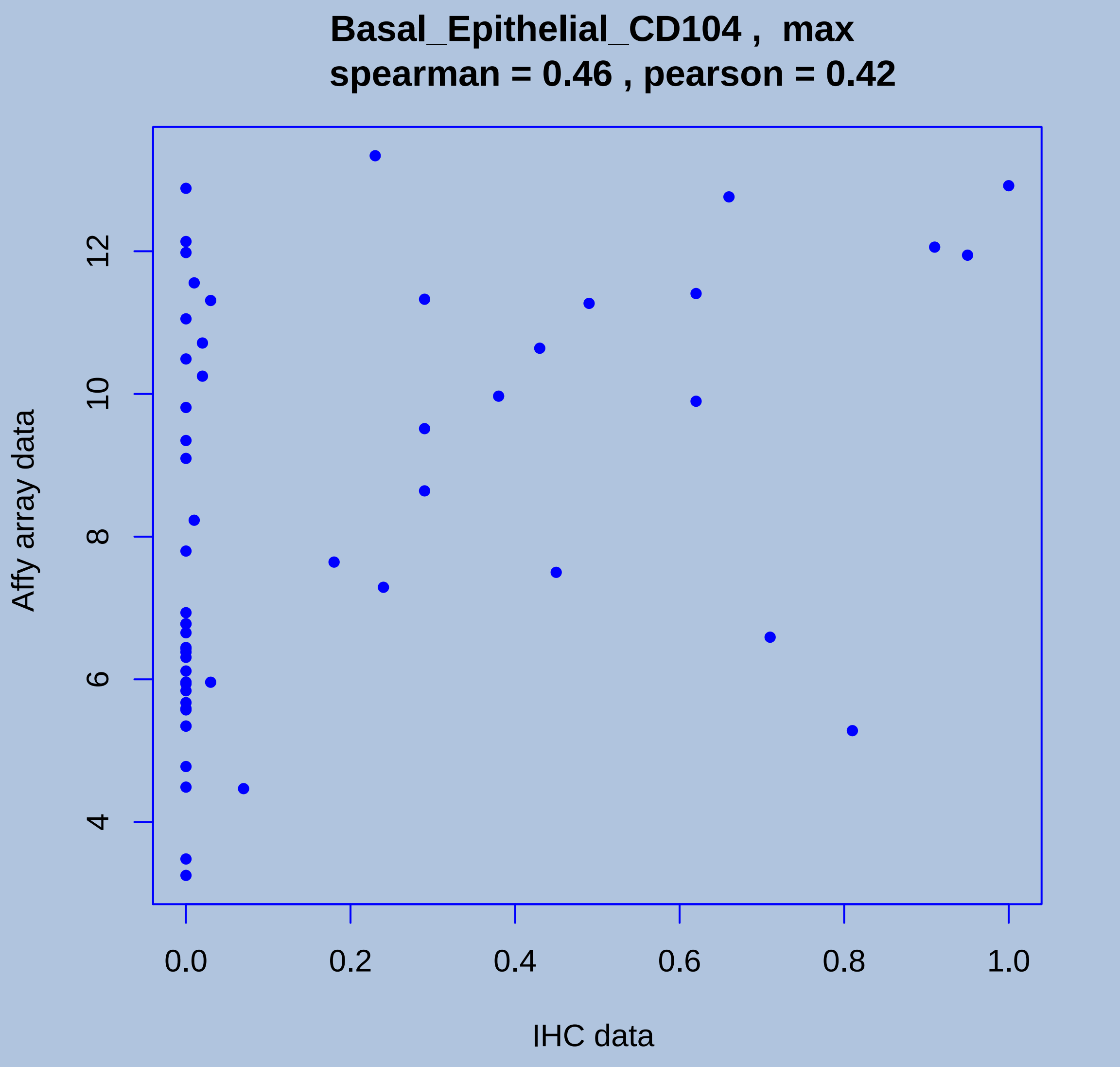

Basal_Epithelial_CD104 , max
spearman = 0.46 , pearson = 0.42
Affy array data
IHC data
4
6
8
10
12
0.0
0.2
0.4
0.6
0.8
1.0

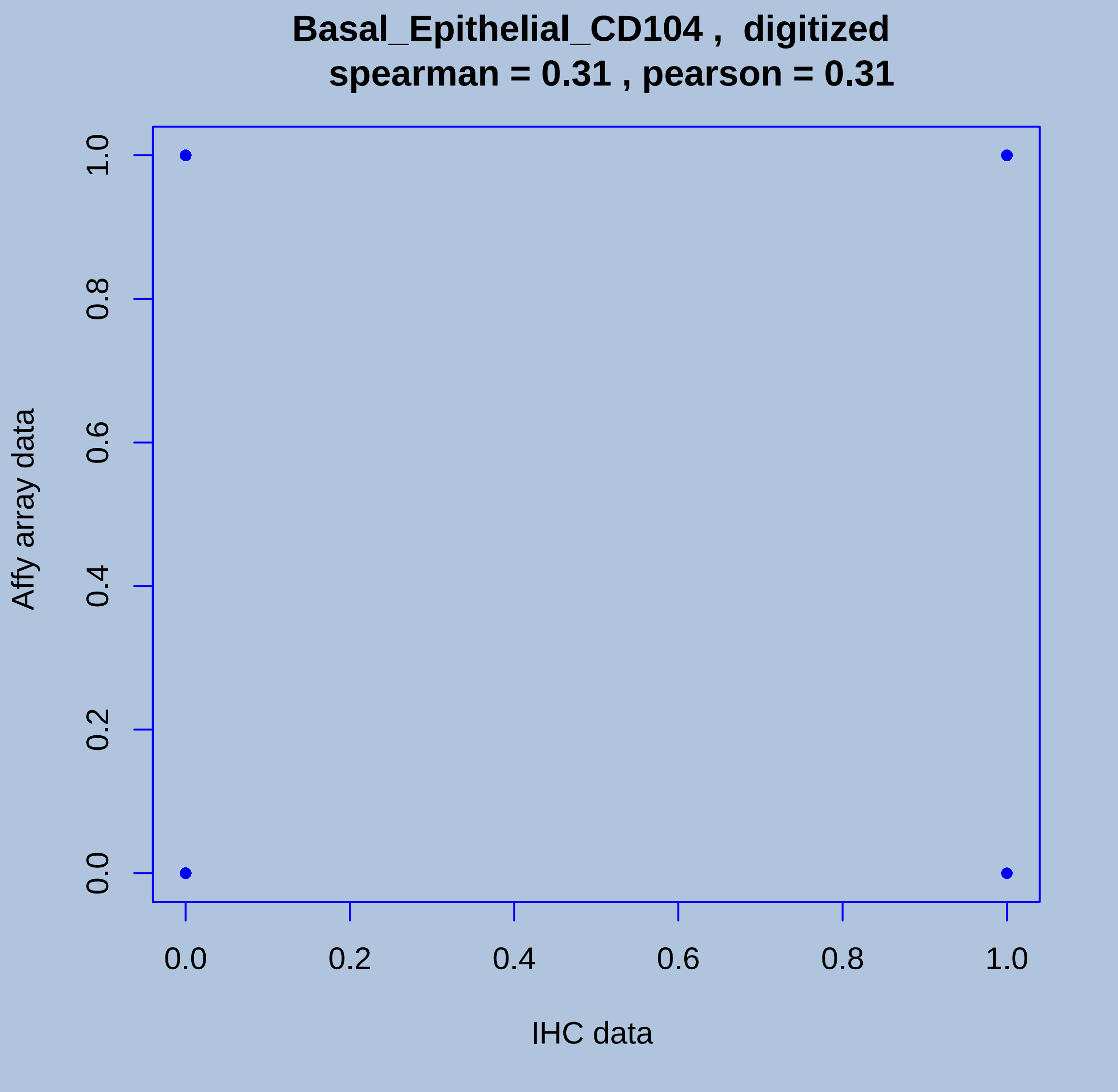
Basal_Epithelial_CD104 , digitized
spearman = 0.31 , pearson = 0.31
Affy array data
0.0
0.2
0.4
0.6
0.8
1.0
IHC data
0.0
0.2
0.4
0.6
0.8
1.0

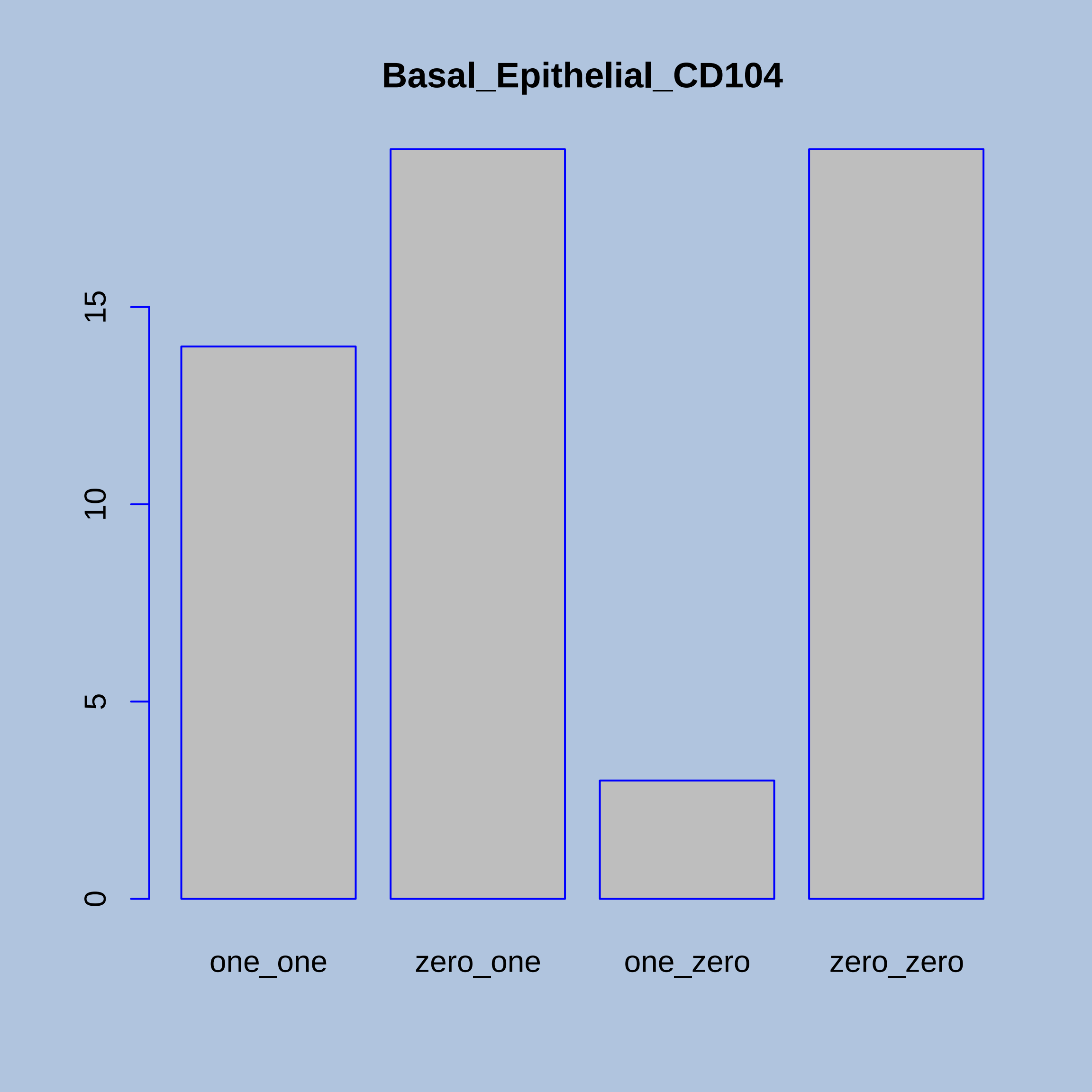
Basal_Epithelial_CD104
0
5
10
15
one_one
zero_one
one_zero
zero_zero

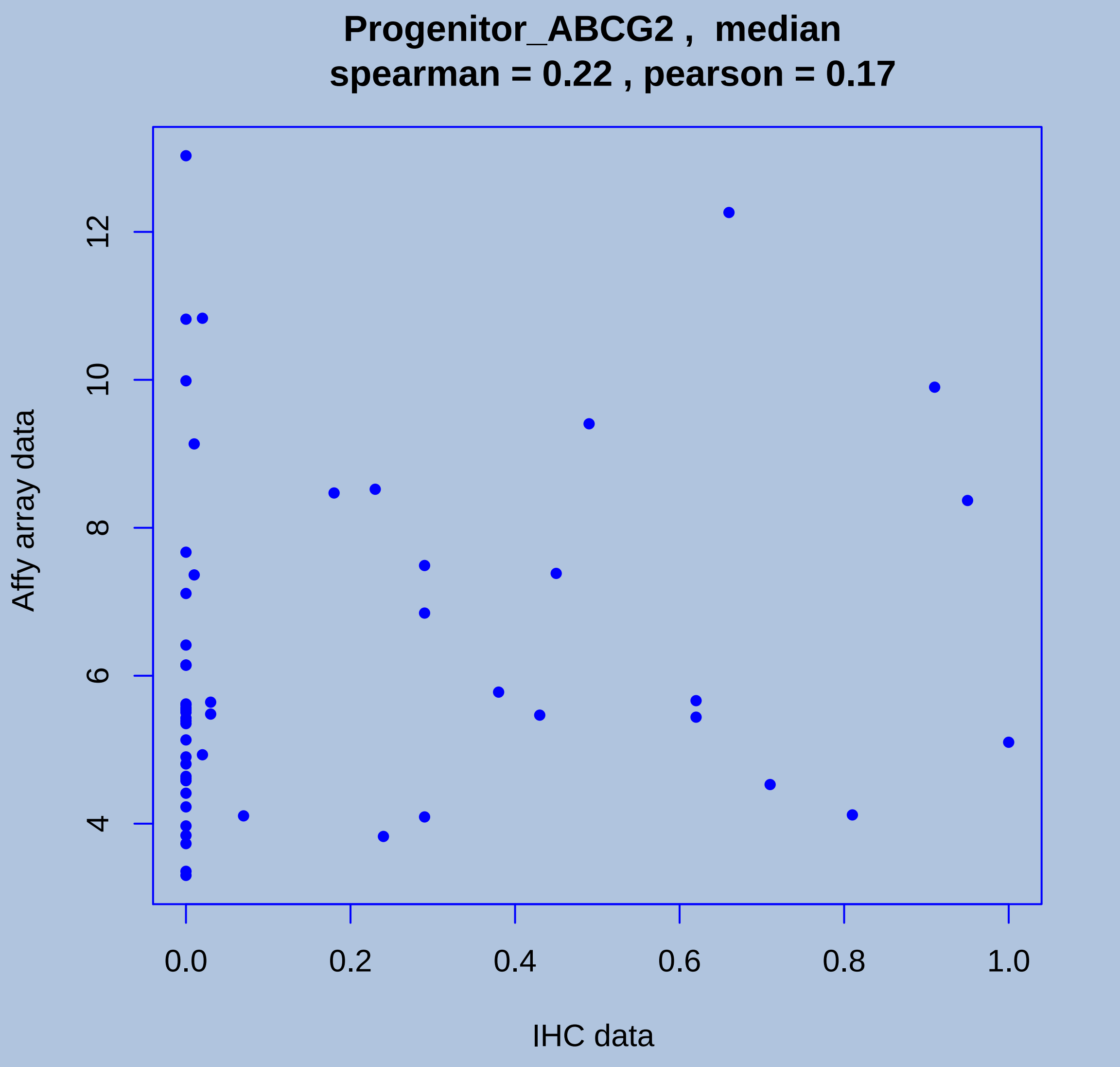
Progenitor_ABCG2 , median
spearman = 0.22 , pearson = 0.17
Affy array data
4
6
8
10
12
0.0
0.2
0.4
0.6
0.8
1.0
IHC data

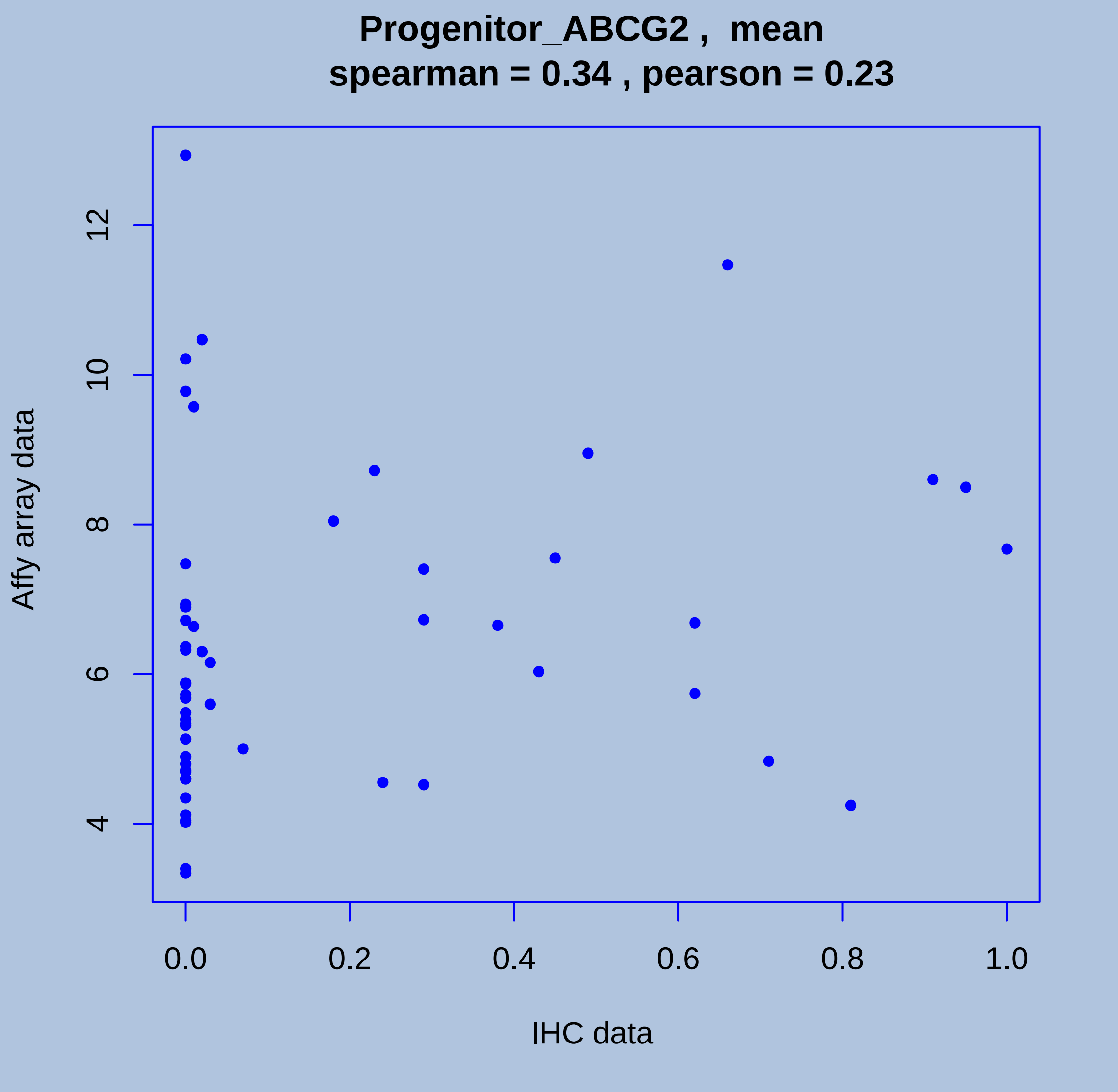

Progenitor_ABCG2 , mean
spearman = 0.34 , pearson = 0.23
Affy array data
IHC data
4
6
8
10
12
0.0
0.2
0.4
0.6
0.8
1.0

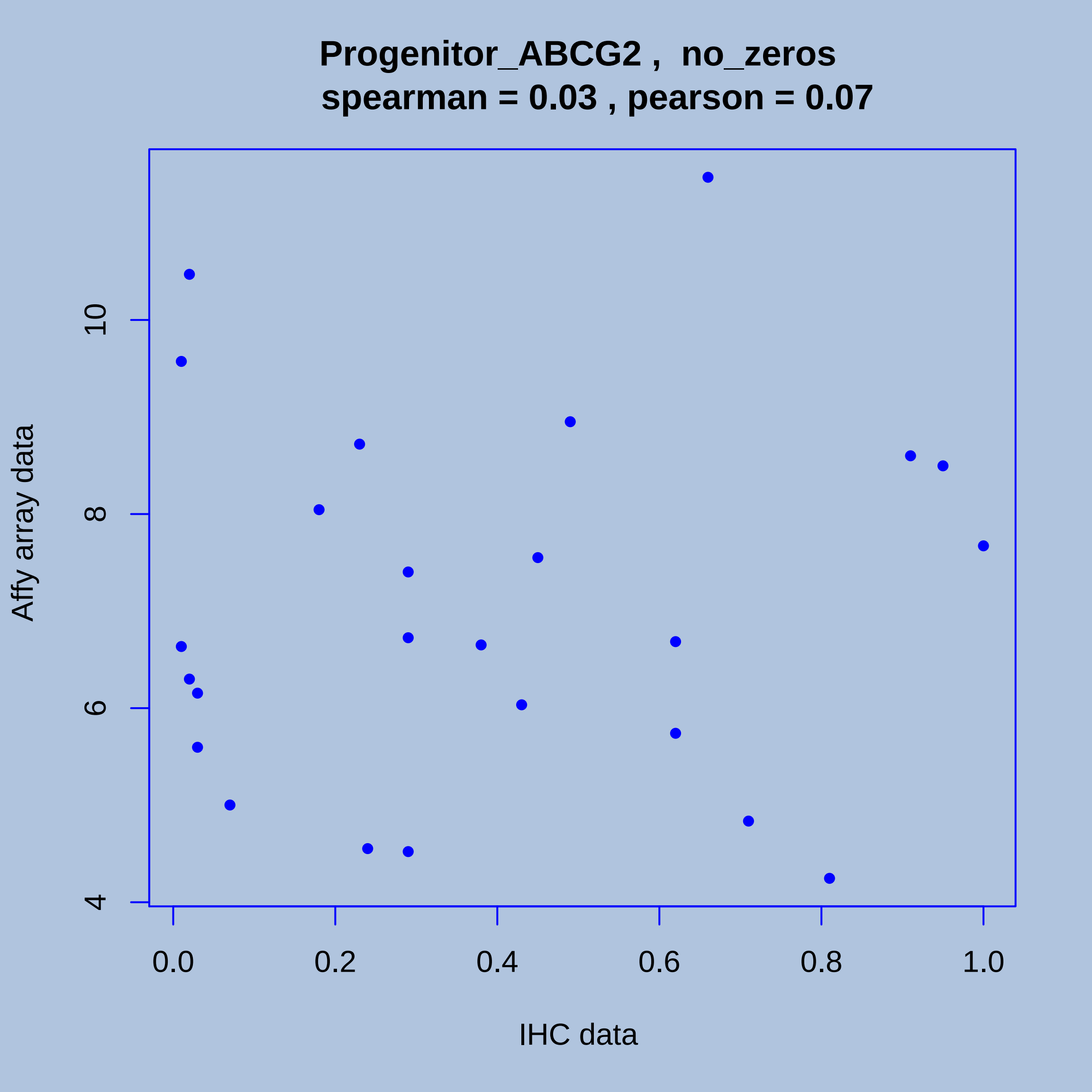
Progenitor_ABCG2 , no_zeros
spearman = 0.03 , pearson = 0.07
Affy array data
4
6
8
10
0.0
0.2
0.4
0.6
0.8
1.0
IHC data

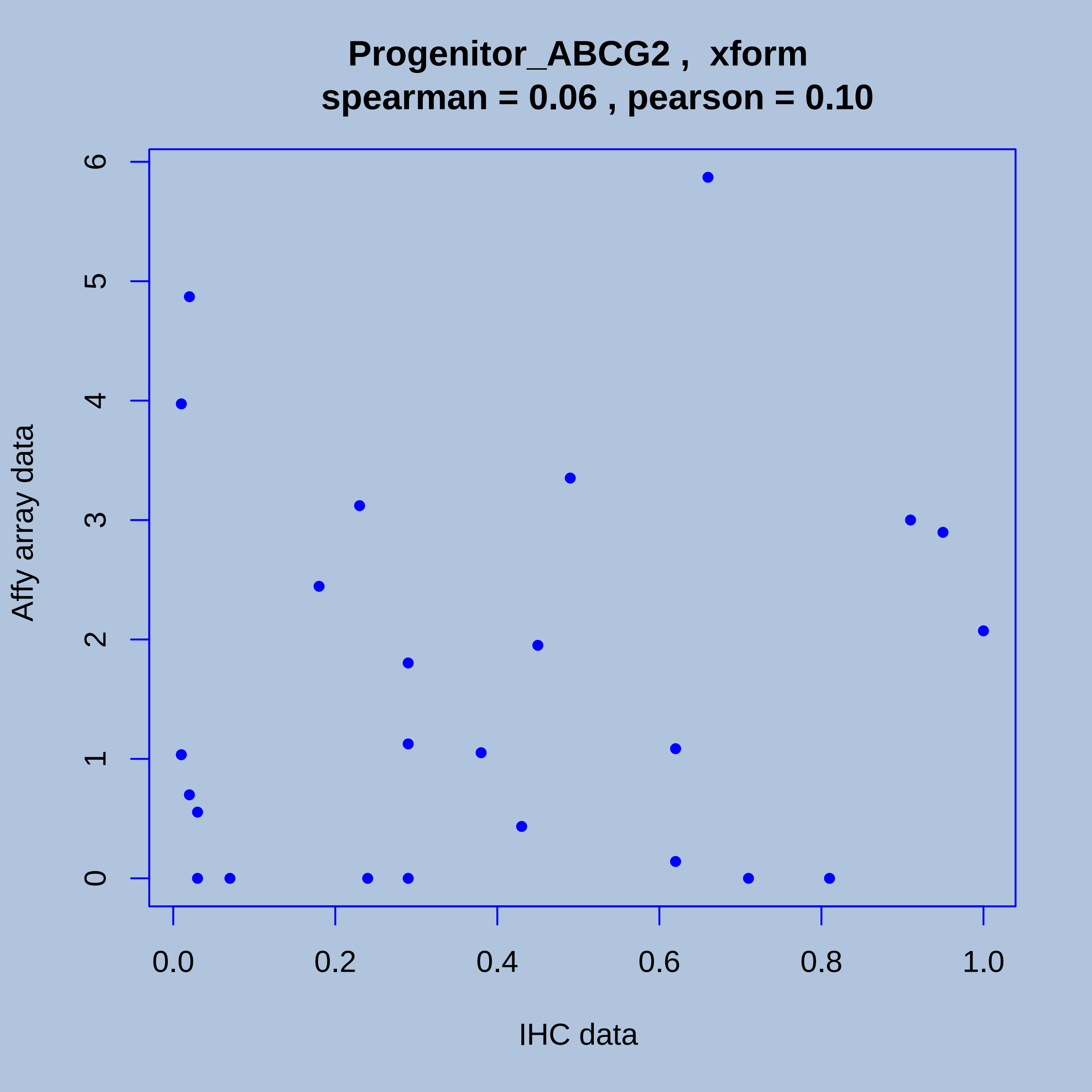
Progenitor_ABCG2 , xform
spearman = 0.06 , pearson = 0.10
Affy array data
IHC data
0
1
2
3
4
5
6
0.0
0.2
0.4
0.6
0.8
1.0

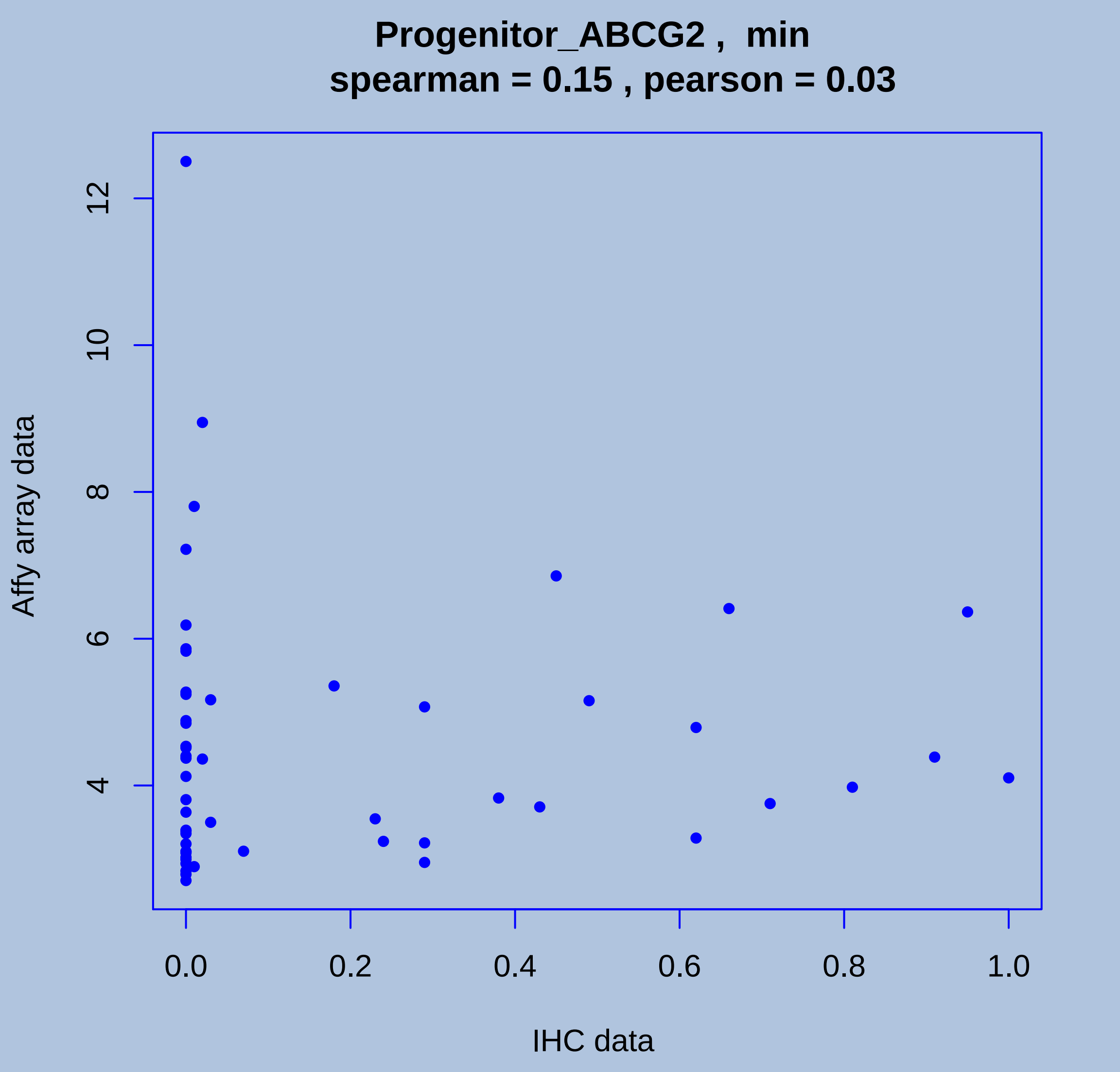
Progenitor_ABCG2 , min
spearman = 0.15 , pearson = 0.03
Affy array data
IHC data
4
6
8
10
12
0.0
0.2
0.4
0.6
0.8
1.0

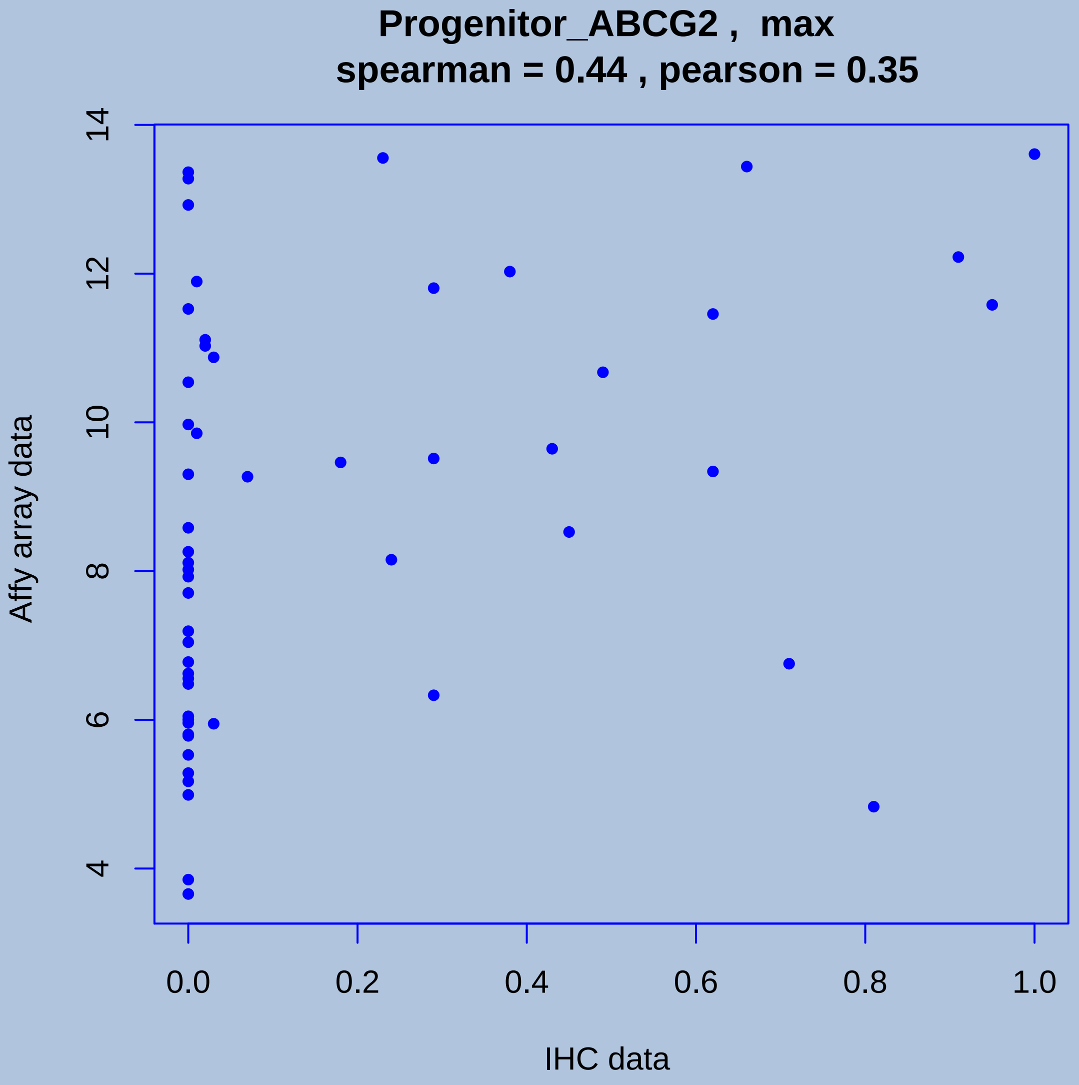

Progenitor_ABCG2 , max
spearman = 0.44 , pearson = 0.35
Affy array data
4
6
8
10
12
14
0.0
0.2
0.4
0.6
0.8
1.0
IHC data

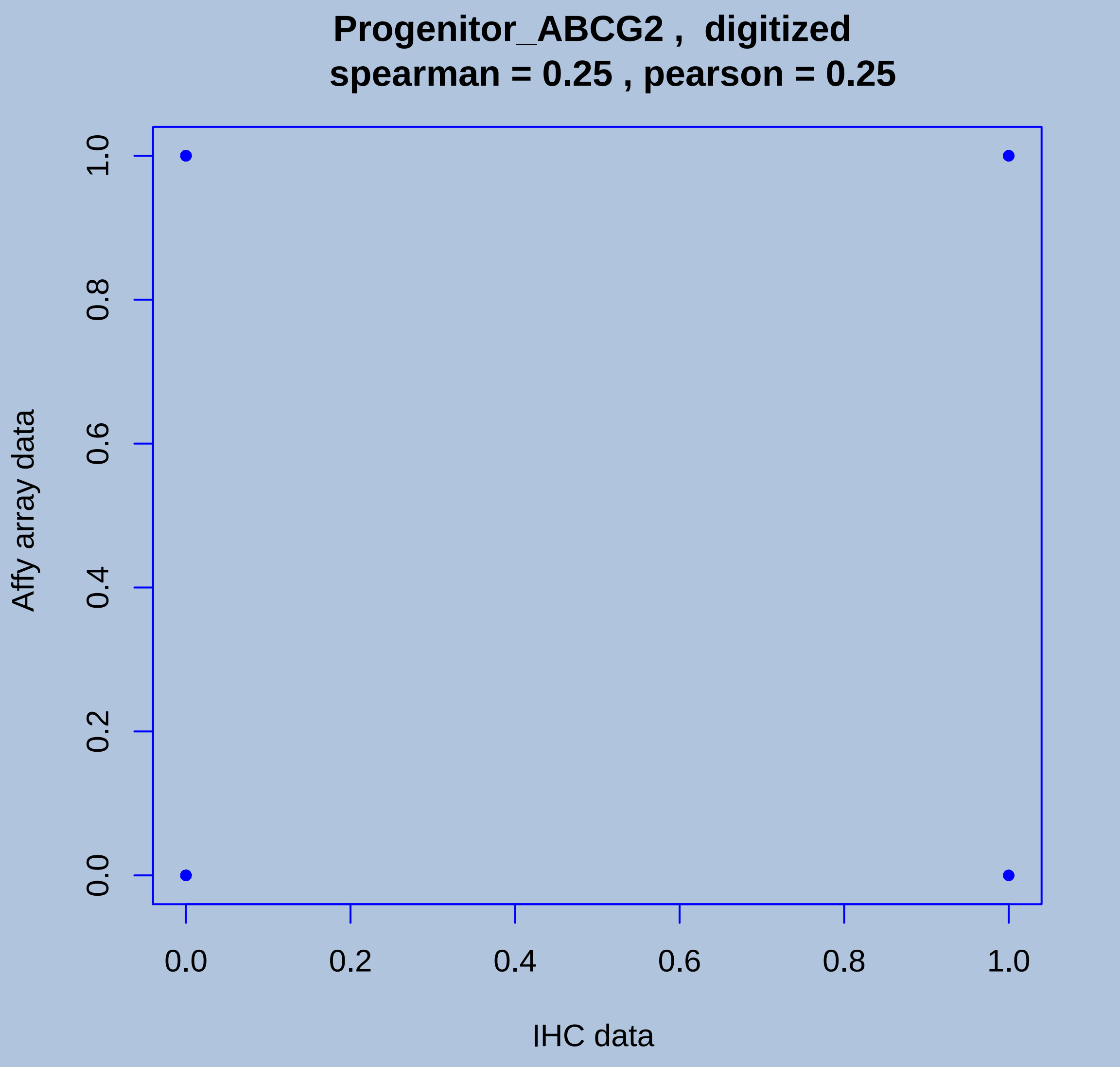
Progenitor_ABCG2 , digitized
spearman = 0.25 , pearson = 0.25
Affy array data
IHC data
0.0
0.2
0.4
0.6
0.8
1.0
0.0
0.2
0.4
0.6
0.8
1.0

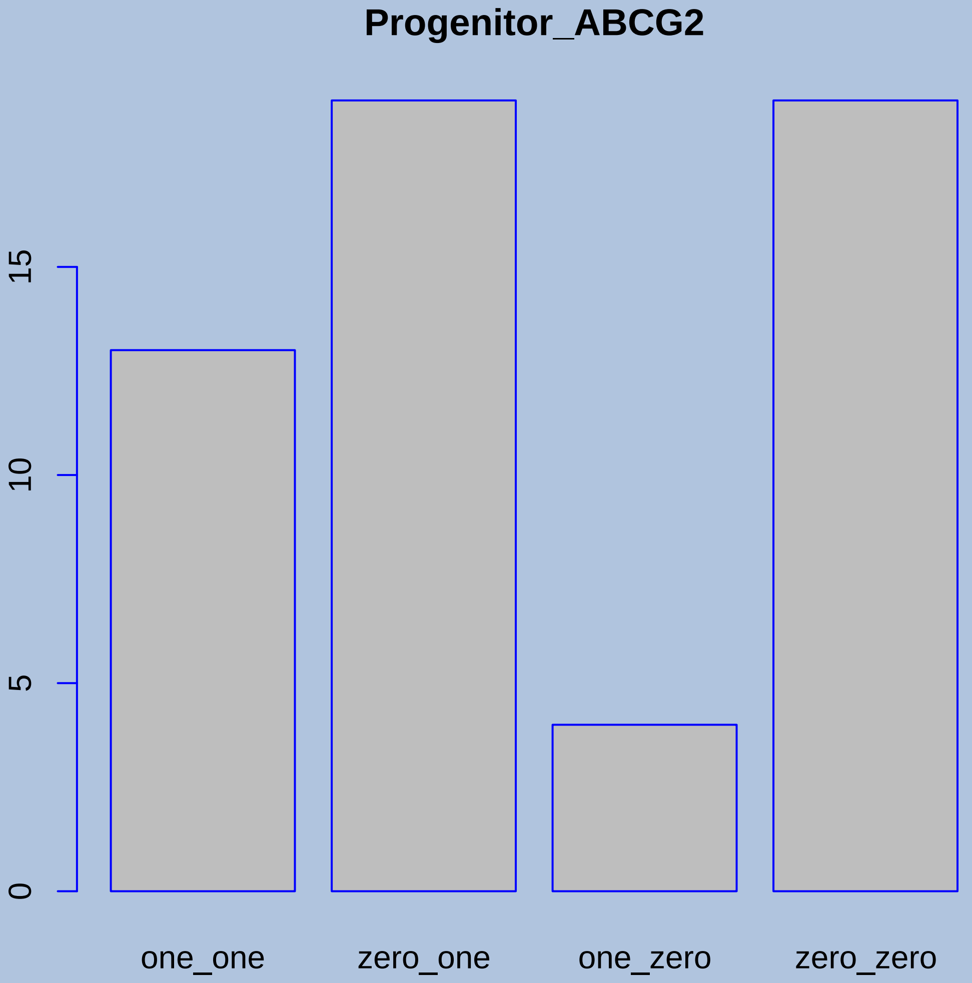
Progenitor_ABCG2
0
5
10
15
one_one
zero_one
one_zero
zero_zero

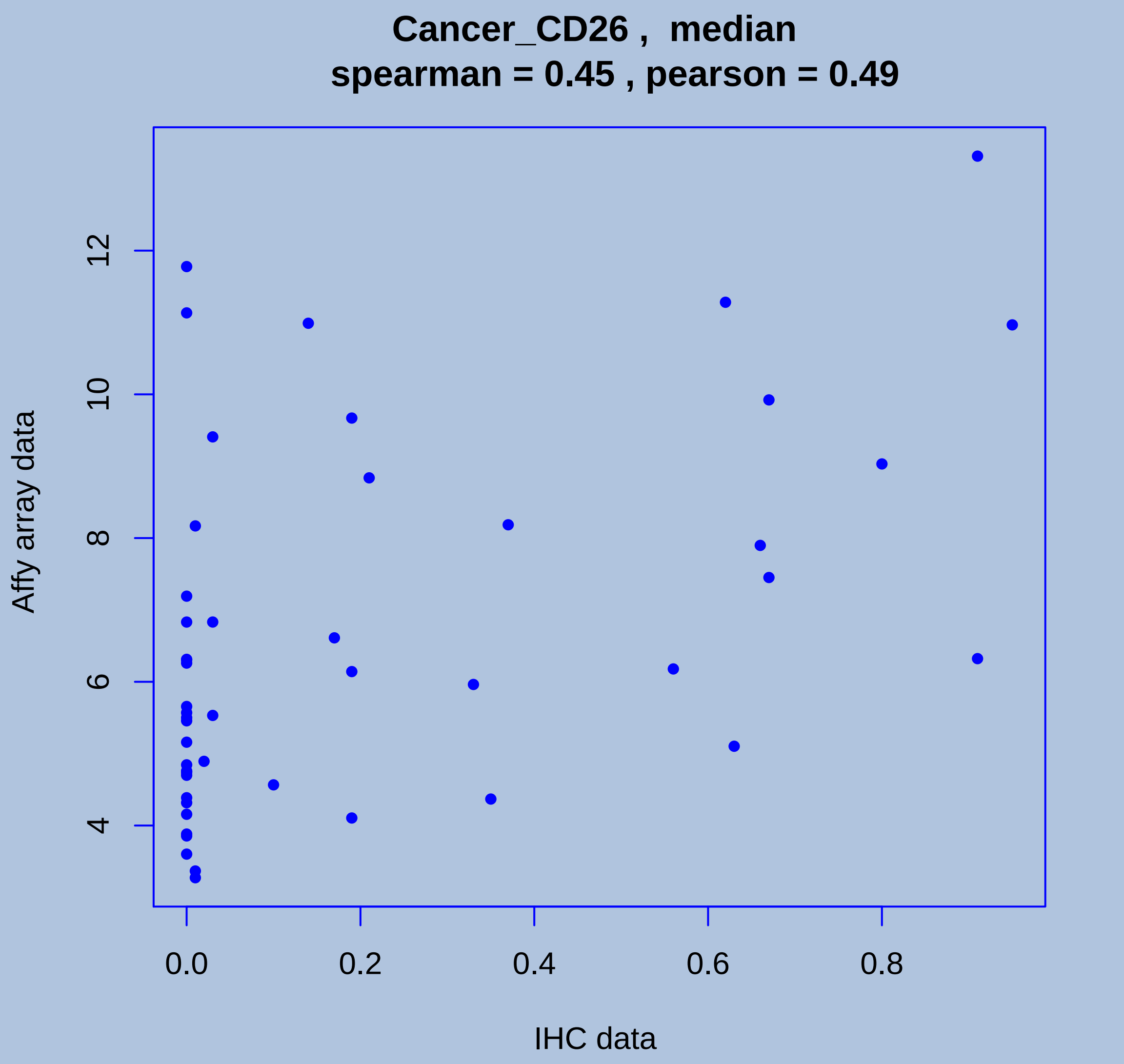

Cancer_CD26 , median
spearman = 0.45 , pearson = 0.49
Affy array data
IHC data
4
6
8
10
12
0.0
0.2
0.4
0.6
0.8

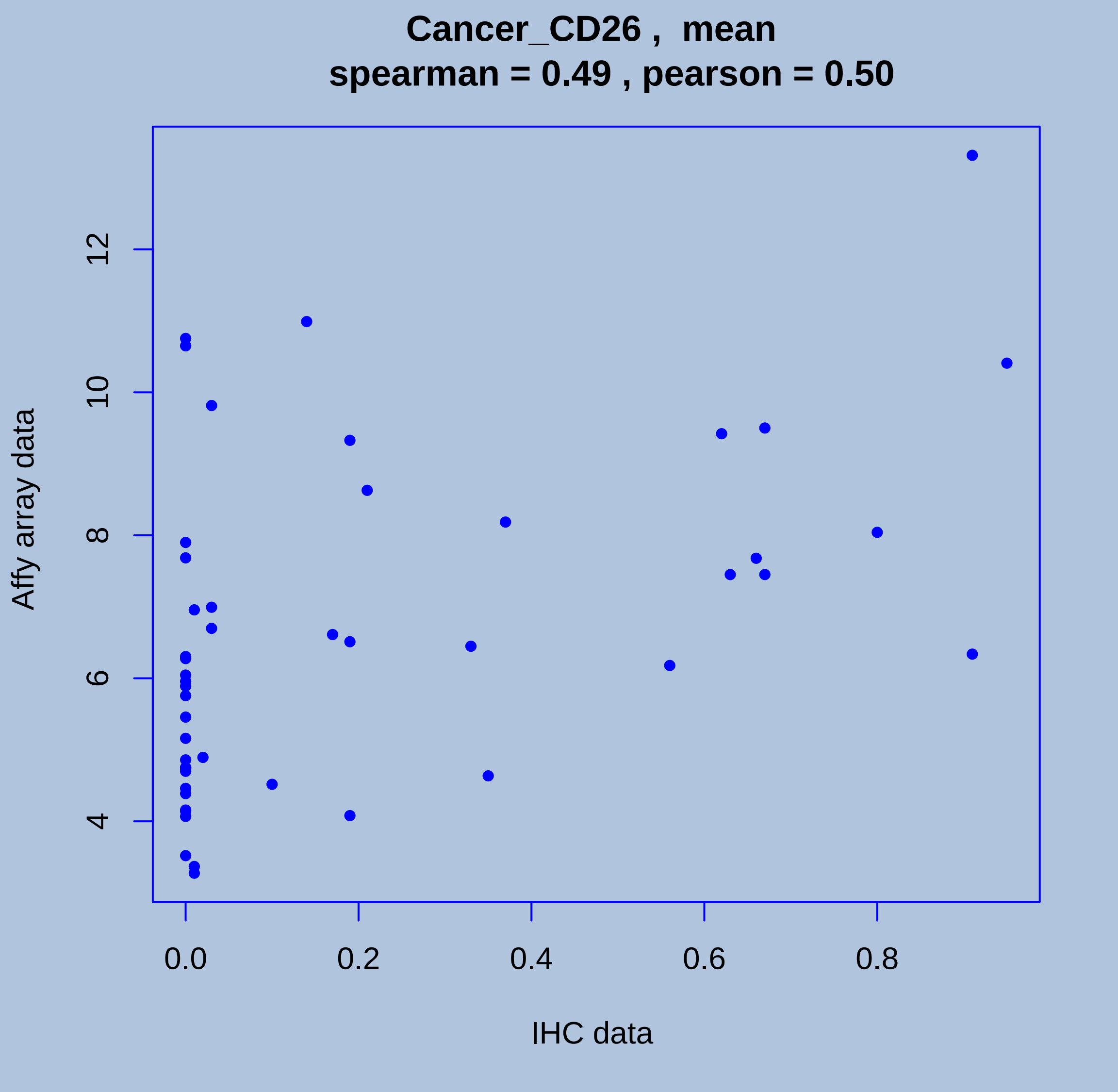
Cancer_CD26 , mean
spearman = 0.49 , pearson = 0.50
Affy array data
4
6
8
10
12
0.0
0.2
0.4
0.6
0.8
IHC data

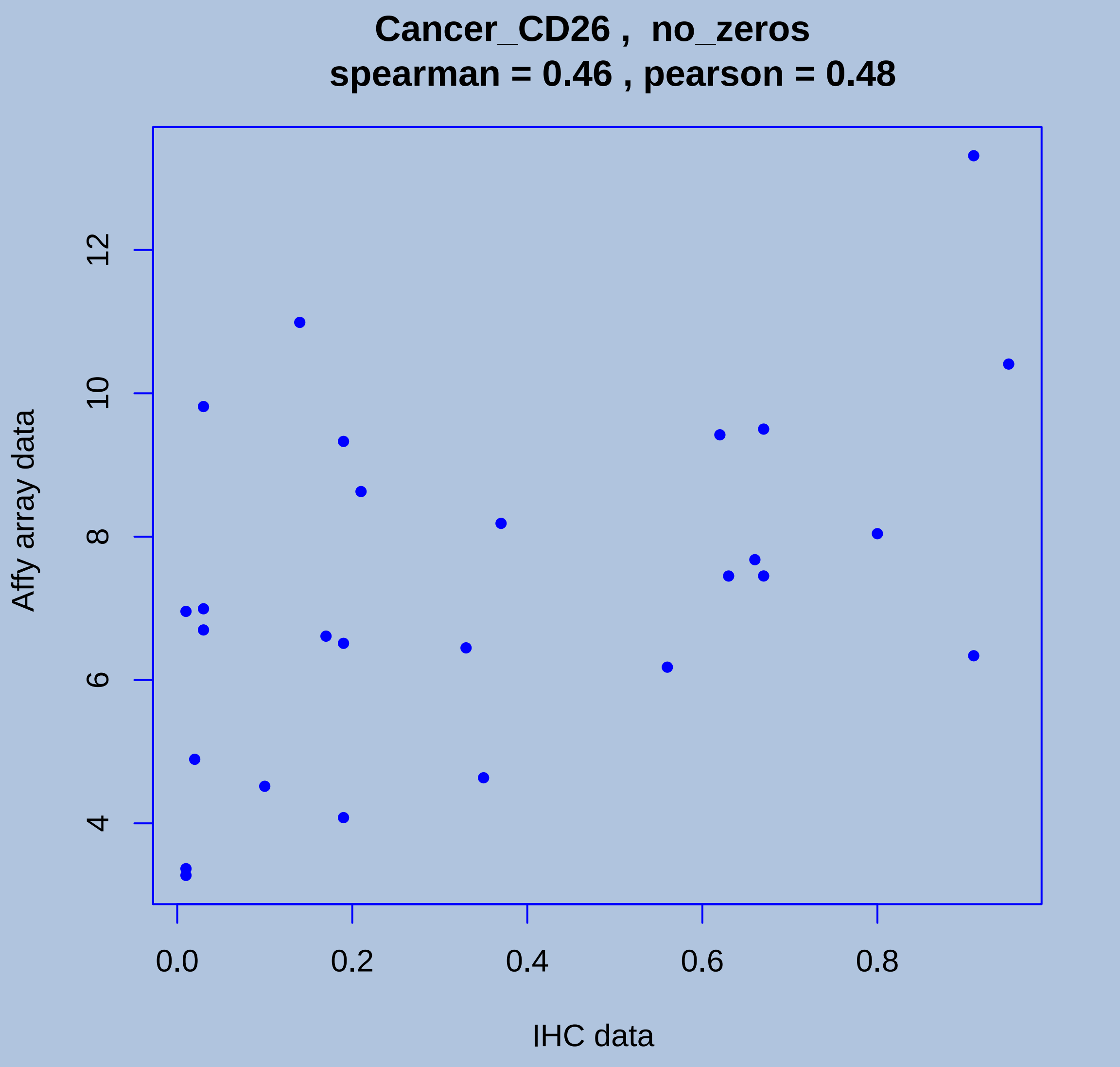

Cancer_CD26 , no_zeros
spearman = 0.46 , pearson = 0.48
Affy array data
IHC data
4
6
8
10
12
0.0
0.2
0.4
0.6
0.8

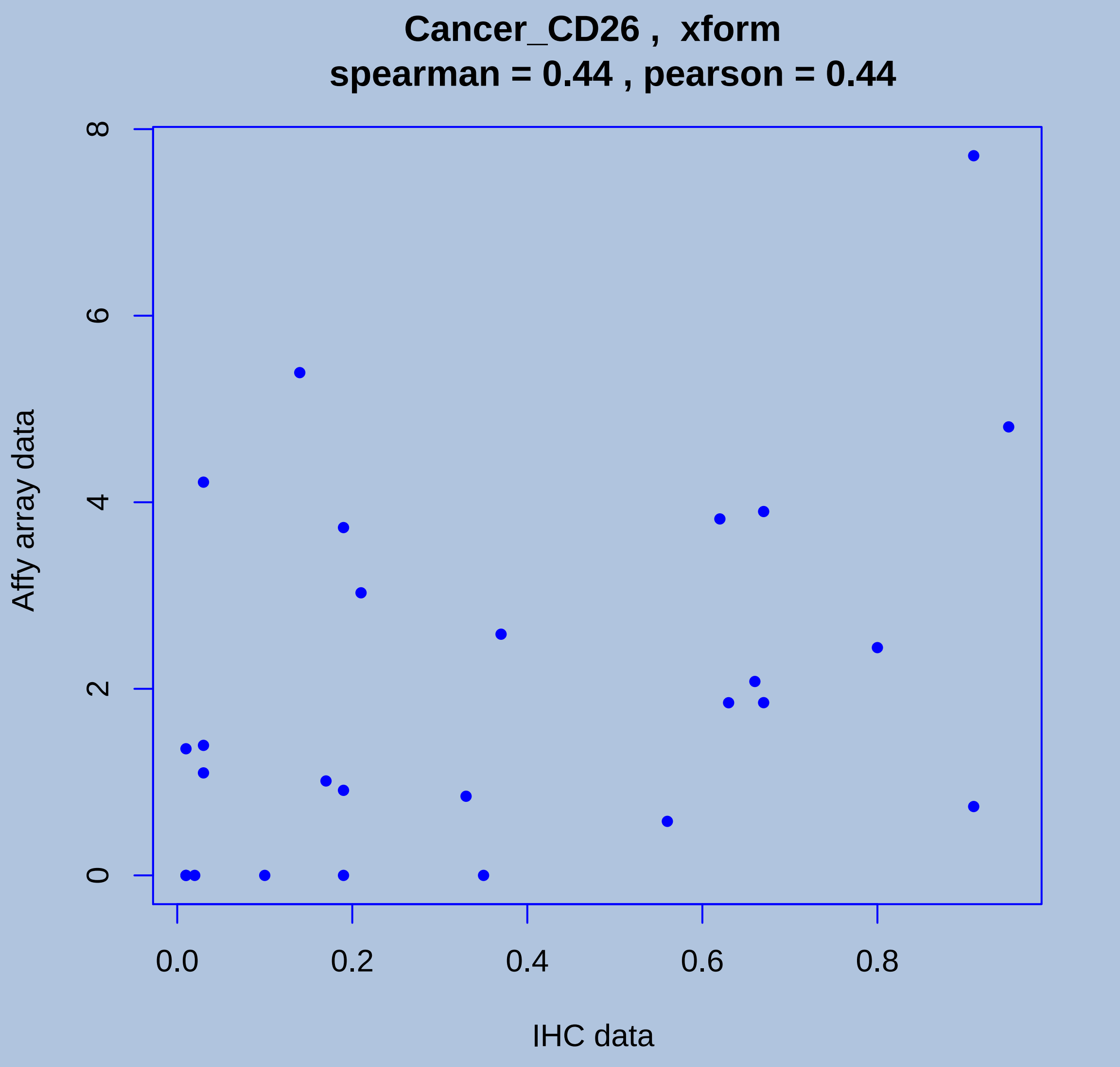

Cancer_CD26 , xform
spearman = 0.44 , pearson = 0.44
Affy array data
IHC data
0
2
4
6
8
0.0
0.2
0.4
0.6
0.8

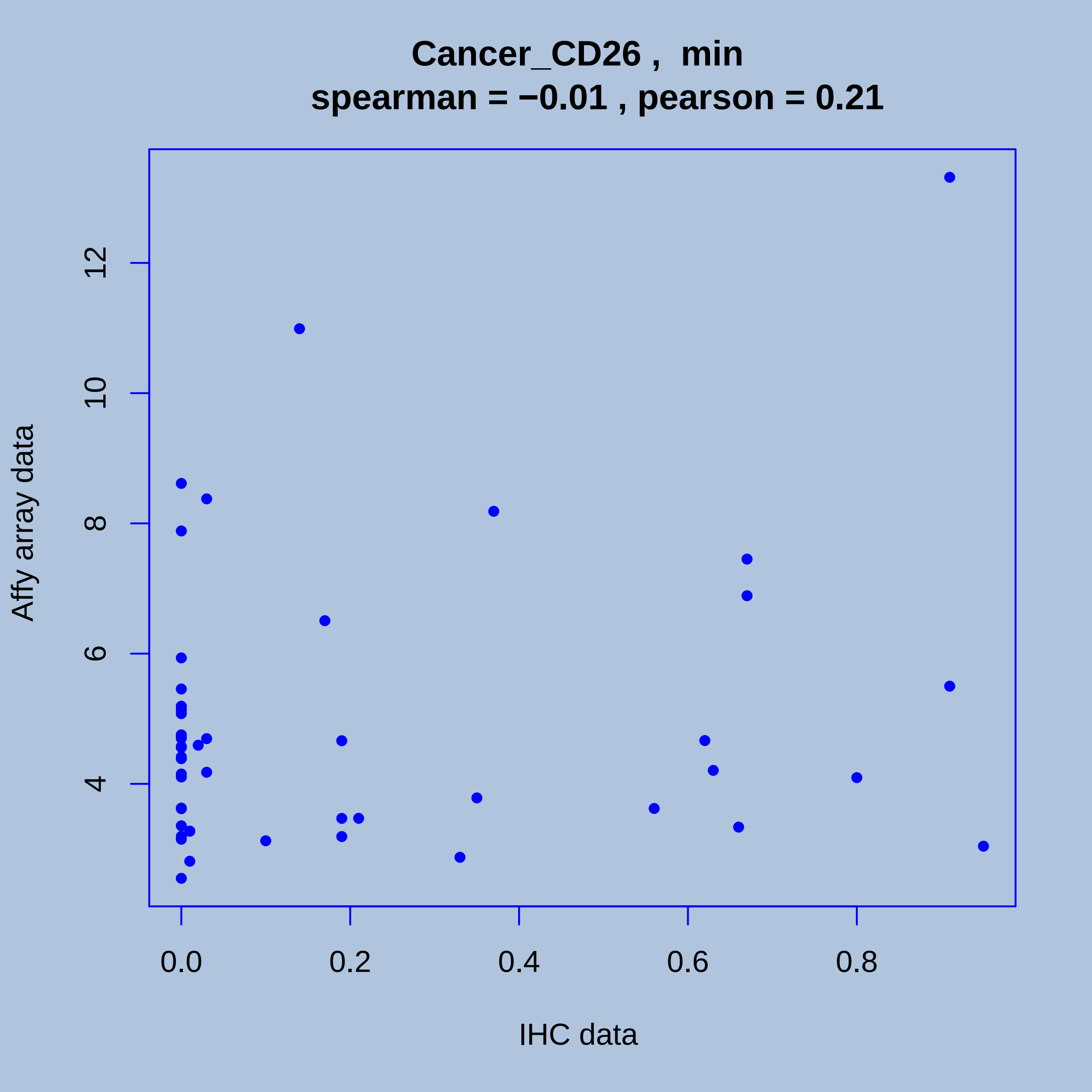

Cancer_CD26 , min
spearman = −0.01 , pearson = 0.21
Affy array data
IHC data
4
6
8
10
12
0.0
0.2
0.4
0.6
0.8

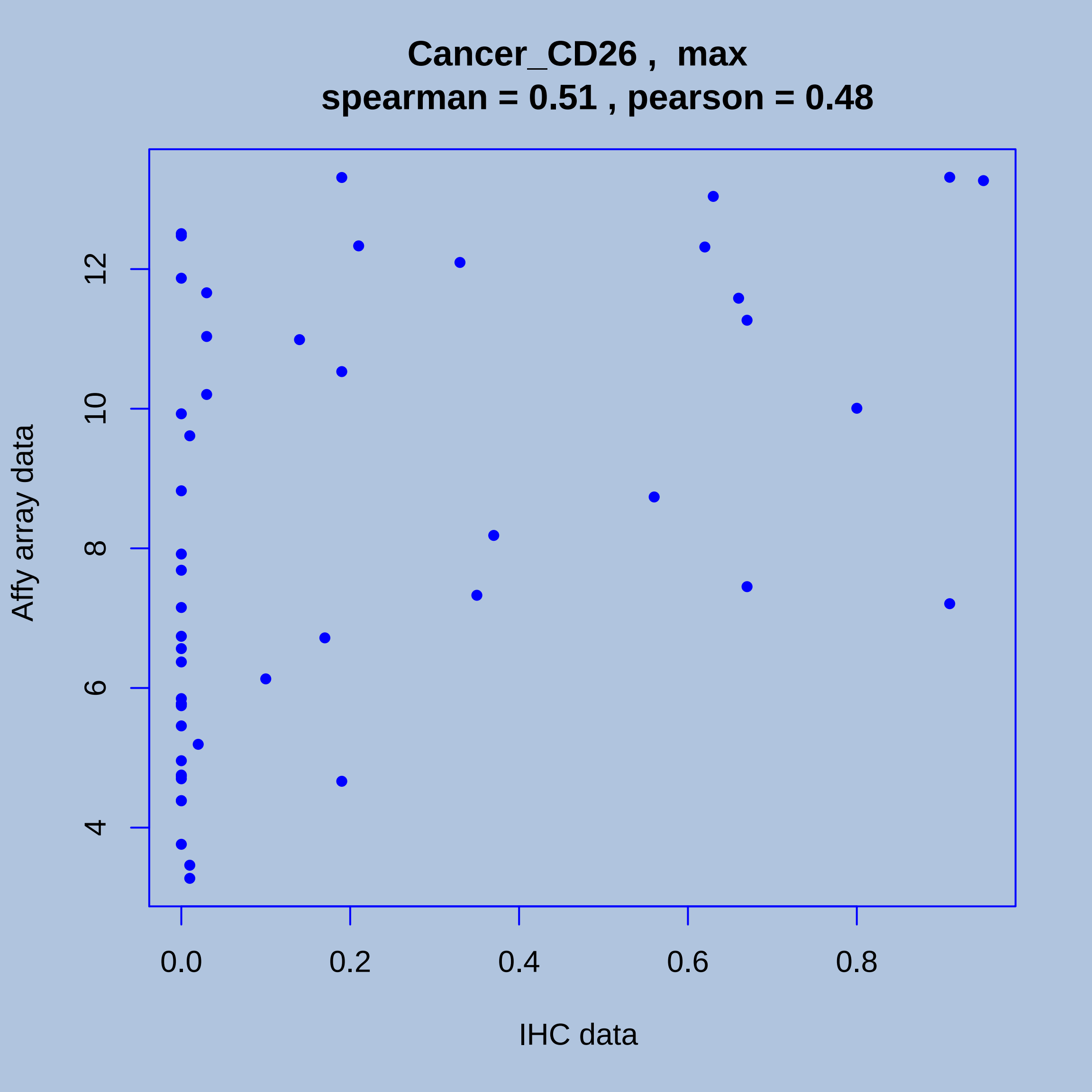
Cancer_CD26 , max
spearman = 0.51 , pearson = 0.48
Affy array data
4
6
8
10
12
0.0
0.2
0.4
0.6
0.8
IHC data

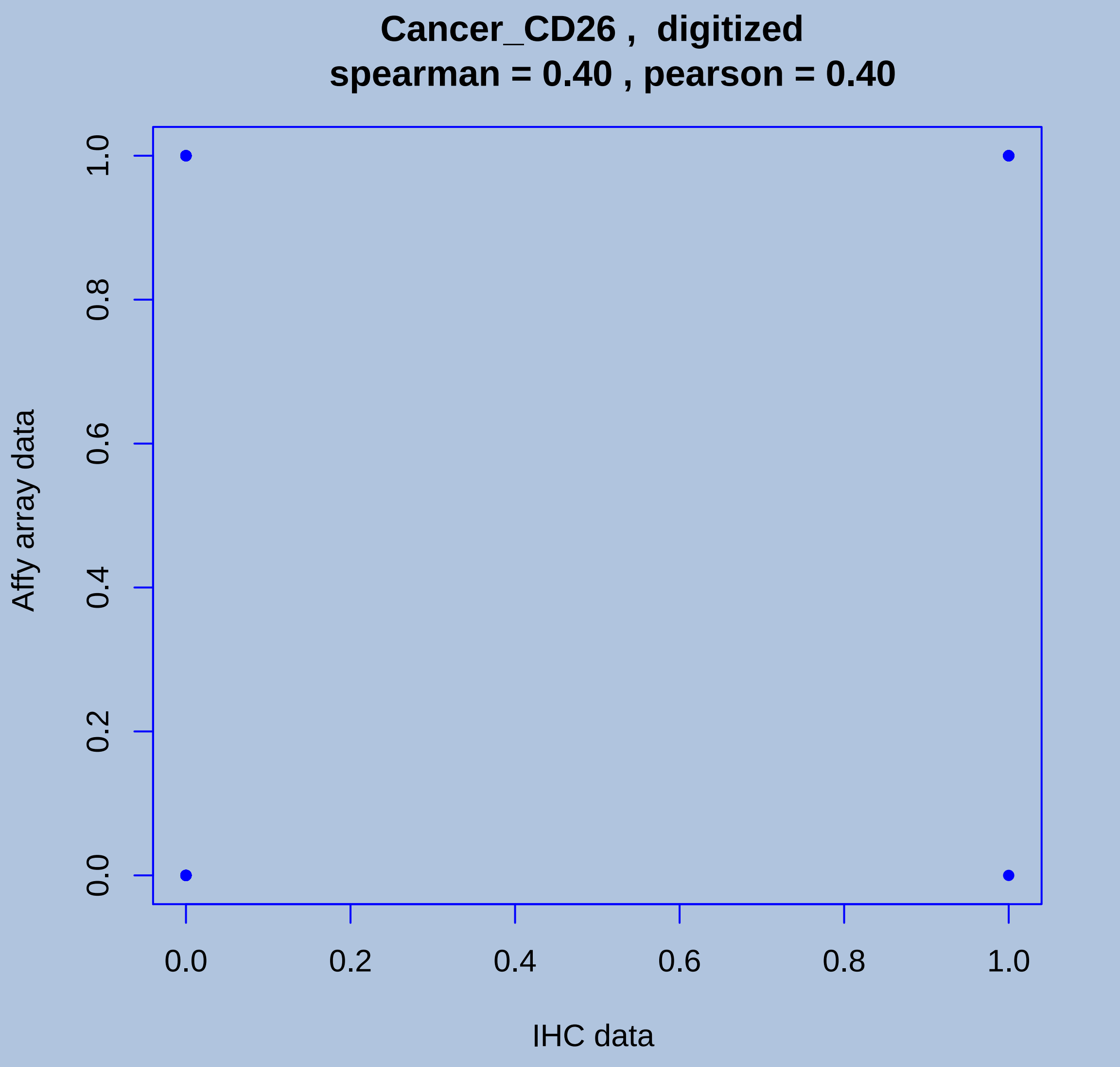
Cancer_CD26 , digitized
spearman = 0.40 , pearson = 0.40
Affy array data
IHC data
0.0
0.2
0.4
0.6
0.8
1.0
0.0
0.2
0.4
0.6
0.8
1.0

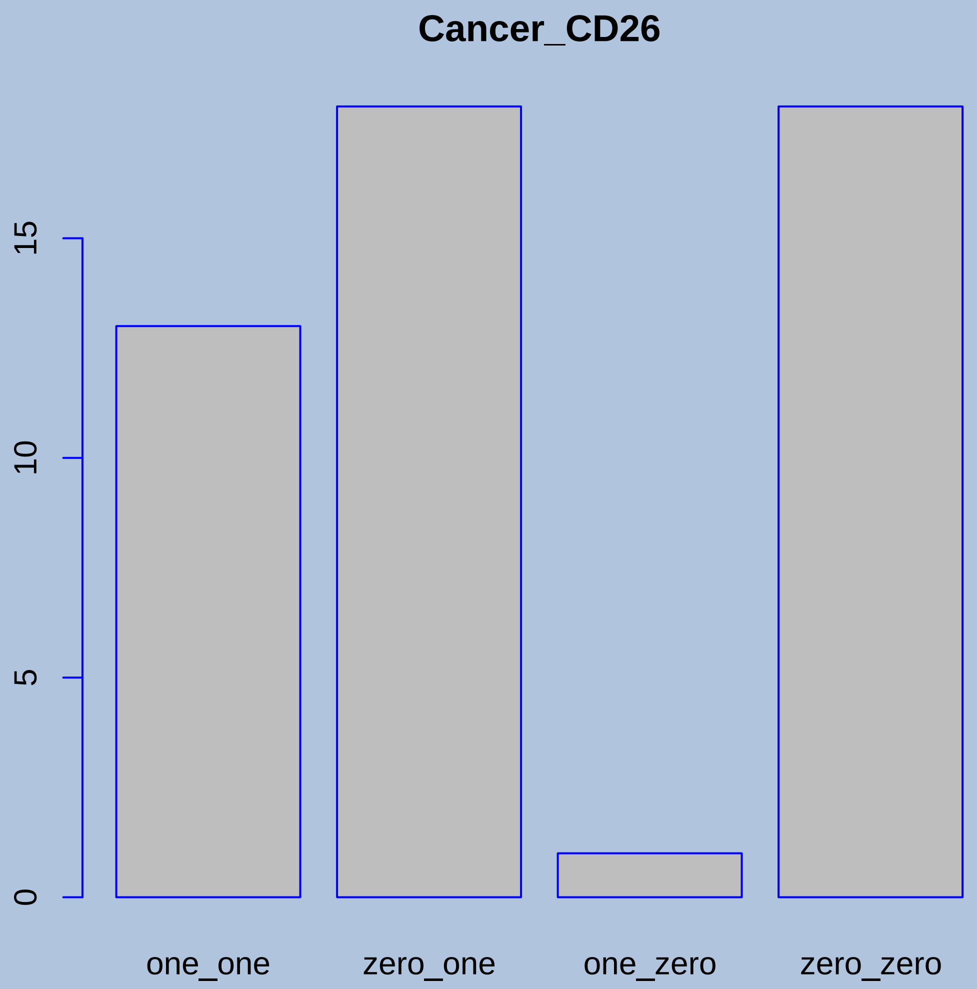
Cancer_CD26
0
5
10
15
one_one
zero_one
one_zero
zero_zero
