# Supplementary material for: Correlation of mRNA and protein levels: Cell type-specific gene expression of cluster designation antigens in the prostate
Source: BMC Genomics. 2008 May 23;9:246. doi: 10.1186/1471-2164-9-246 (PMC2413246; doi:10.1186/1471-2164-9-246)
Supplement: Additional file 2 — Scatter plot using updated probe set definitions. Statistical Pearson correlations ranged from 0.09 – 0.57 and Spearman Coefficients ranged from 0.03 – 0.63 using updated probe set definitions. Updated probe set definitions increased correlation from none to moderate positive correlation for progenitor cells. [file 1471-2164-9-246-S2.pdf]

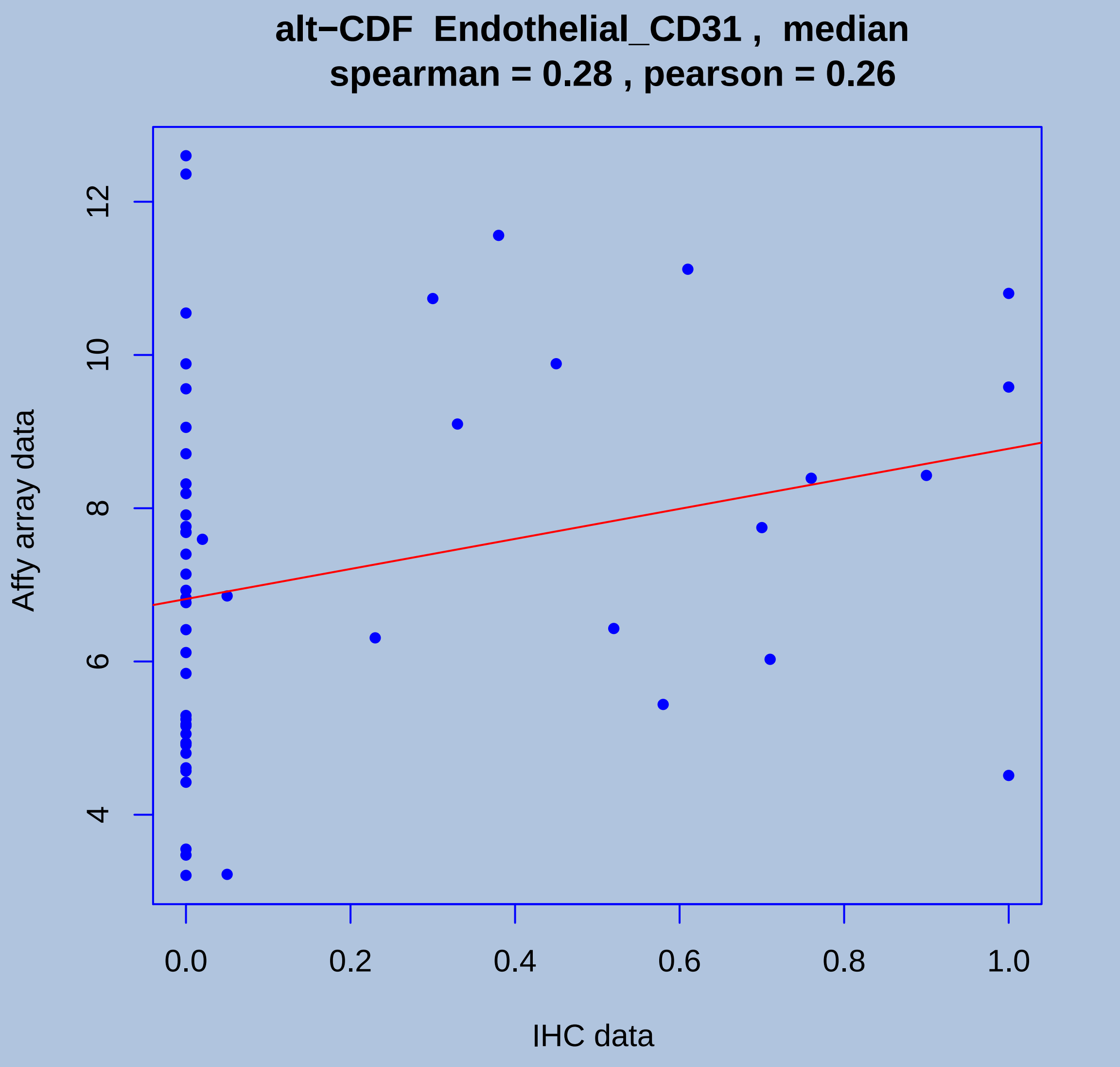
alt−CDF Endothelial_CD31 , median
spearman = 0.28 , pearson = 0.26
Affy array data
4
6
8
10
12
IHC data
0.0
0.2
0.4
0.6
0.8
1.0

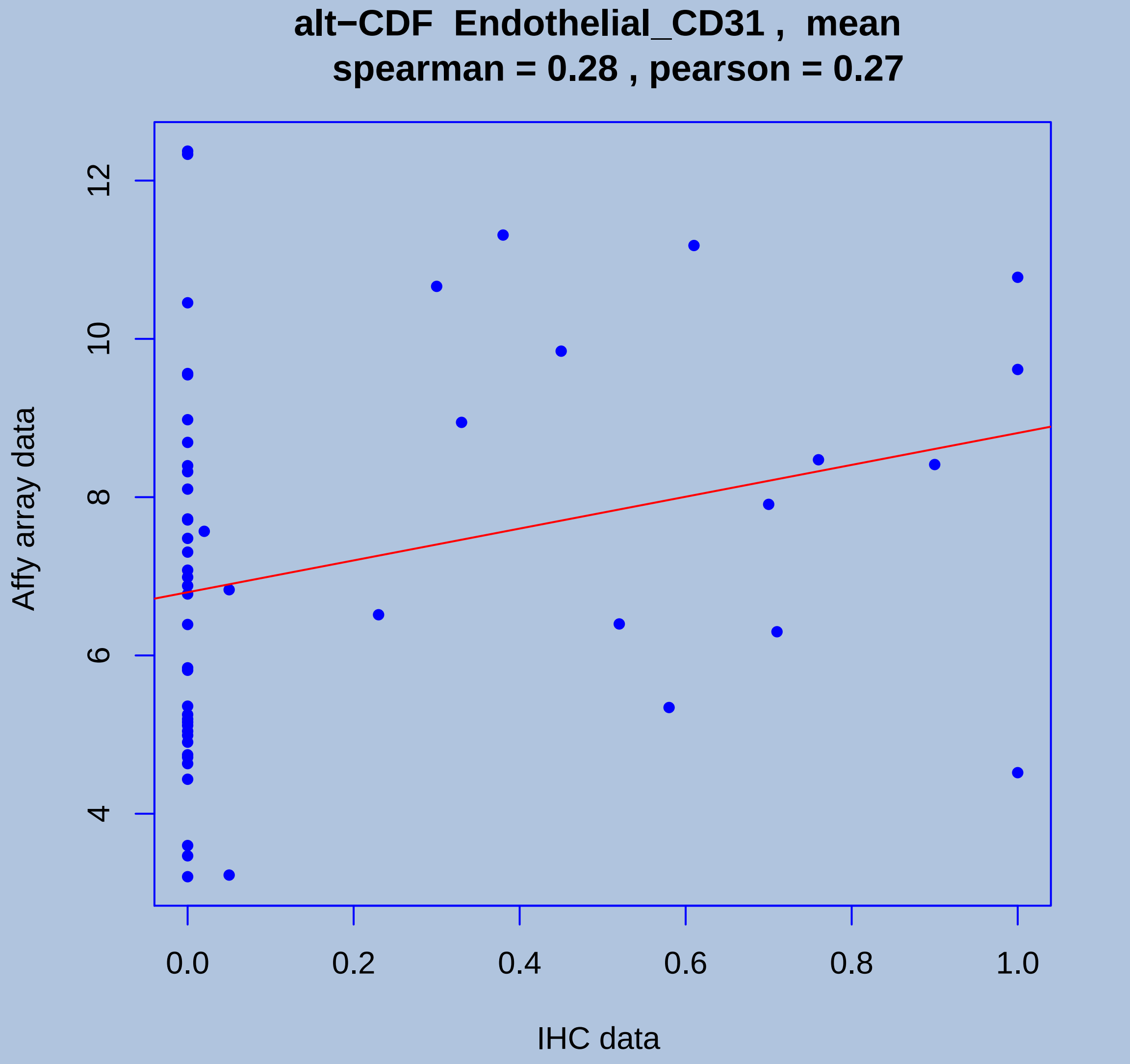

alt−CDF Endothelial_CD31 , mean
spearman = 0.28 , pearson = 0.27
Affy array data
IHC data
4
6
8
10
12
0.0
0.2
0.4
0.6
0.8
1.0

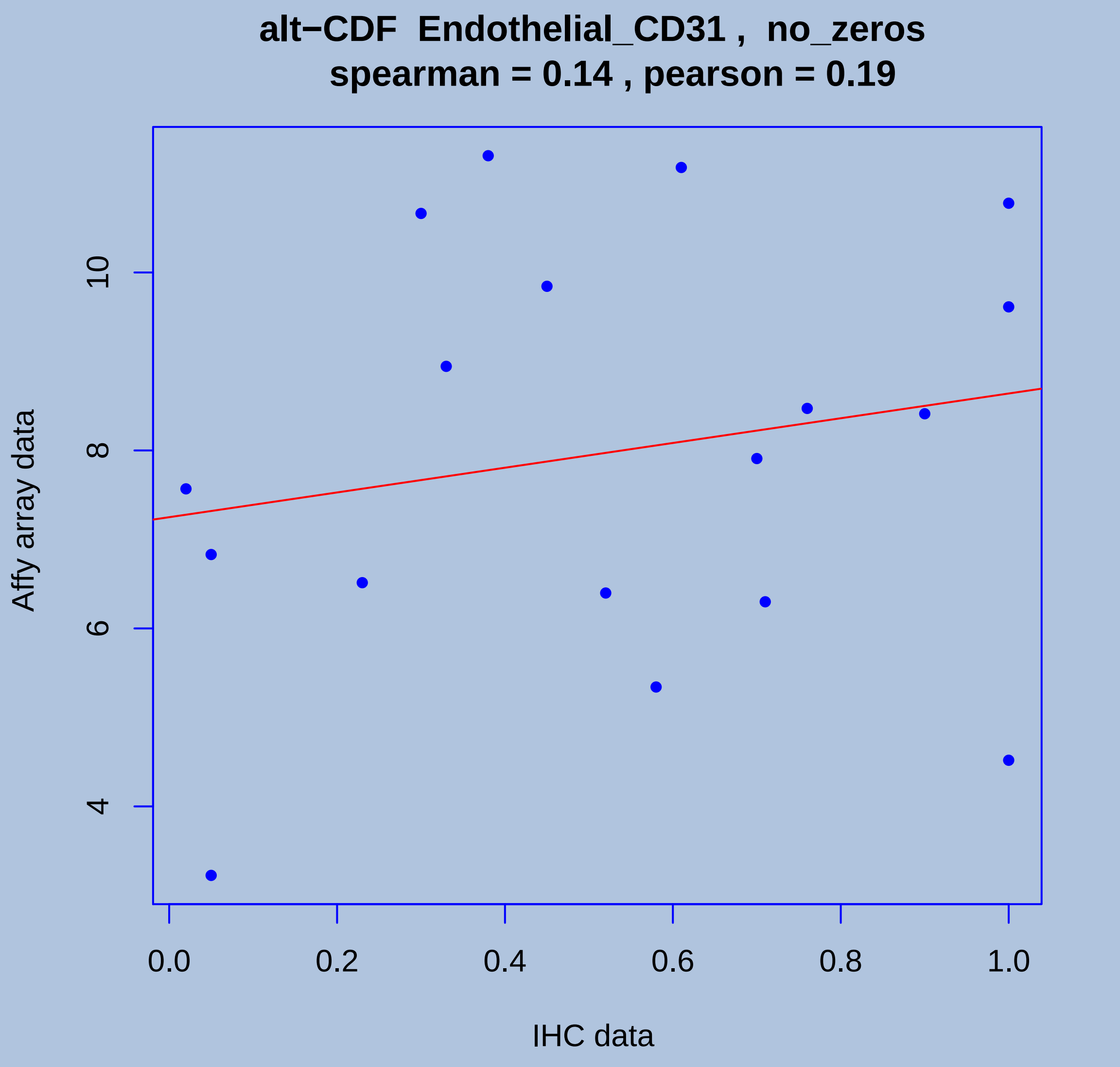
alt−CDF Endothelial_CD31 , no_zeros
spearman = 0.14 , pearson = 0.19
Affy array data
4
6
8
10
0.0
0.2
0.4
0.6
0.8
1.0
IHC data

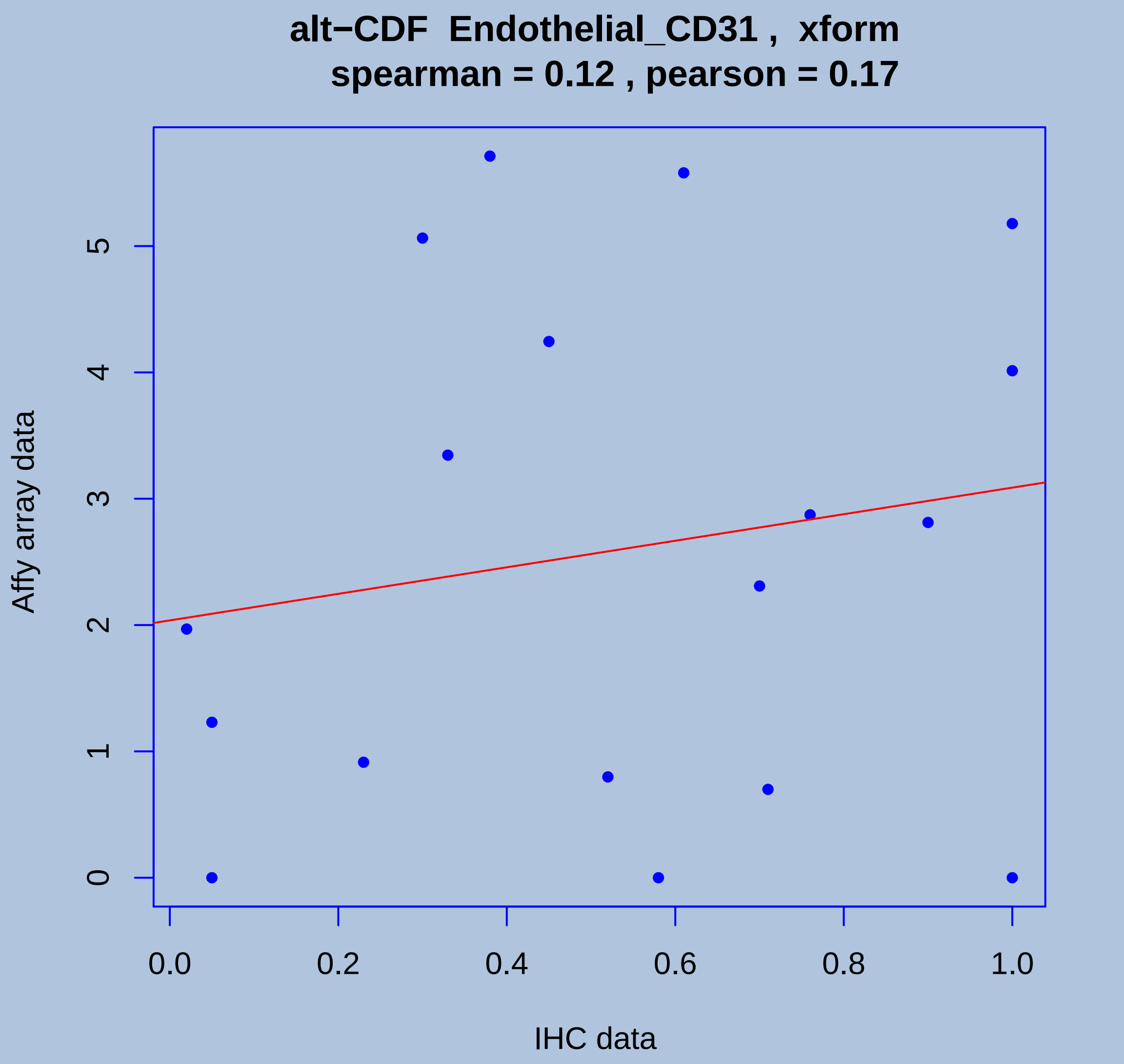
alt−CDF Endothelial_CD31 , xform
spearman = 0.12 , pearson = 0.17
Affy array data
0
1
2
3
4
5
0.0
0.2
0.4
0.6
0.8
1.0
IHC data

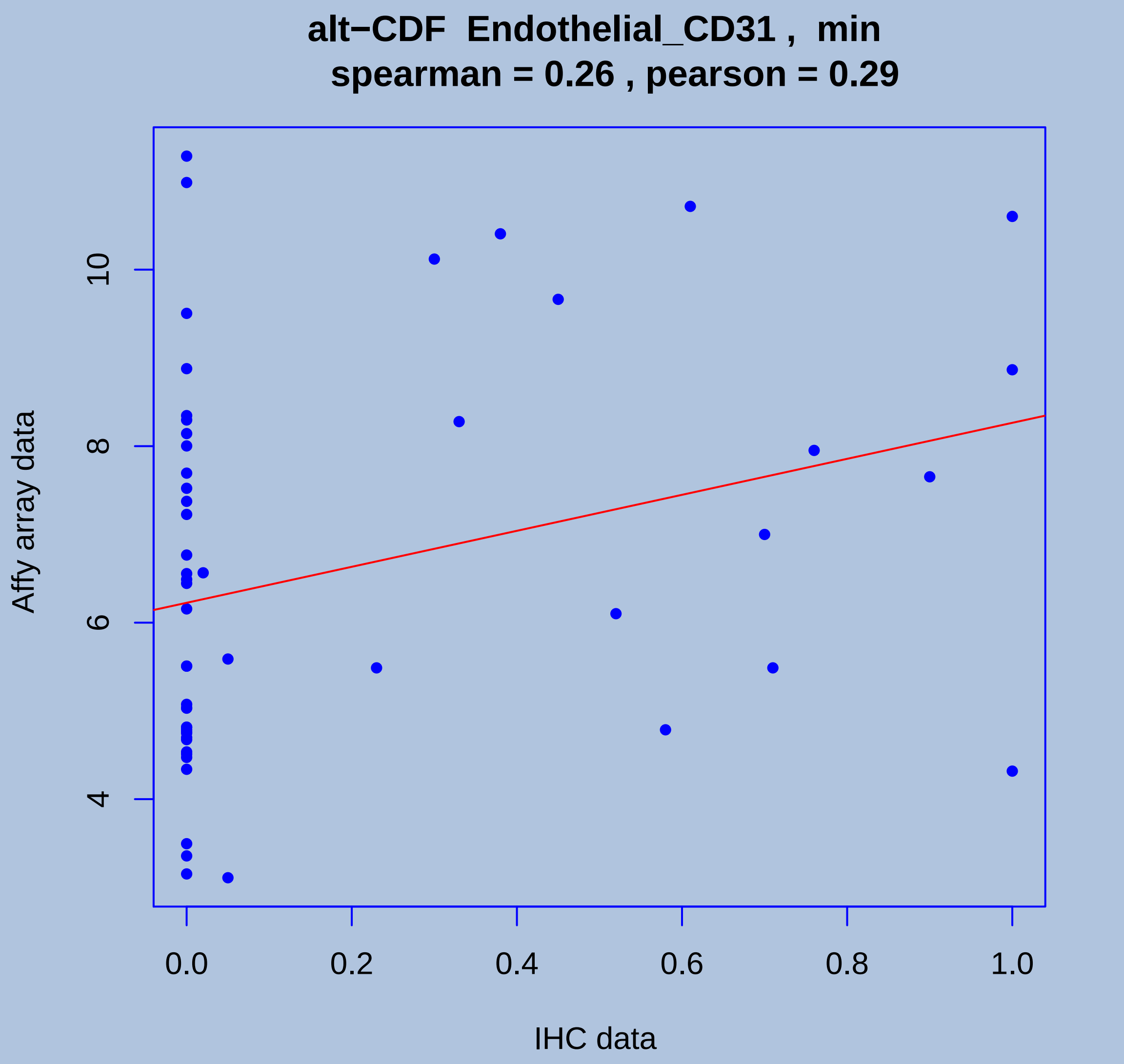
alt−CDF Endothelial_CD31 , min
spearman = 0.26 , pearson = 0.29
Affy array data
4
6
8
10
0.0
0.2
0.4
0.6
0.8
1.0
IHC data

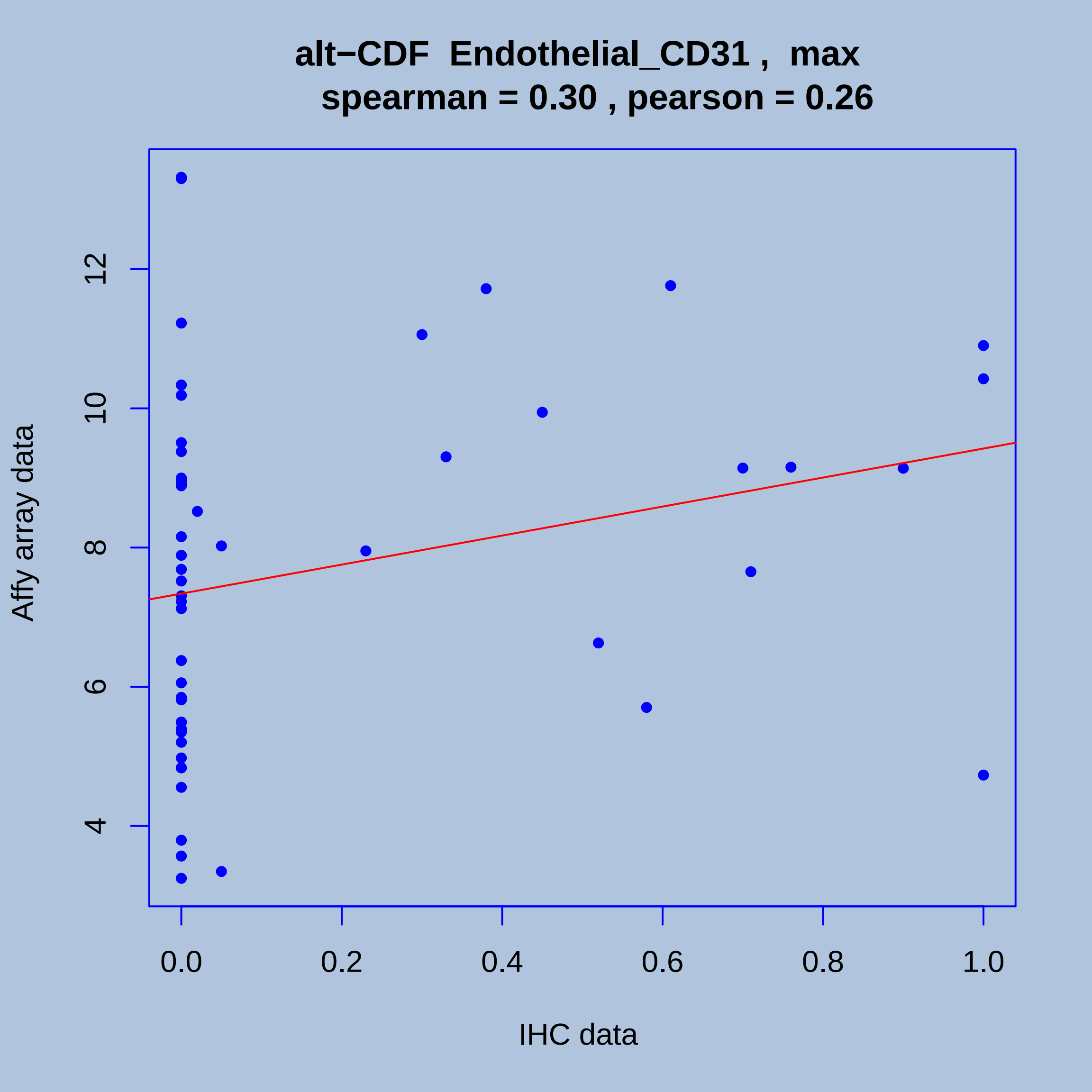
alt−CDF Endothelial_CD31 , max
spearman = 0.30 , pearson = 0.26
Affy array data
4
6
8
10
12
0.0
0.2
0.4
0.6
0.8
1.0
IHC data

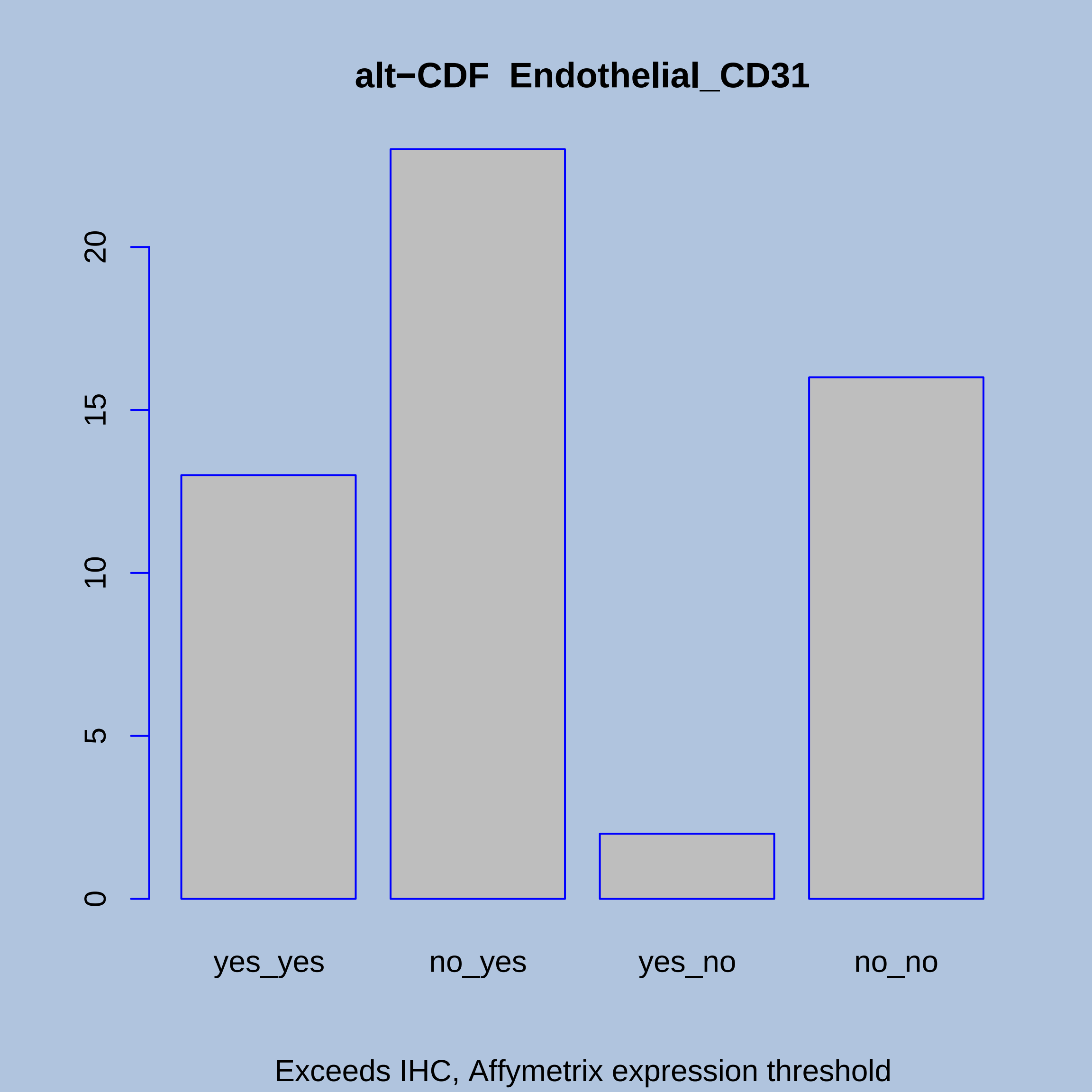
alt−CDF Endothelial_CD31
0
5
10
15
20
yes_yes
no_yes
yes_no
no_no
Exceeds IHC, Affymetrix expression threshold

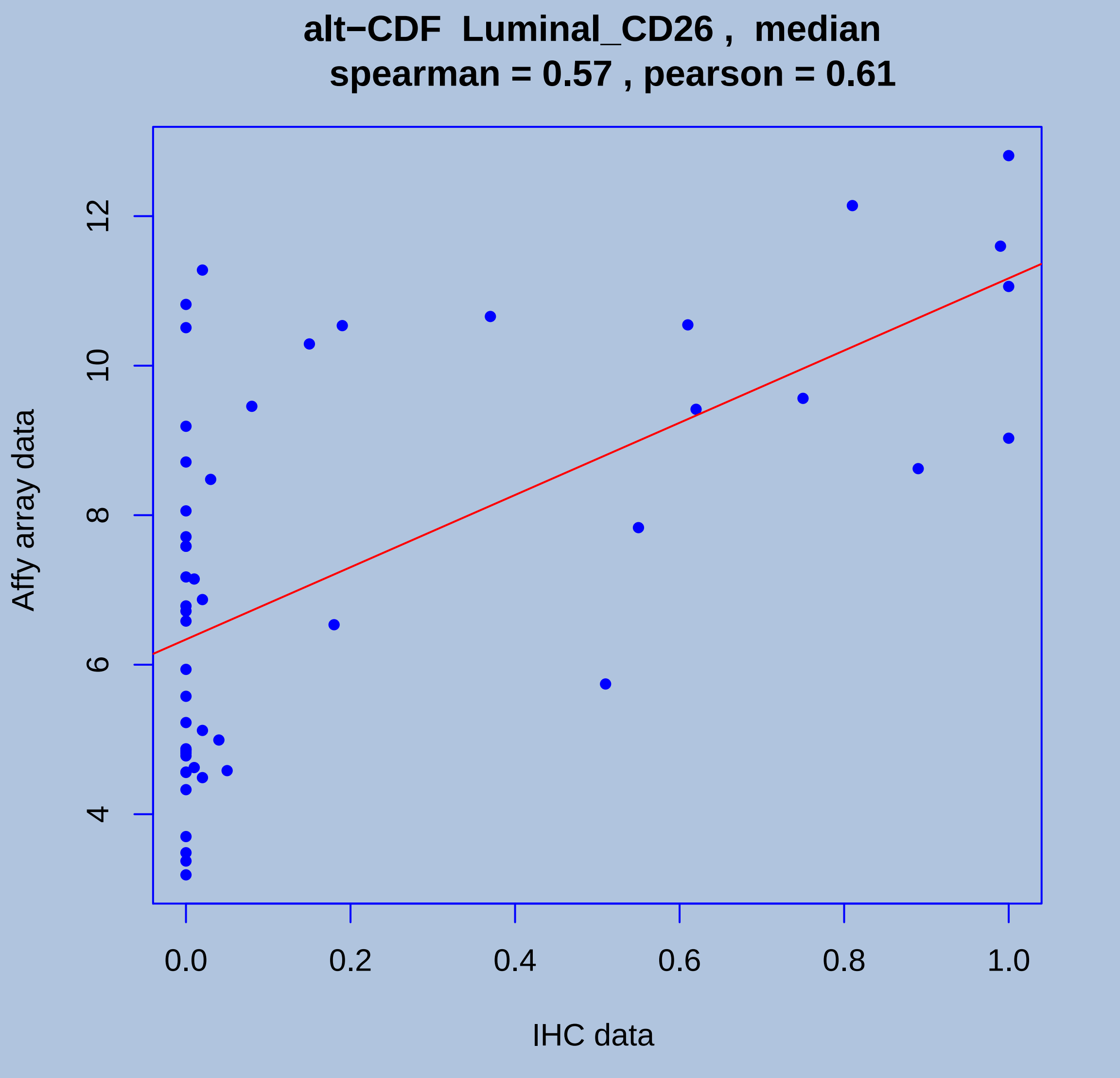

alt−CDF Luminal_CD26 , median
spearman = 0.57 , pearson = 0.61
Affy array data
IHC data
4
6
8
10
12
0.0
0.2
0.4
0.6
0.8
1.0

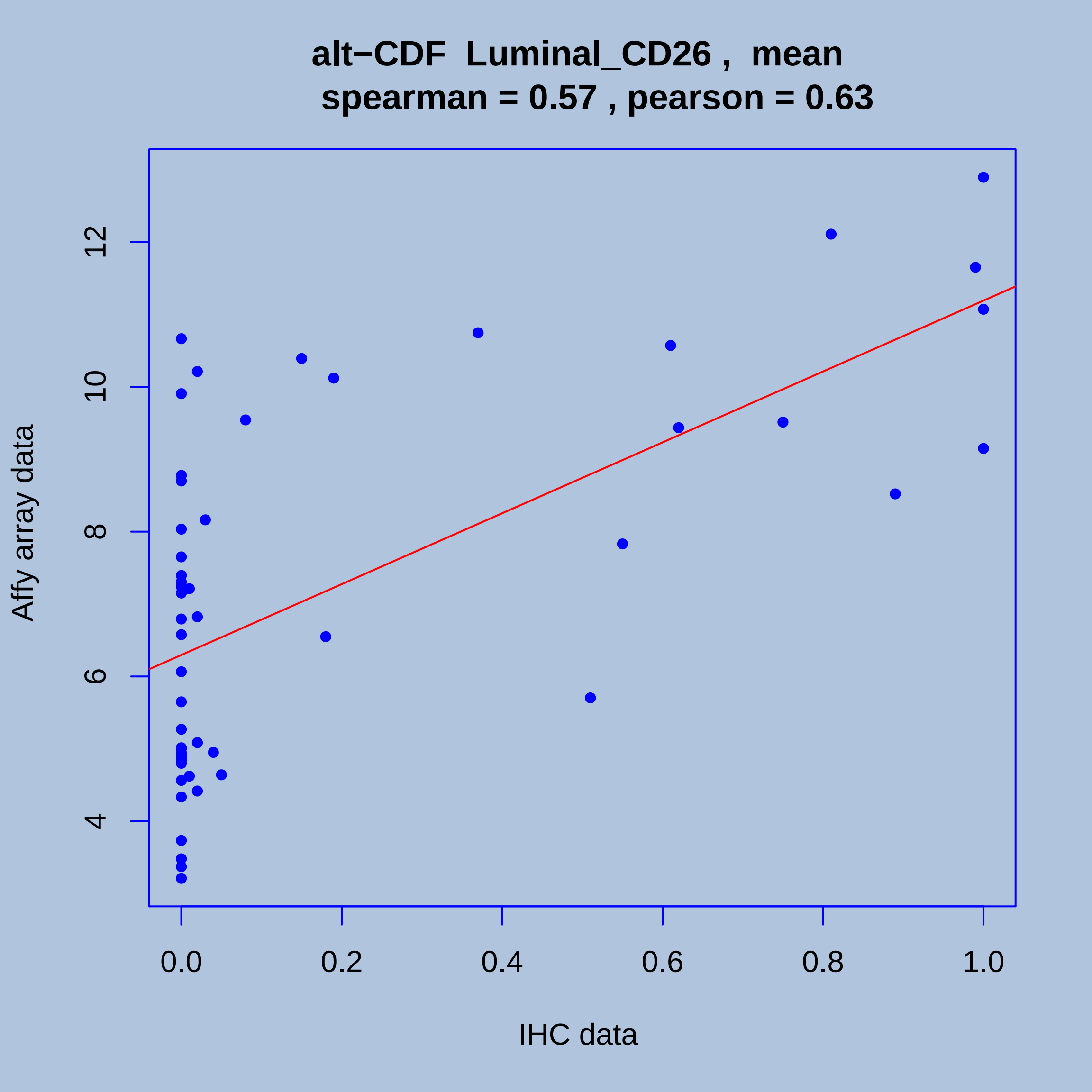

alt−CDF Luminal_CD26 , mean
spearman = 0.57 , pearson = 0.63
Affy array data
IHC data
4
6
8
10
12
0.0
0.2
0.4
0.6
0.8
1.0

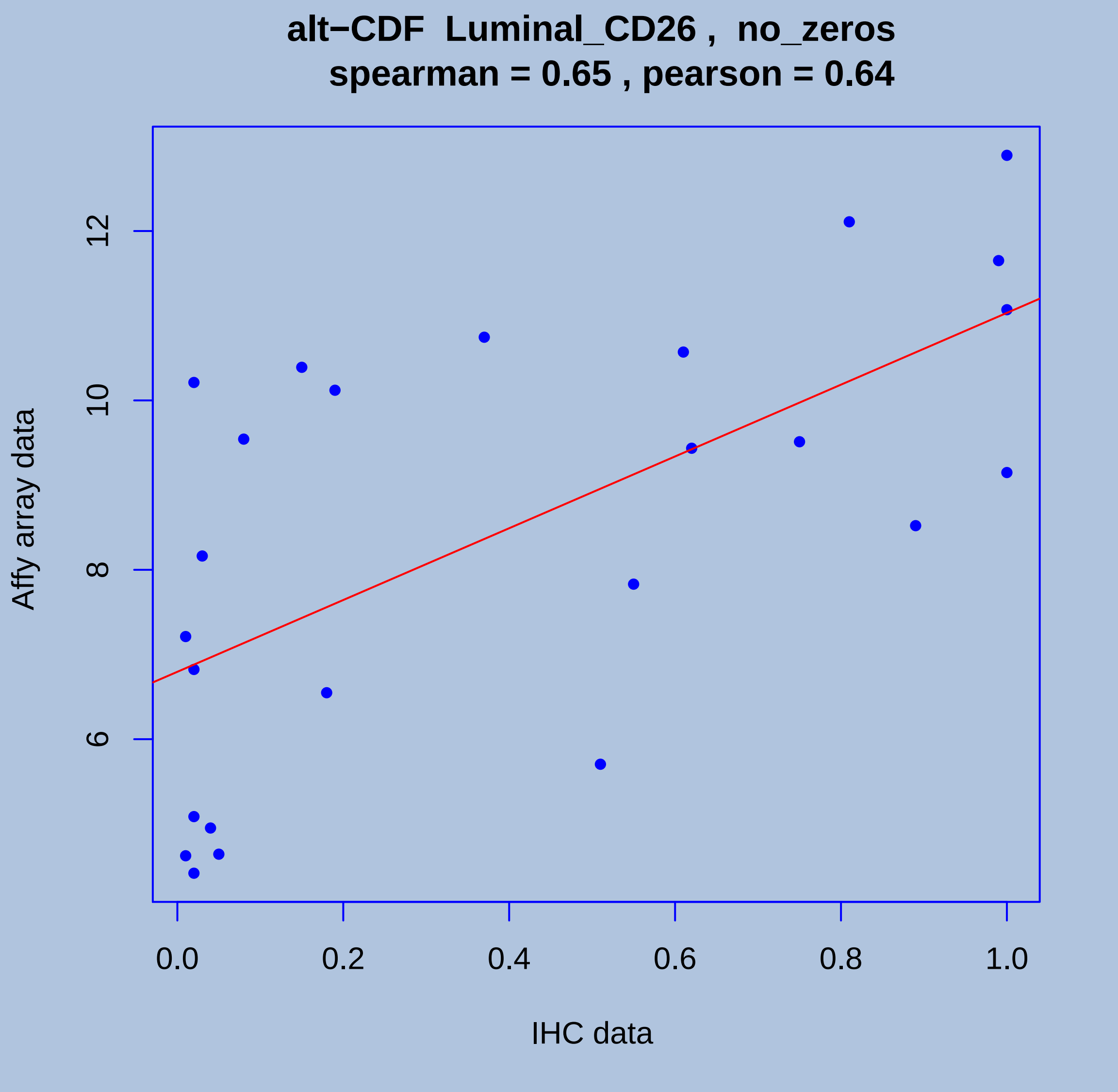

alt−CDF Luminal_CD26 , no_zeros
spearman = 0.65 , pearson = 0.64
12
10
8
6
Affy array data
0.0
0.2
0.4
0.6
0.8
1.0
IHC data

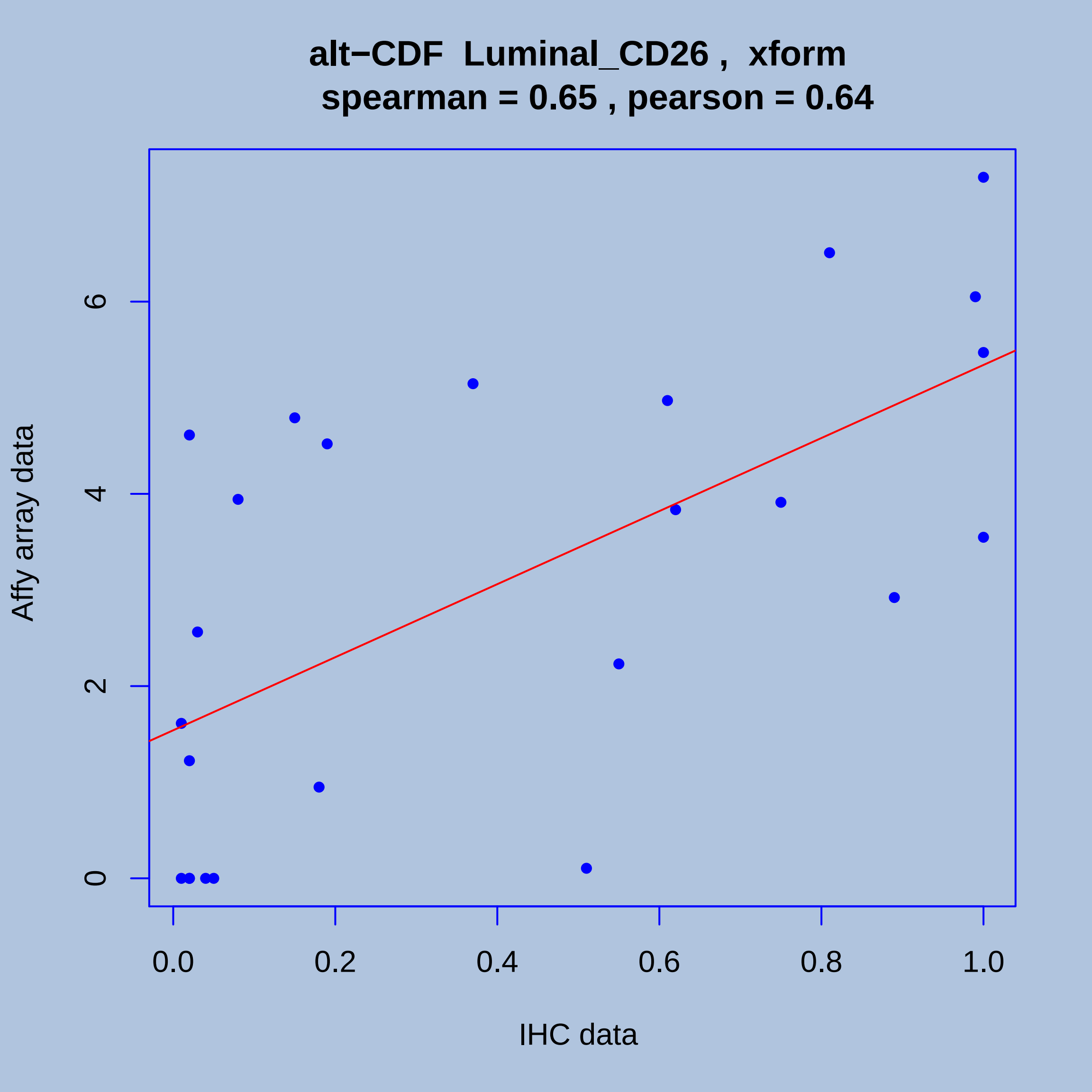
alt−CDF Luminal_CD26 , xform
spearman = 0.65 , pearson = 0.64
Affy array data
IHC data
0
2
4
6
0.0
0.2
0.4
0.6
0.8
1.0

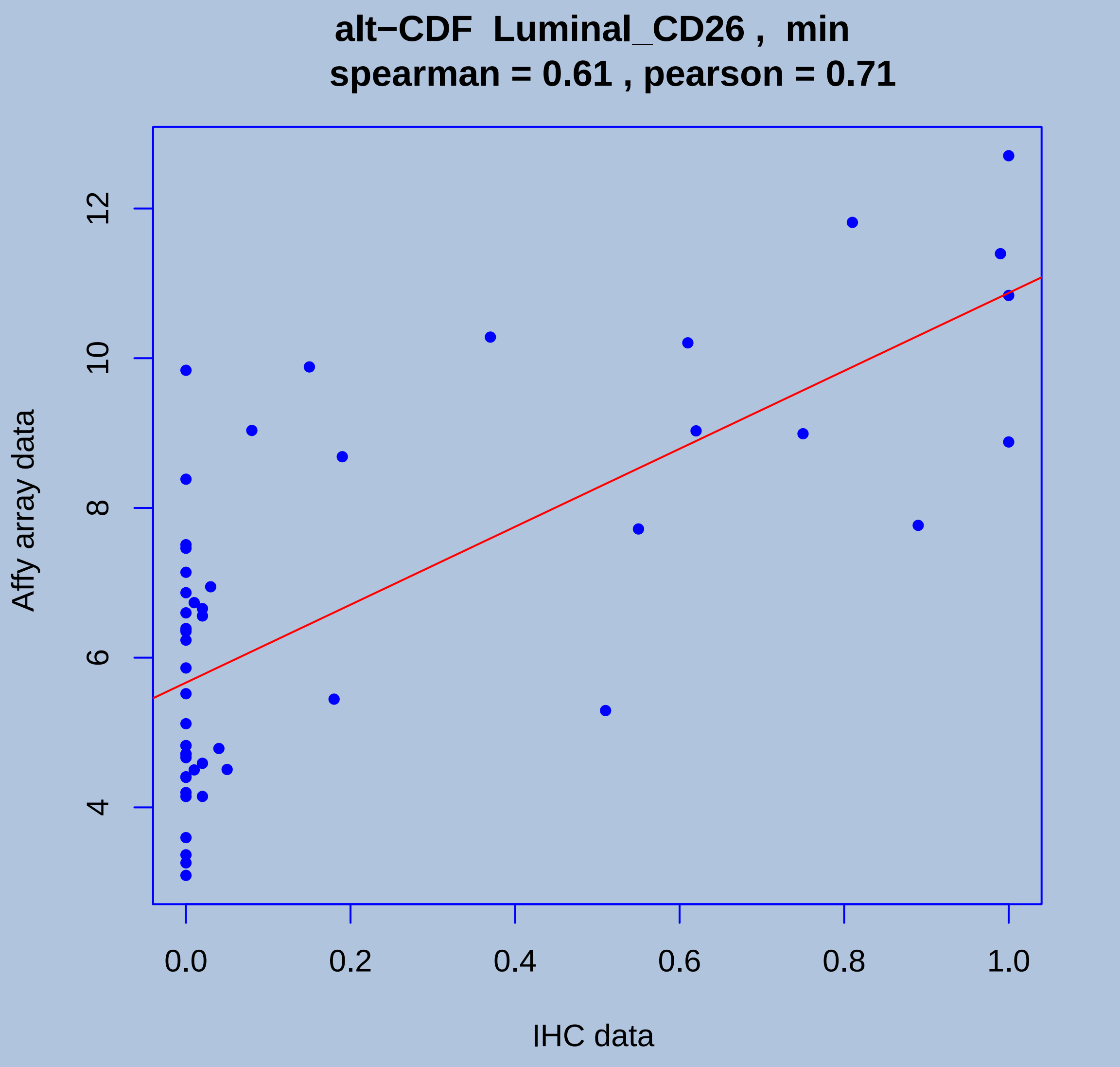

alt−CDF Luminal_CD26 , min
spearman = 0.61 , pearson = 0.71
Affy array data
4
6
8
10
12
0.0
0.2
0.4
0.6
0.8
1.0
IHC data

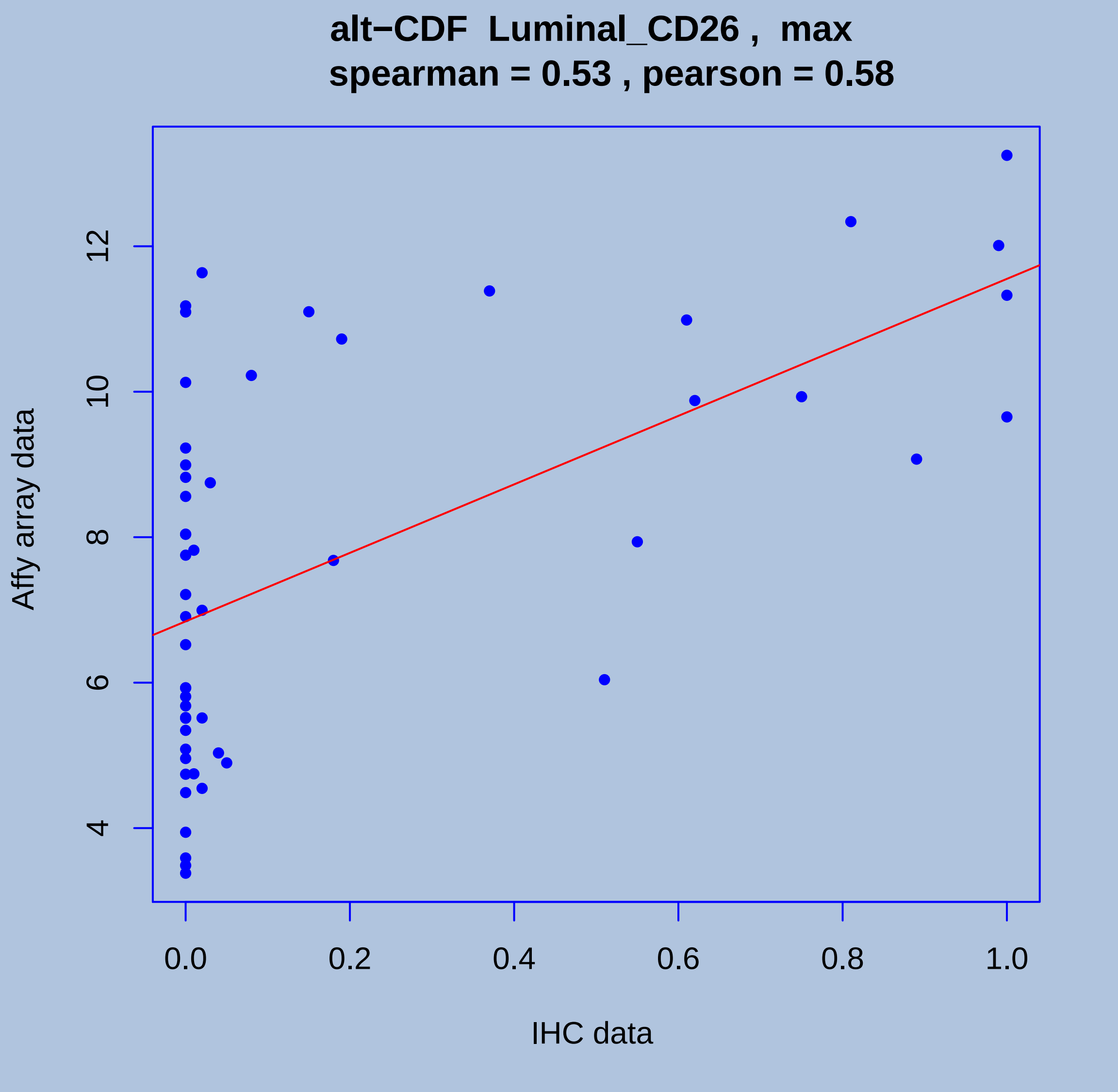

alt−CDF Luminal_CD26 , max
spearman = 0.53 , pearson = 0.58
Affy array data
IHC data
4
6
8
10
12
0.0
0.2
0.4
0.6
0.8
1.0

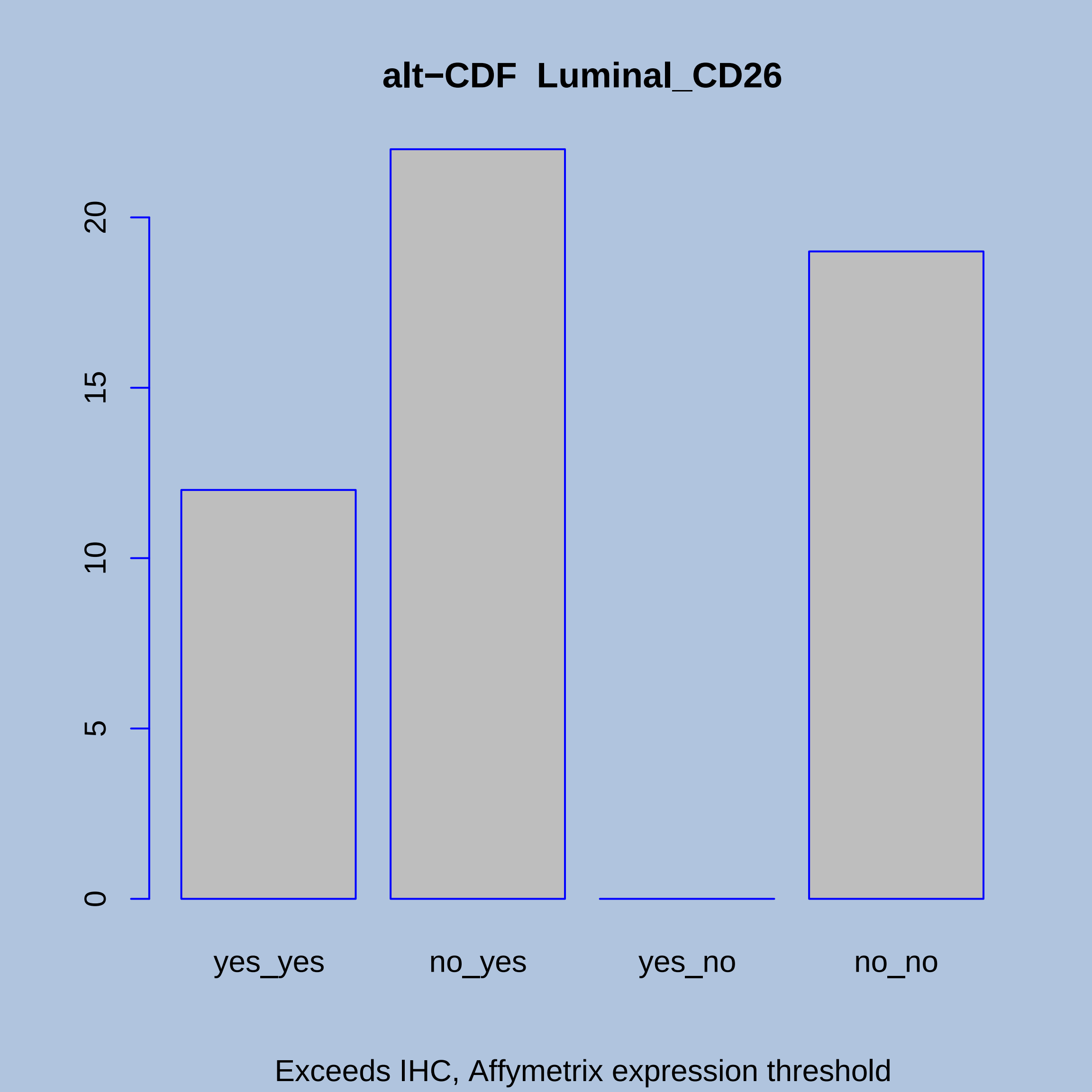

alt−CDF Luminal_CD26
0
5
10
15
20
yes_yes
no_yes
yes_no
no_no
Exceeds IHC, Affymetrix expression threshold

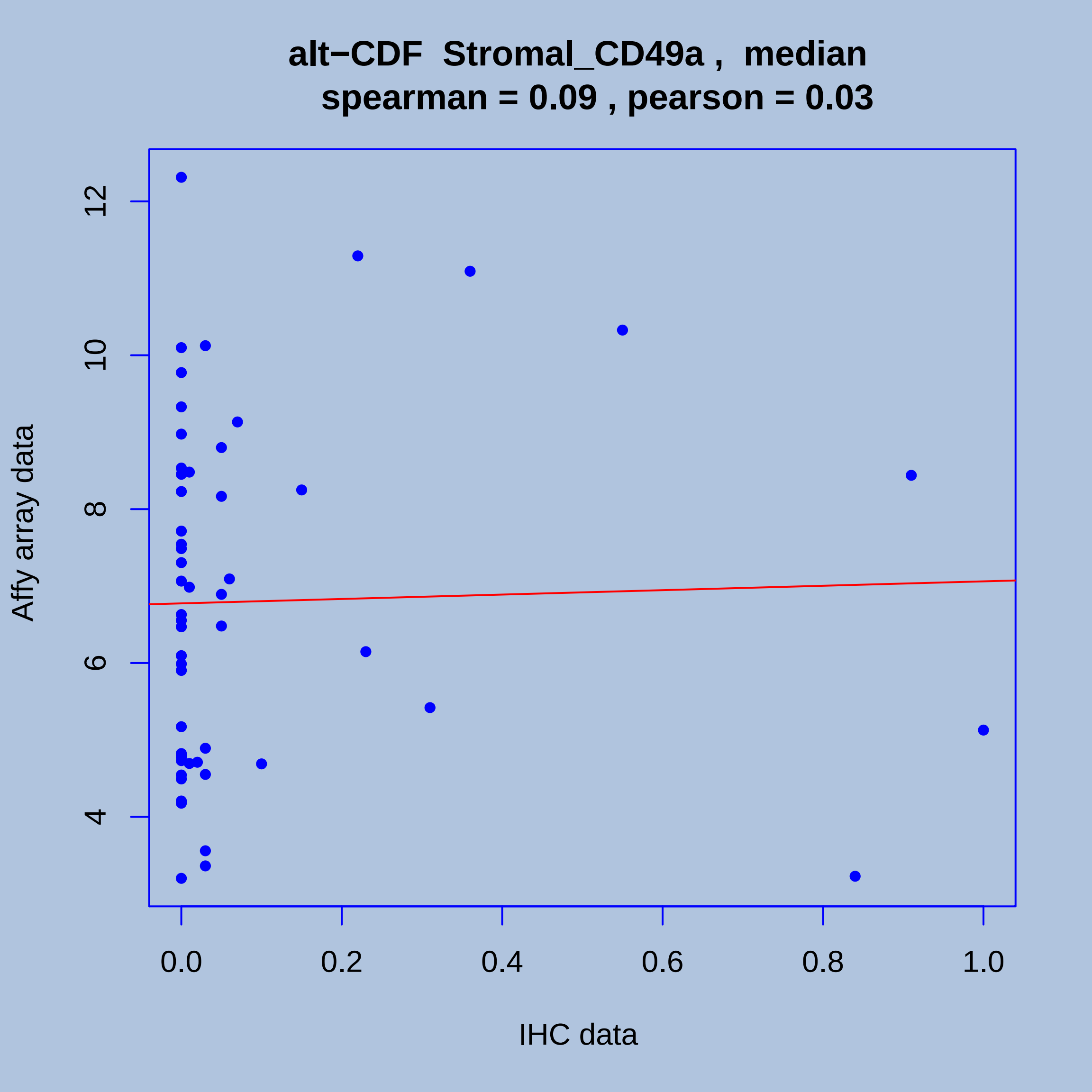

alt−CDF Stromal_CD49a , median
spearman = 0.09 , pearson = 0.03
Affy array data
IHC data
0.0
0.2
0.4
0.6
0.8
1.0
4
6
8
10
12

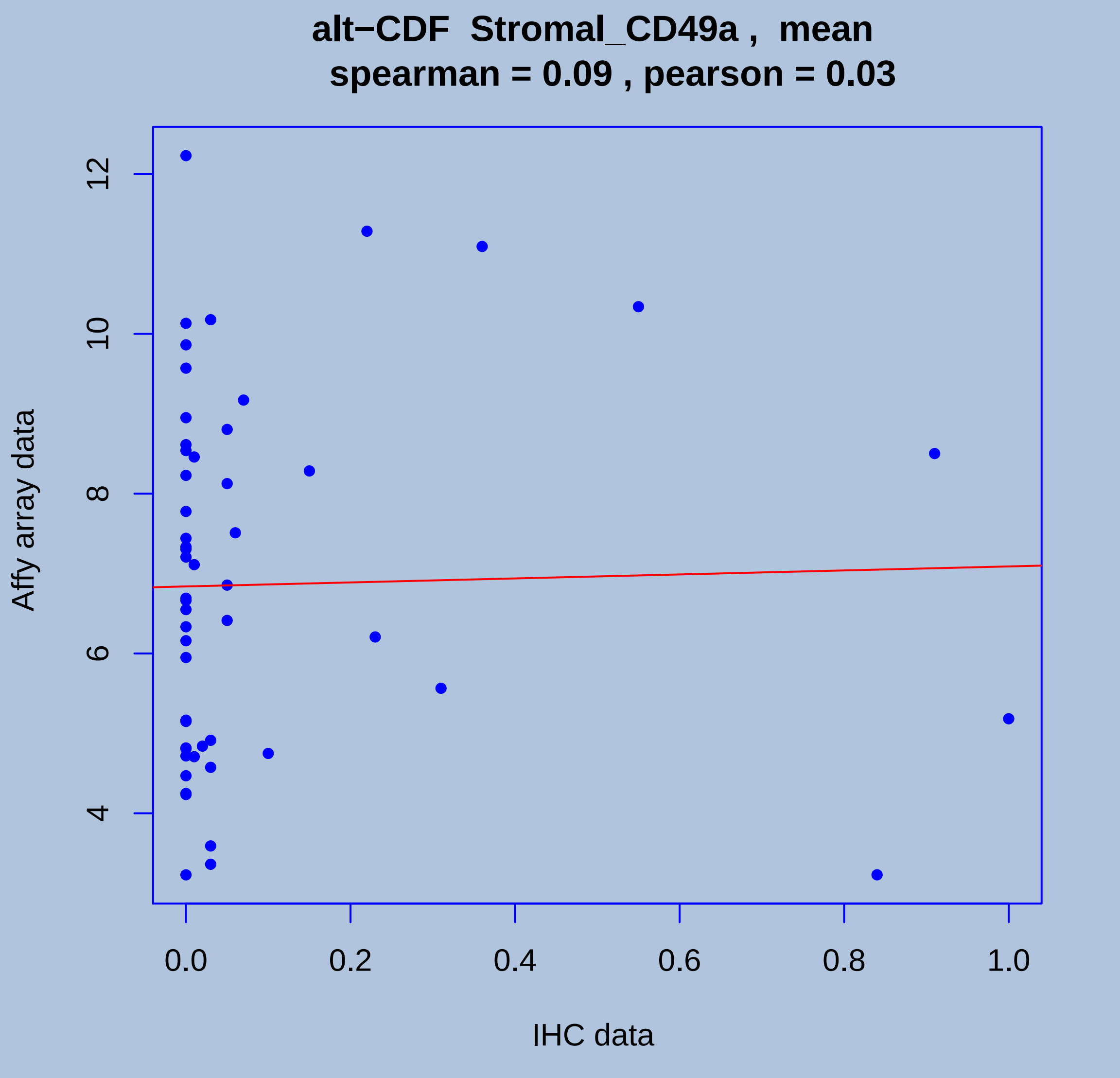

alt−CDF Stromal_CD49a , mean
spearman = 0.09 , pearson = 0.03
Affy array data
IHC data
12
10
8
6
4
0.0
0.2
0.4
0.6
0.8
1.0

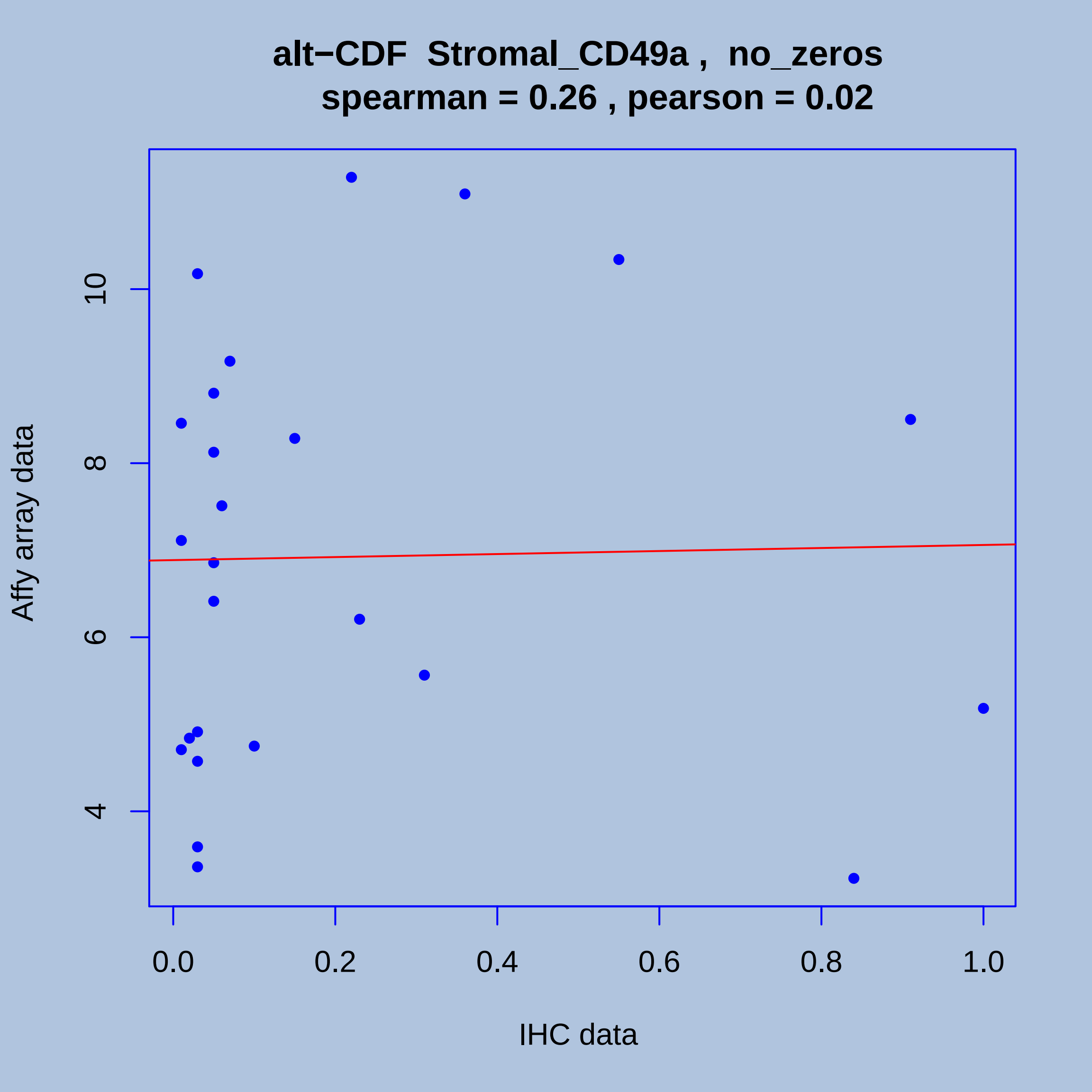

alt−CDF Stromal_CD49a , no_zeros
spearman = 0.26 , pearson = 0.02
Affy array data
IHC data
4
6
8
10
0.0
0.2
0.4
0.6
0.8
1.0

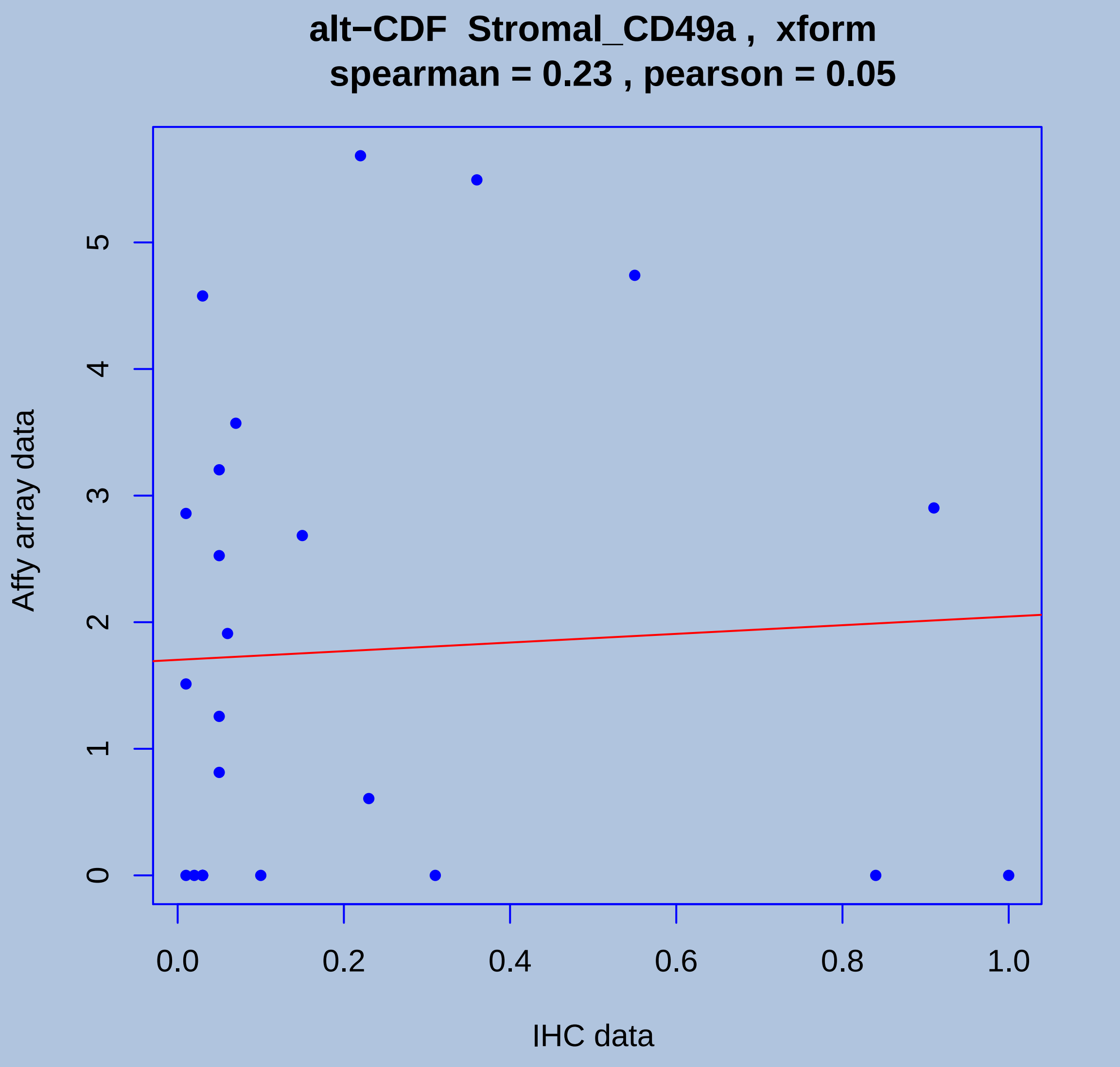
alt−CDF Stromal_CD49a , xform
spearman = 0.23 , pearson = 0.05
Affy array data
IHC data
0
1
2
3
4
5
0.0
0.2
0.4
0.6
0.8
1.0

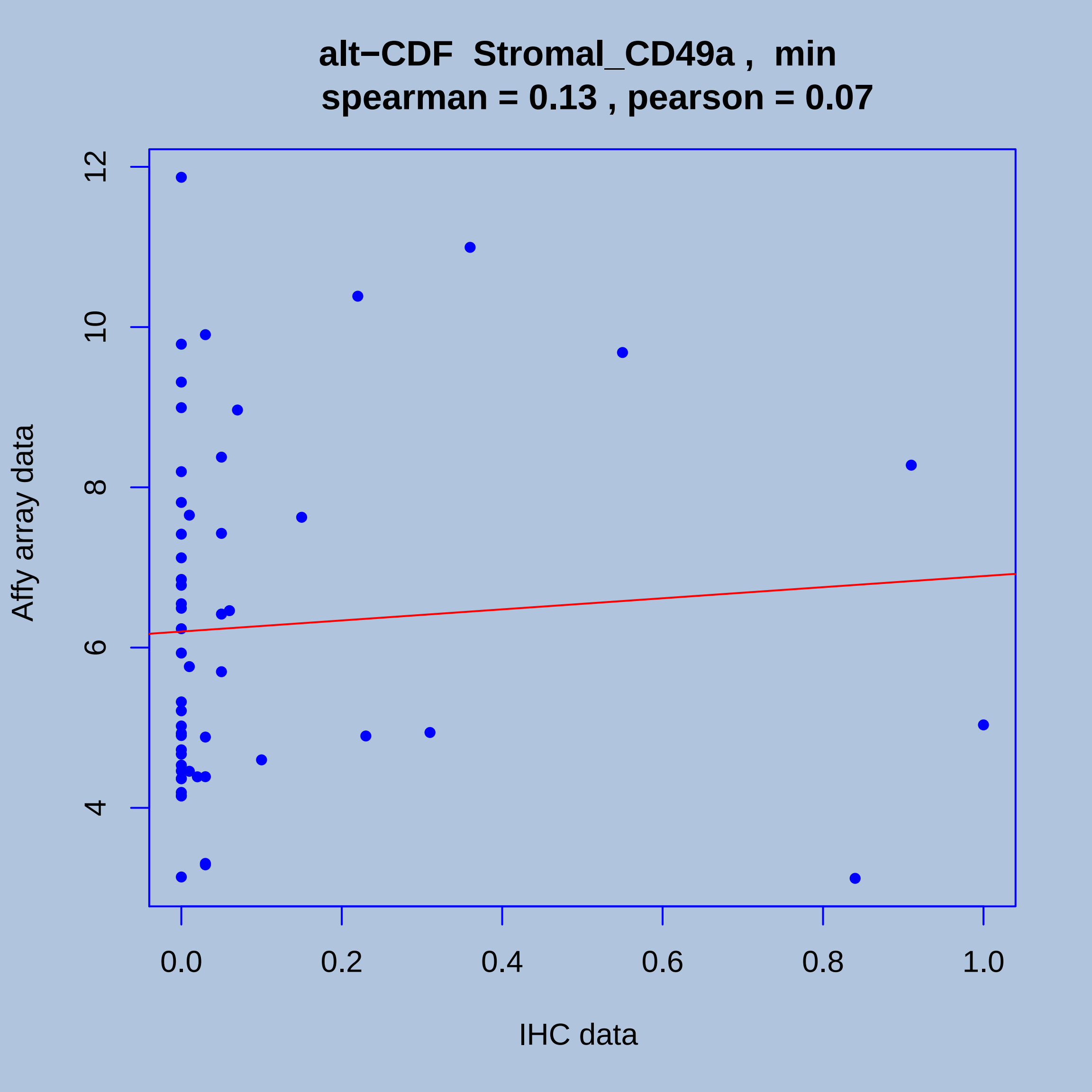

alt−CDF Stromal_CD49a , min
spearman = 0.13 , pearson = 0.07
Affy array data
IHC data
0.0
0.2
0.4
0.6
0.8
1.0
4
6
8
10
12

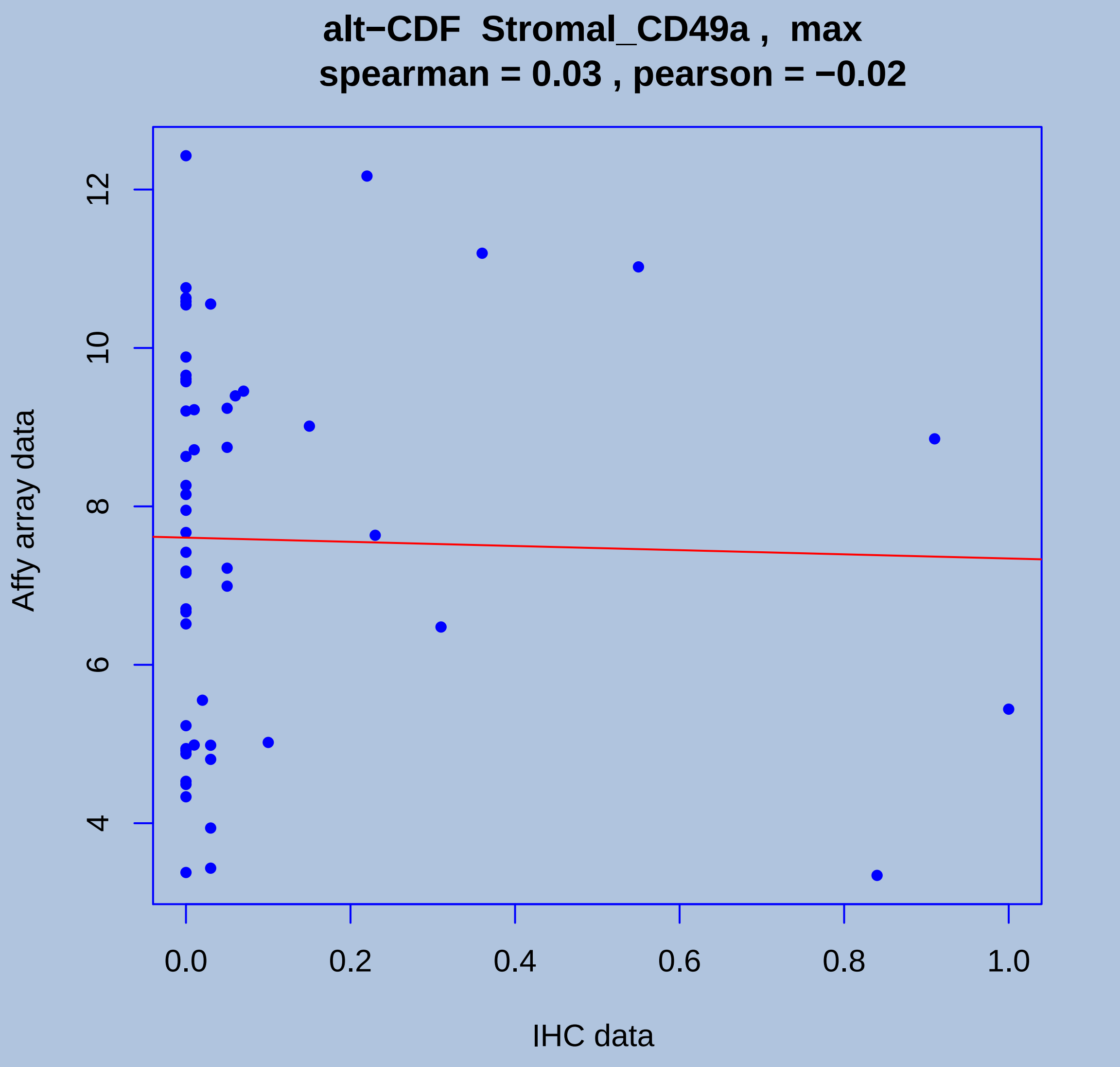

alt−CDF Stromal_CD49a , max
spearman = 0.03 , pearson = −0.02
Affy array data
IHC data
4
6
8
10
12
0.0
0.2
0.4
0.6
0.8
1.0

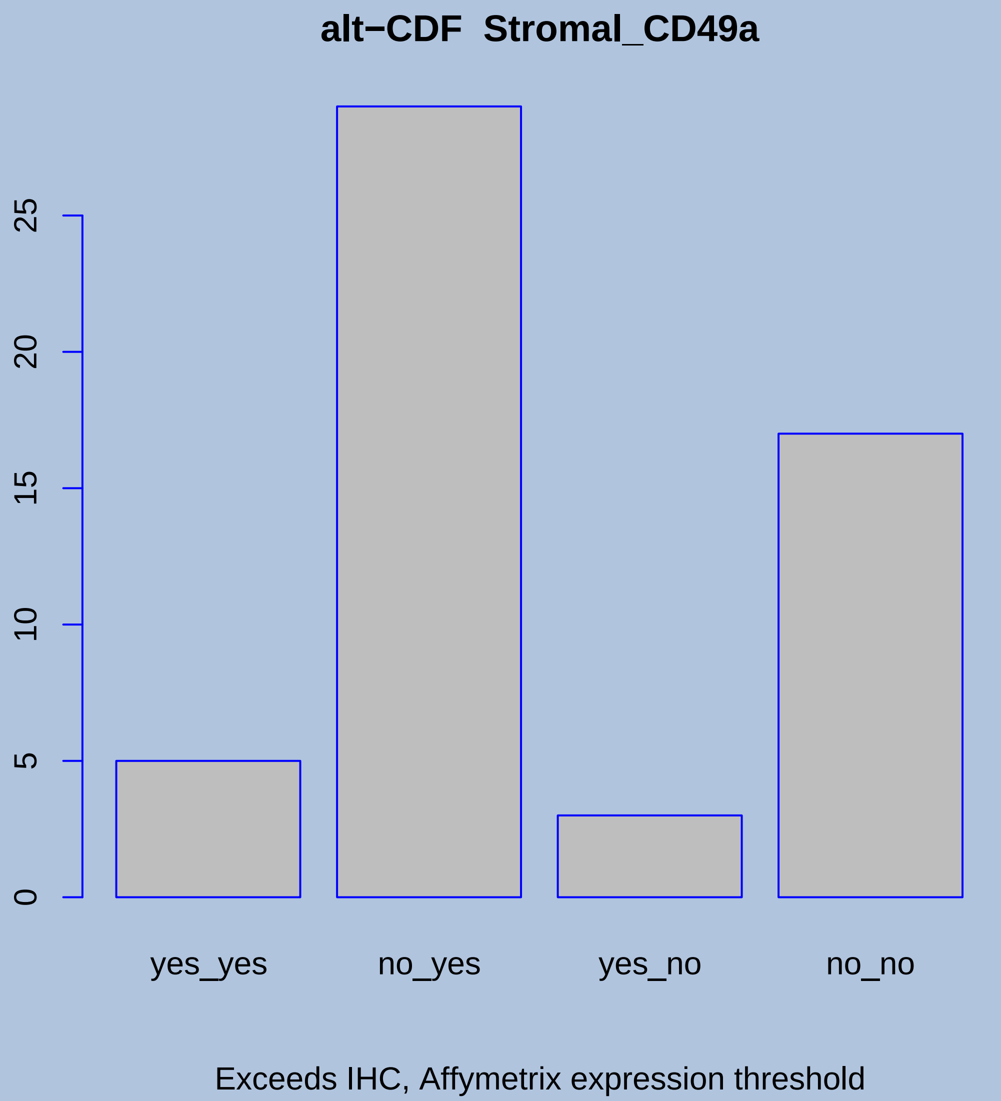

alt−CDF Stromal_CD49a
0
5
10
15
20
25
yes_yes
no_yes
yes_no
no_no
Exceeds IHC, Affymetrix expression threshold

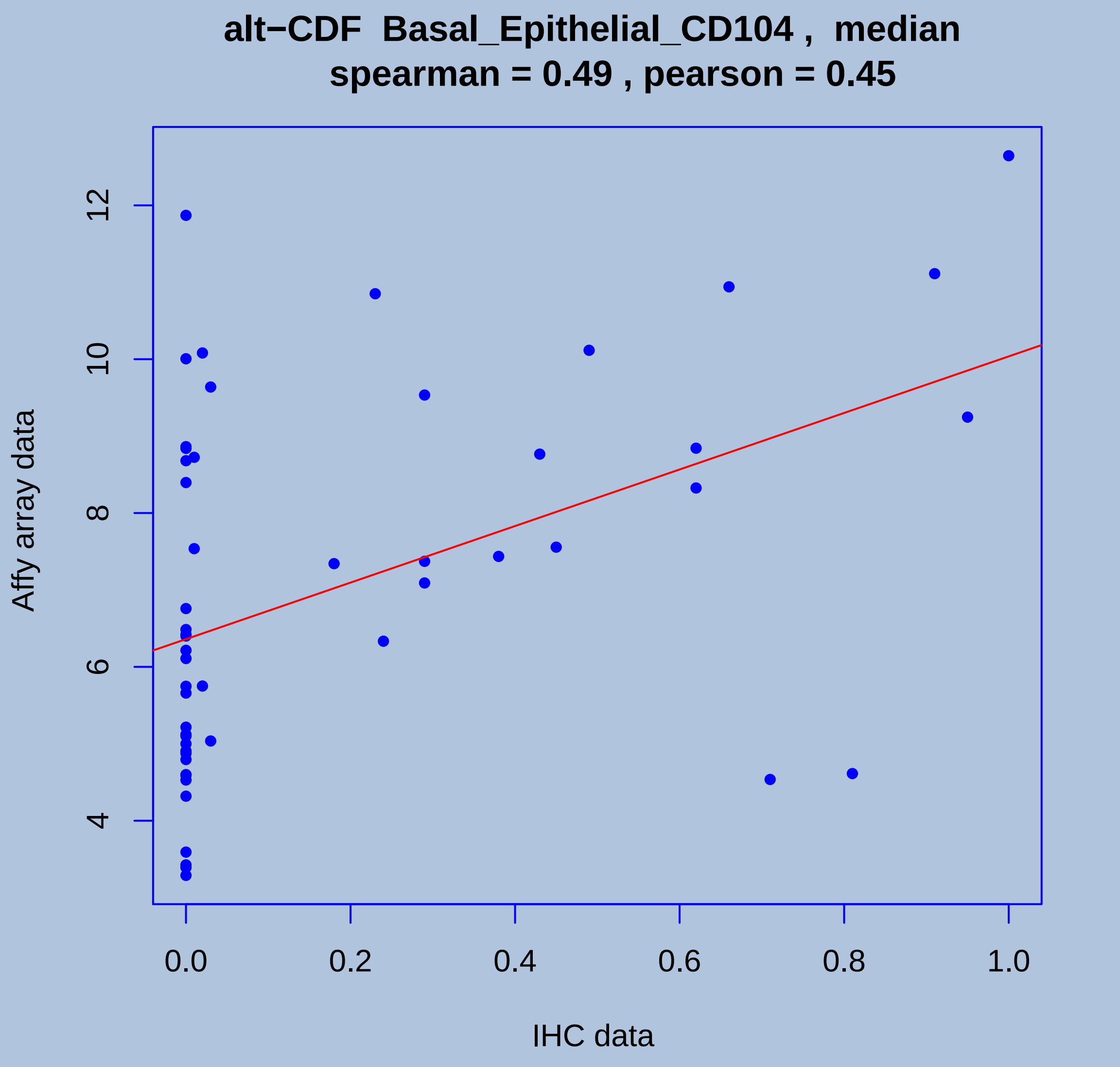
alt−CDF Basal_Epithelial_CD104 , median
spearman = 0.49 , pearson = 0.45
Affy array data
IHC data
0.0
0.2
0.4
0.6
0.8
1.0
4
6
8
10
12

alt−CDF Basal_Epithelial_CD104 , mean
spearman = 0.49 , pearson = 0.45

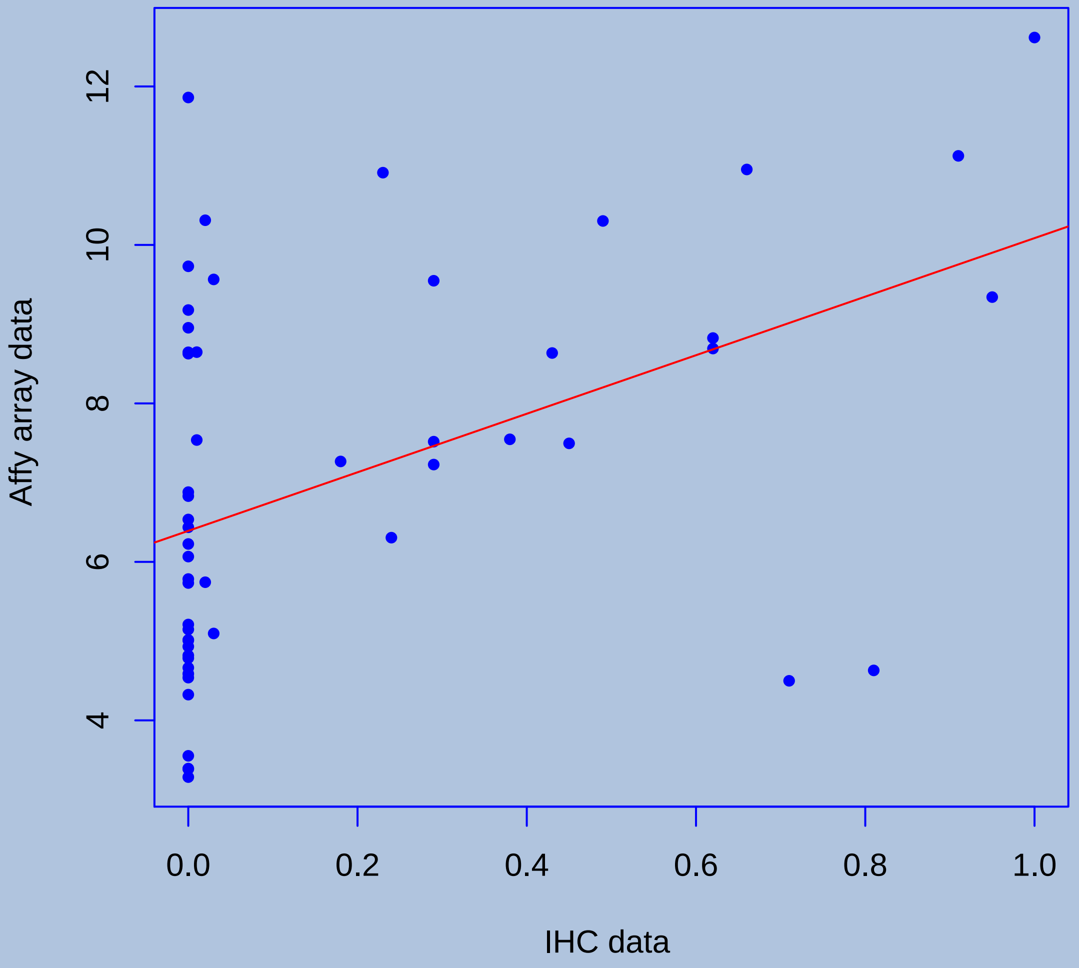

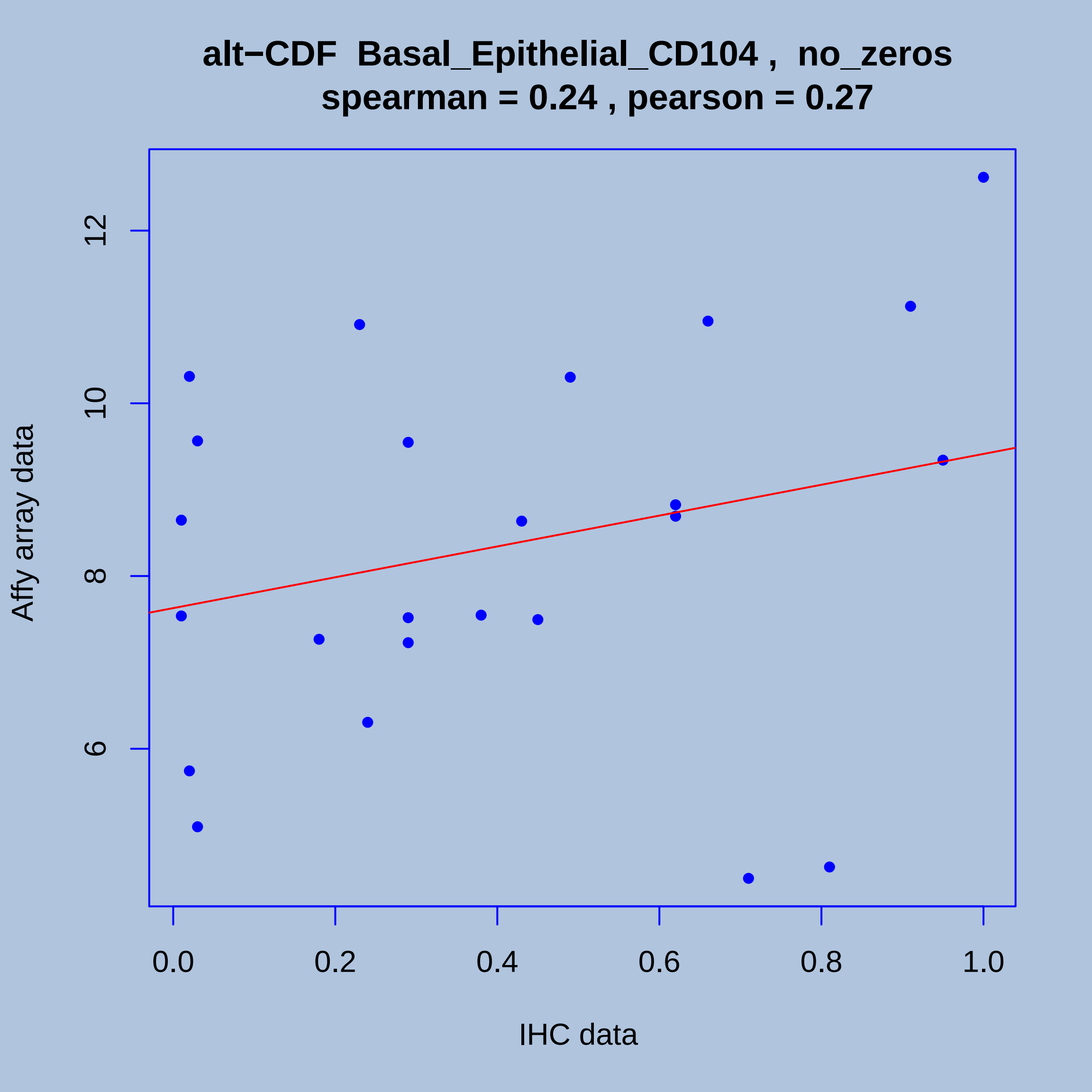

alt−CDF Basal_Epithelial_CD104 , no_zeros
spearman = 0.24 , pearson = 0.27
Affy array data
IHC data
6
8
10
12
0.0
0.2
0.4
0.6
0.8
1.0

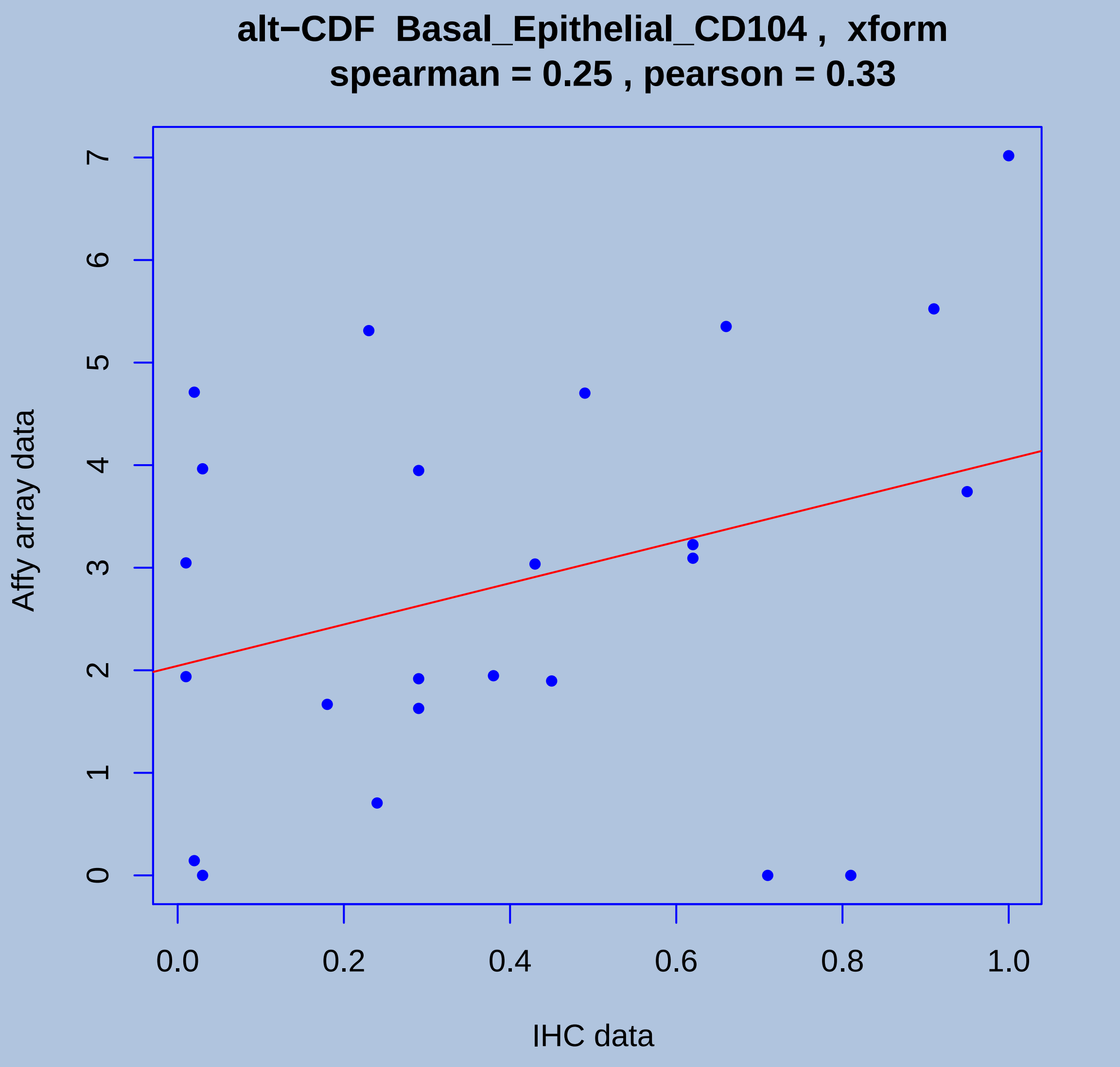

alt−CDF Basal_Epithelial_CD104 , xform
spearman = 0.25 , pearson = 0.33
Affy array data
0
1
2
3
4
5
6
7
0.0
0.2
0.4
0.6
0.8
1.0
IHC data

alt−CDF Basal_Epithelial_CD104 , min
spearman = 0.49 , pearson = 0.46

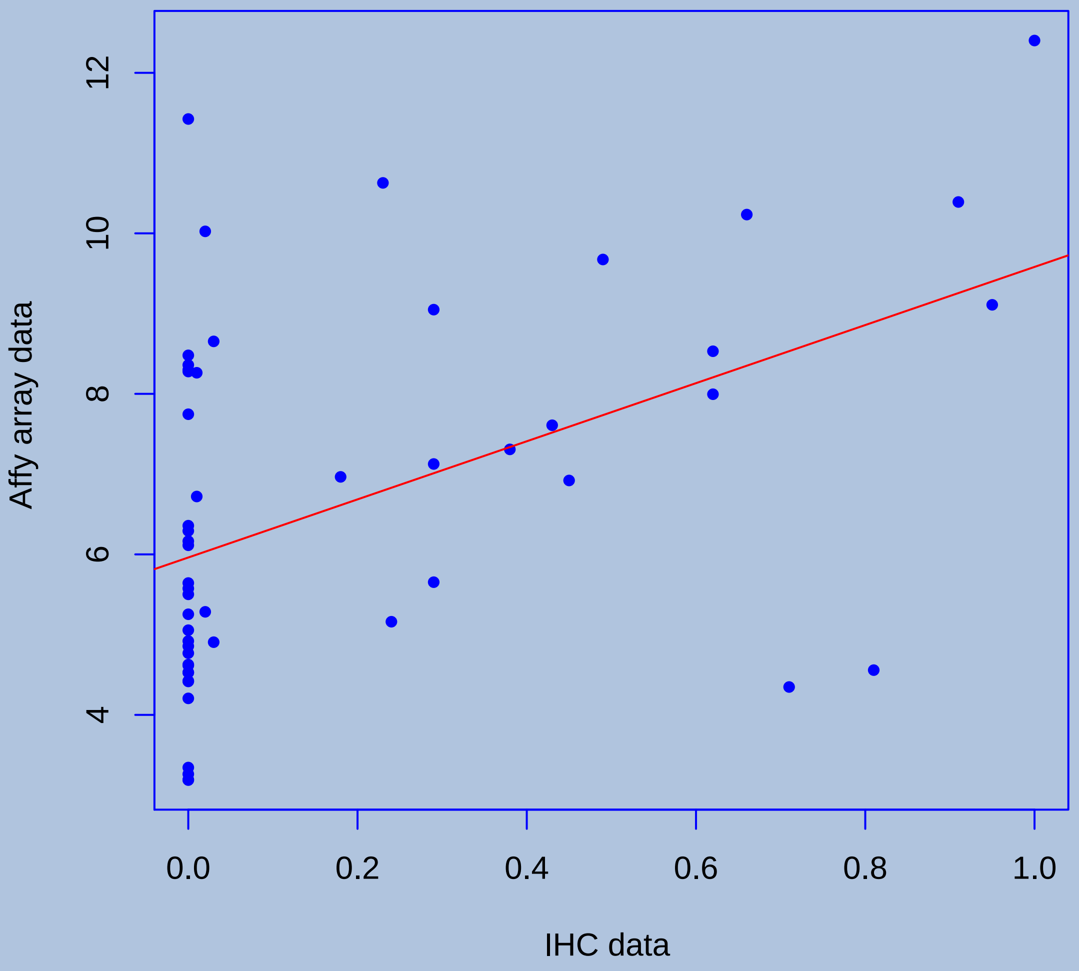

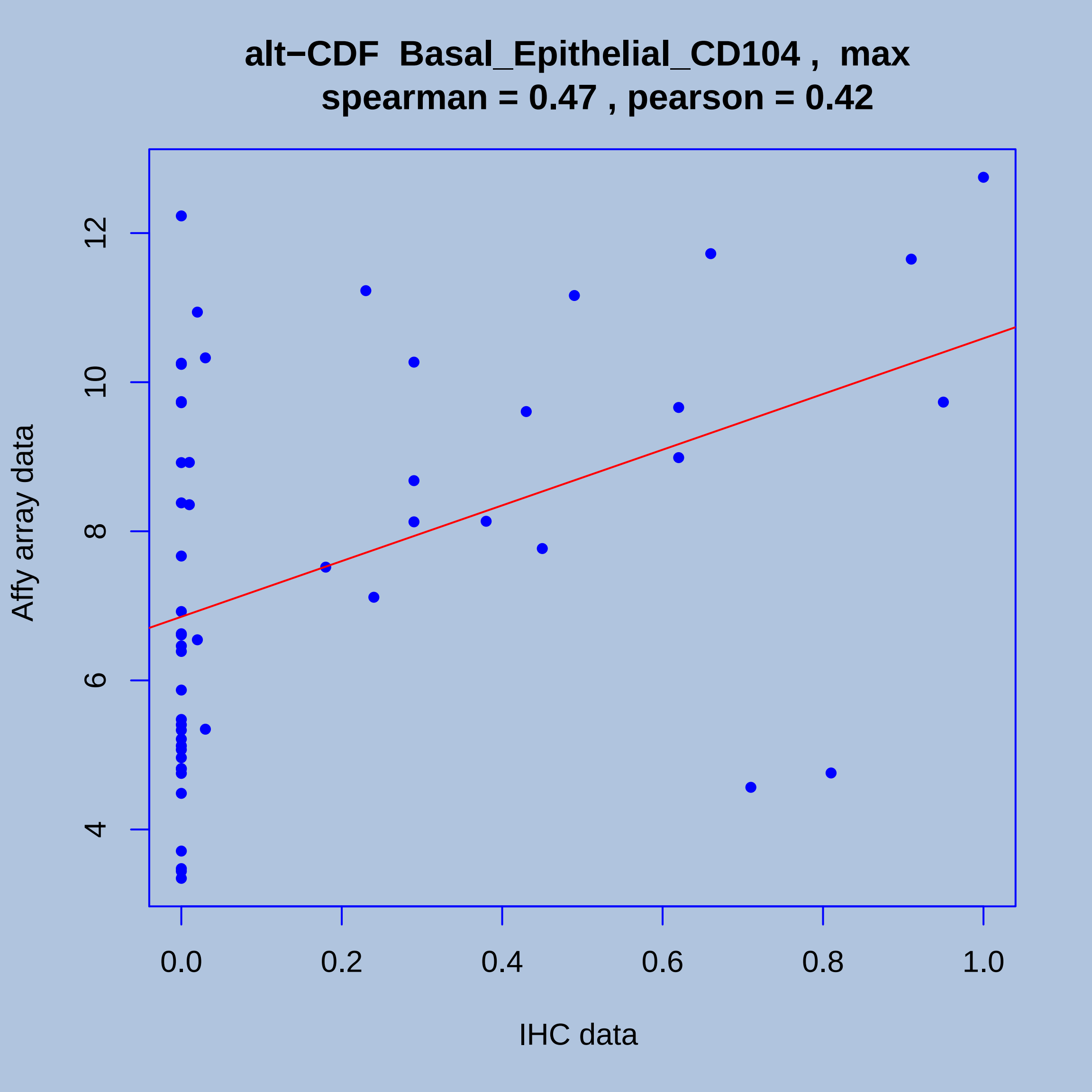

alt−CDF Basal_Epithelial_CD104 , max
spearman = 0.47 , pearson = 0.42
Affy array data
4
6
8
10
12
0.0
0.2
0.4
0.6
0.8
1.0
IHC data

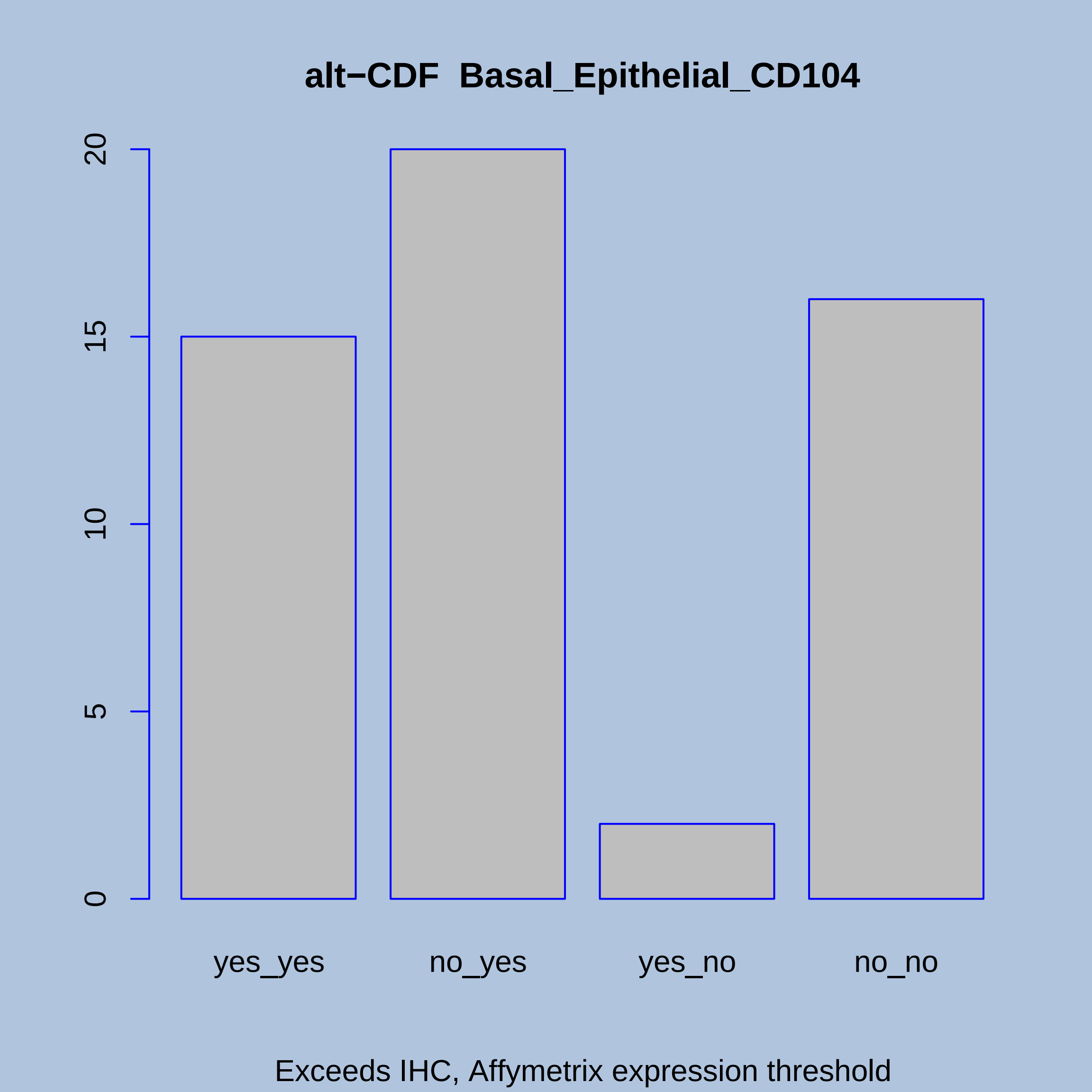
alt−CDF Basal_Epithelial_CD104
0
5
10
15
20
yes_yes
no_yes
yes_no
no_no
Exceeds IHC, Affymetrix expression threshold

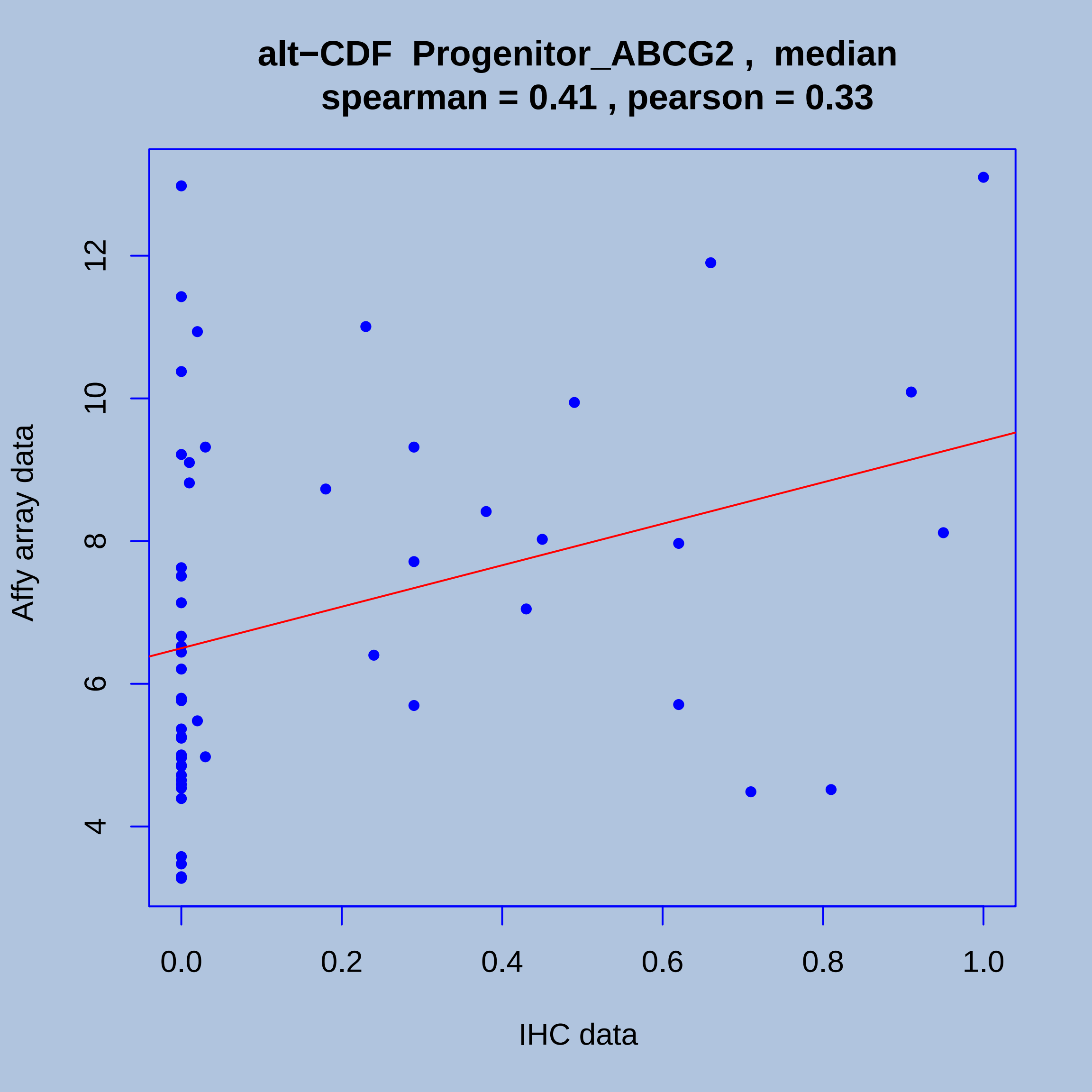
alt−CDF Progenitor_ABCG2 , median
spearman = 0.41 , pearson = 0.33
Affy array data
IHC data
4
6
8
10
12
0.0
0.2
0.4
0.6
0.8
1.0

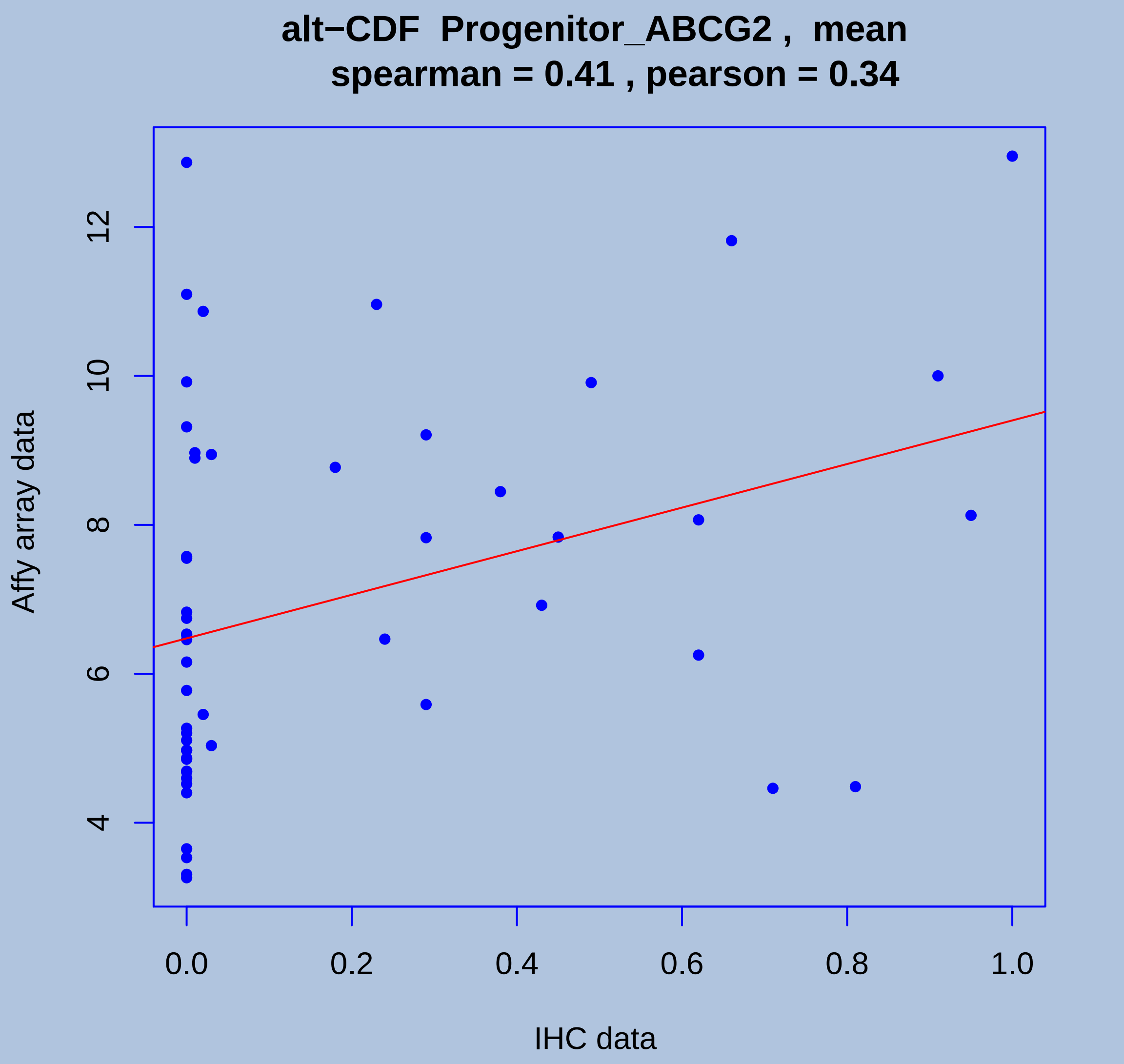

alt−CDF Progenitor_ABCG2 , mean
spearman = 0.41 , pearson = 0.34
Affy array data
IHC data
4
6
8
10
12
0.0
0.2
0.4
0.6
0.8
1.0

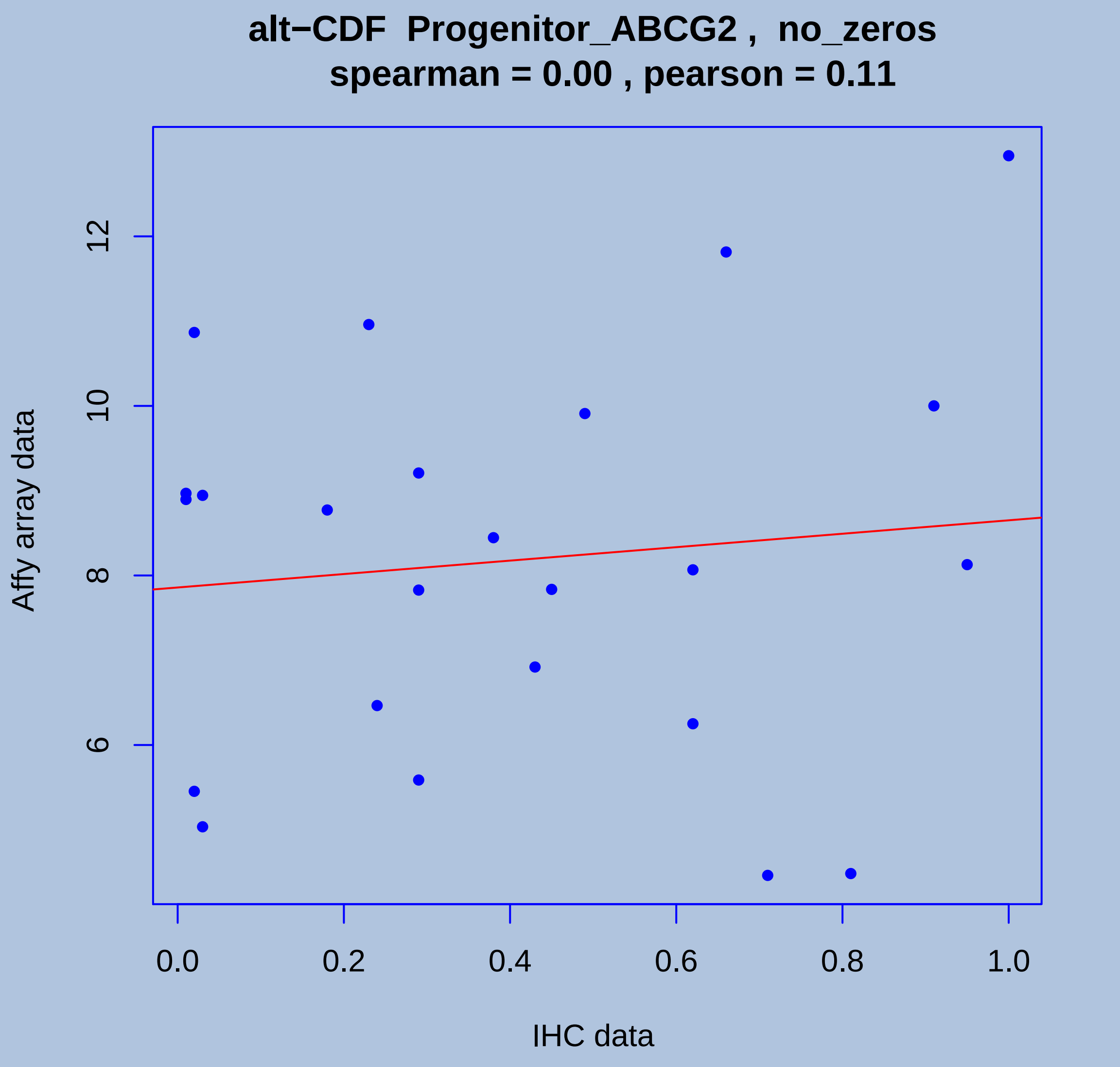

alt−CDF Progenitor_ABCG2 , no_zeros
spearman = 0.00 , pearson = 0.11
Affy array data
12
10
8
6
0.0
0.2
0.4
0.6
0.8
1.0
IHC data

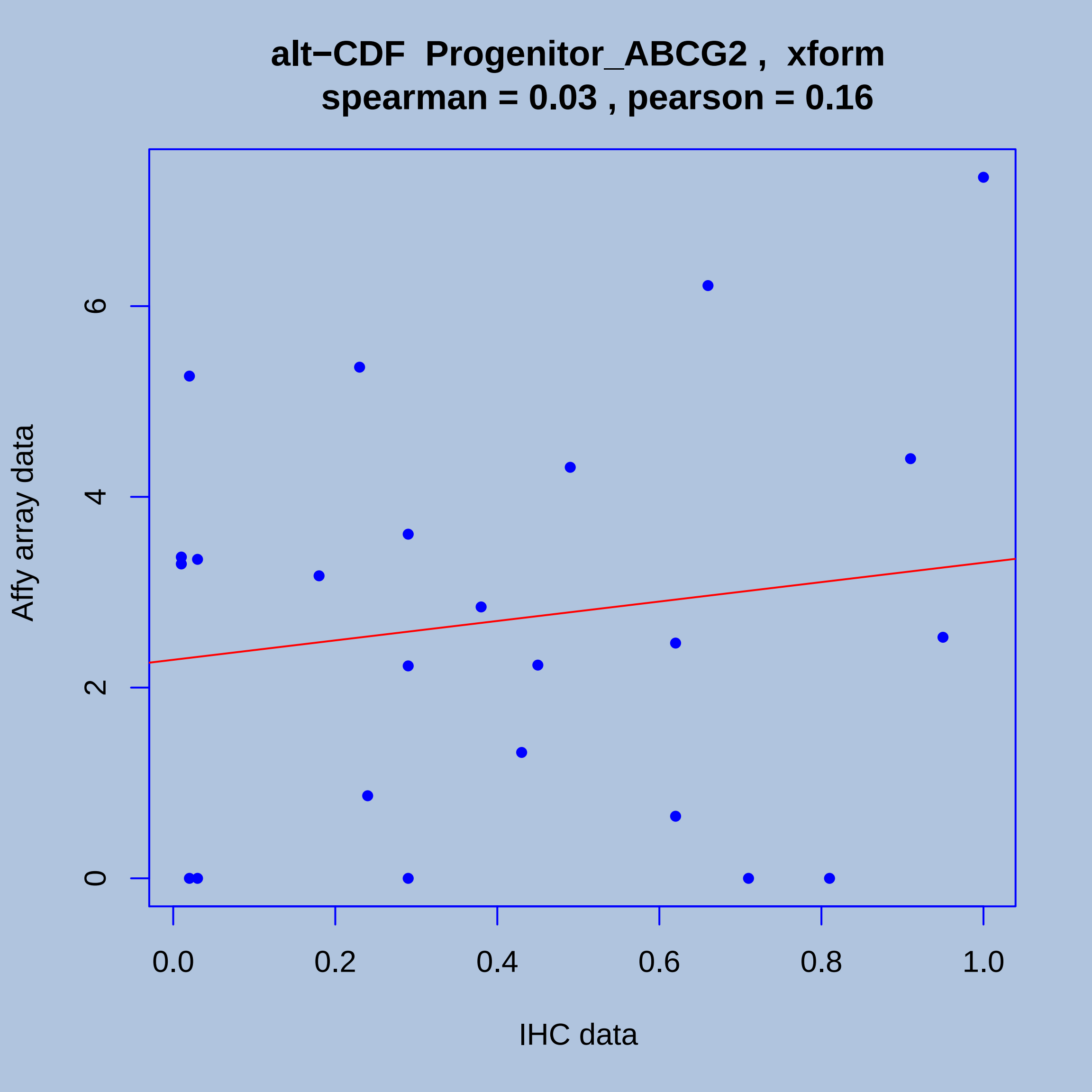
alt−CDF Progenitor_ABCG2 , xform
spearman = 0.03 , pearson = 0.16
Affy array data
IHC data
0
2
4
6
0.0
0.2
0.4
0.6
0.8
1.0

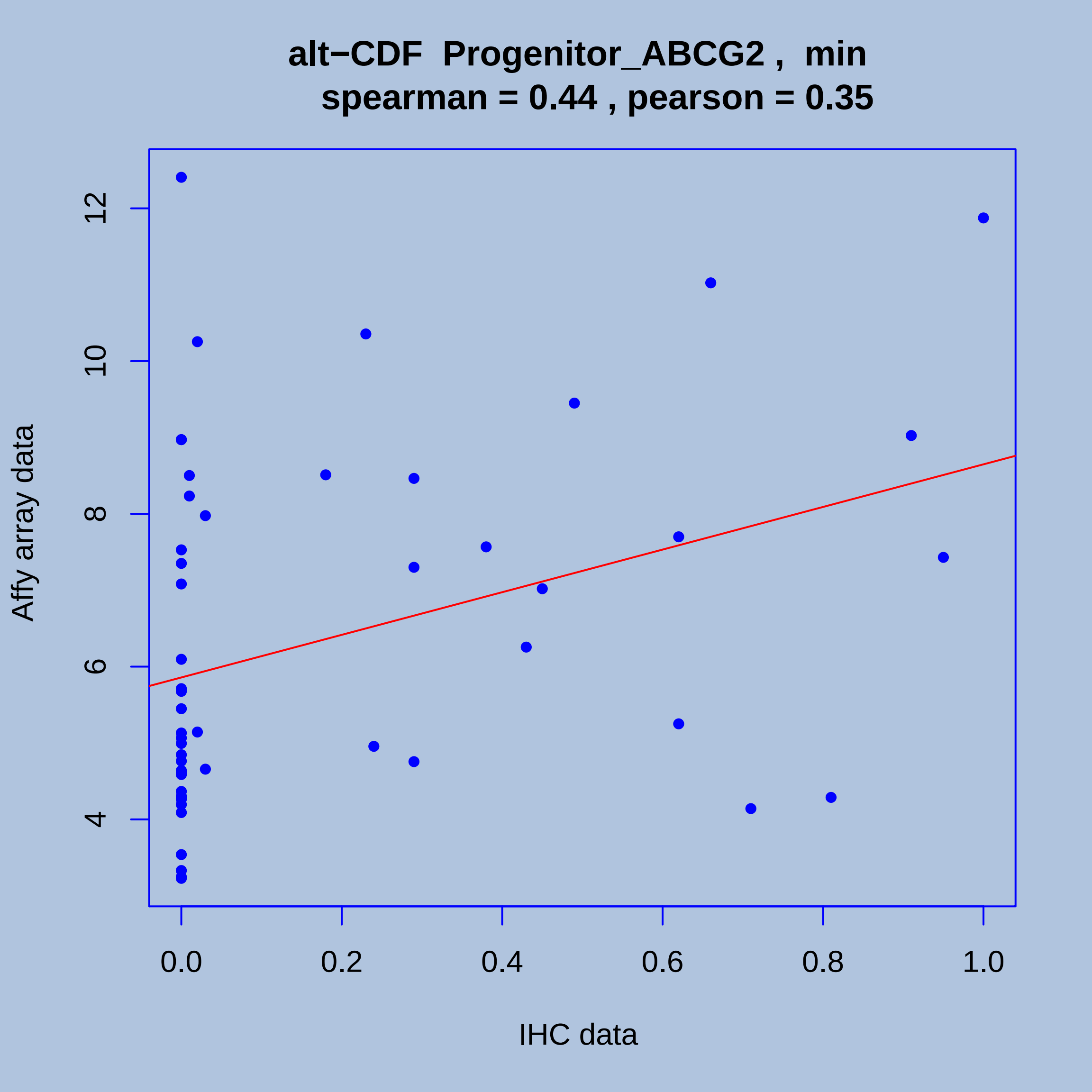
alt−CDF Progenitor_ABCG2 , min
spearman = 0.44 , pearson = 0.35
Affy array data
4
6
8
10
12
0.0
0.2
0.4
0.6
0.8
1.0
IHC data

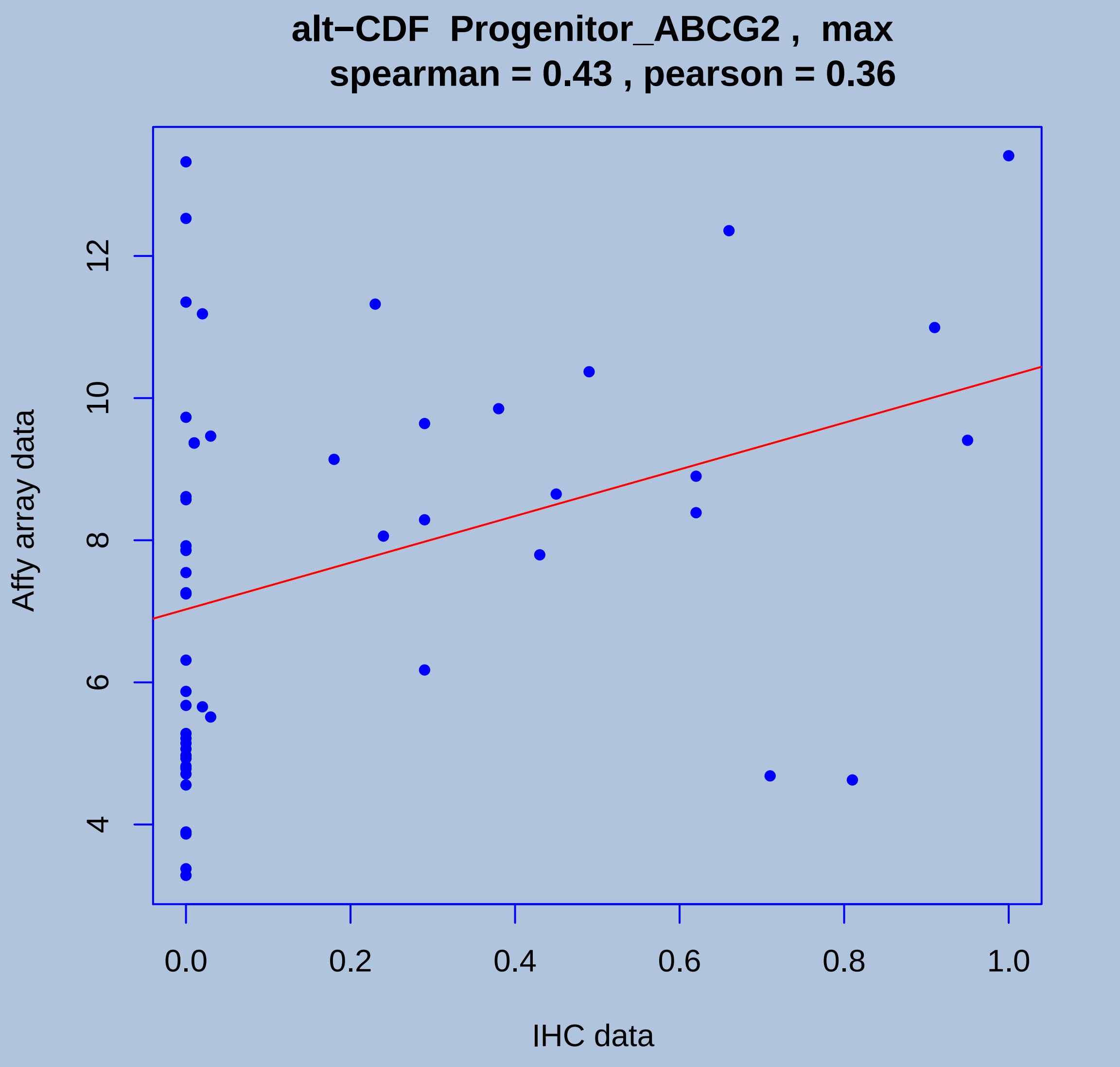
alt−CDF Progenitor_ABCG2 , max
spearman = 0.43 , pearson = 0.36
Affy array data
IHC data
4
6
8
10
12
0.0
0.2
0.4
0.6
0.8
1.0

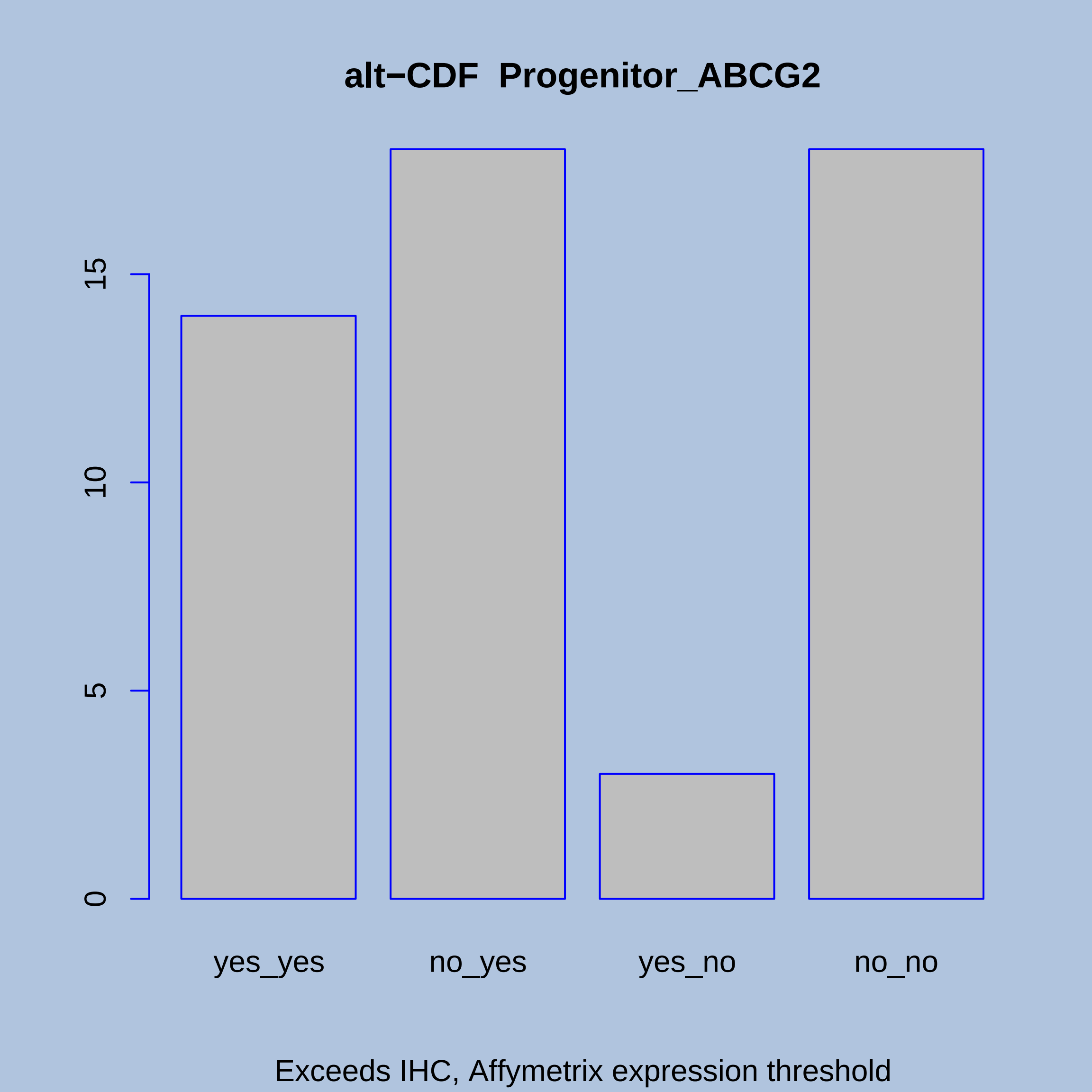
alt−CDF Progenitor_ABCG2
0
5
10
15
yes_yes
no_yes
yes_no
no_no
Exceeds IHC, Affymetrix expression threshold

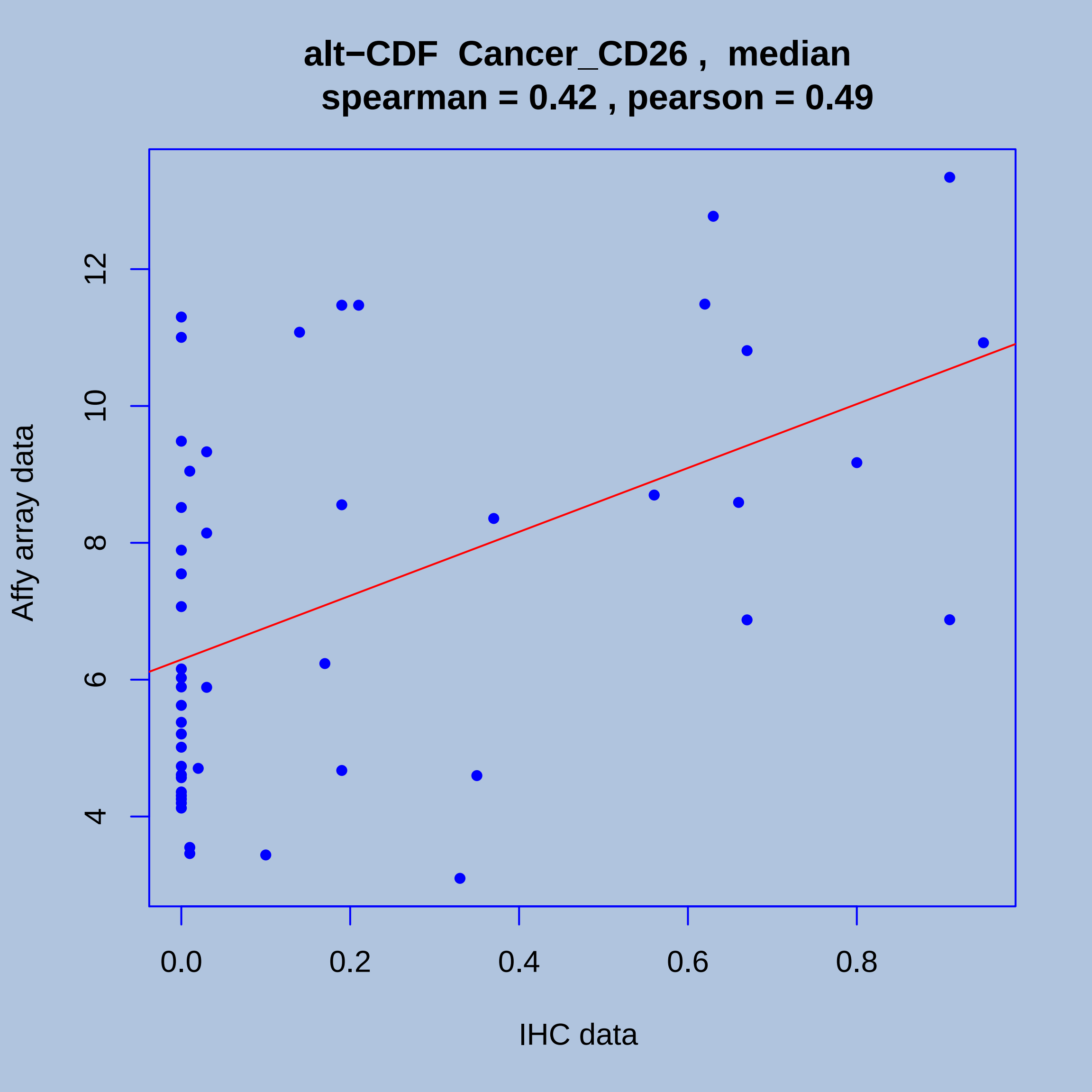
alt−CDF Cancer_CD26 , median
spearman = 0.42 , pearson = 0.49
Affy array data
IHC data
4
6
8
10
12
0.0
0.2
0.4
0.6
0.8

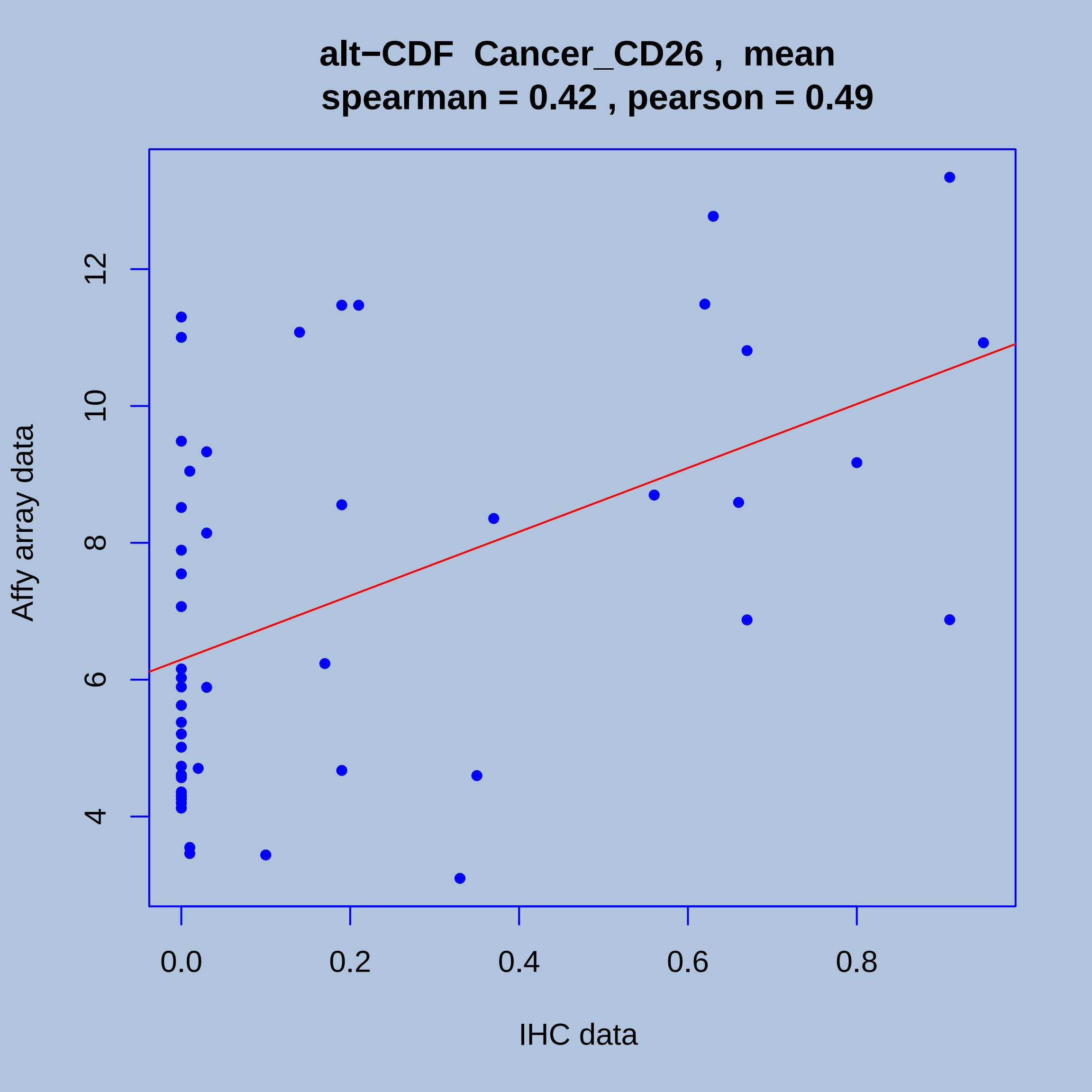
alt−CDF Cancer_CD26 , mean
spearman = 0.42 , pearson = 0.49
Affy array data
4
6
8
10
12
IHC data
0.0
0.2
0.4
0.6
0.8

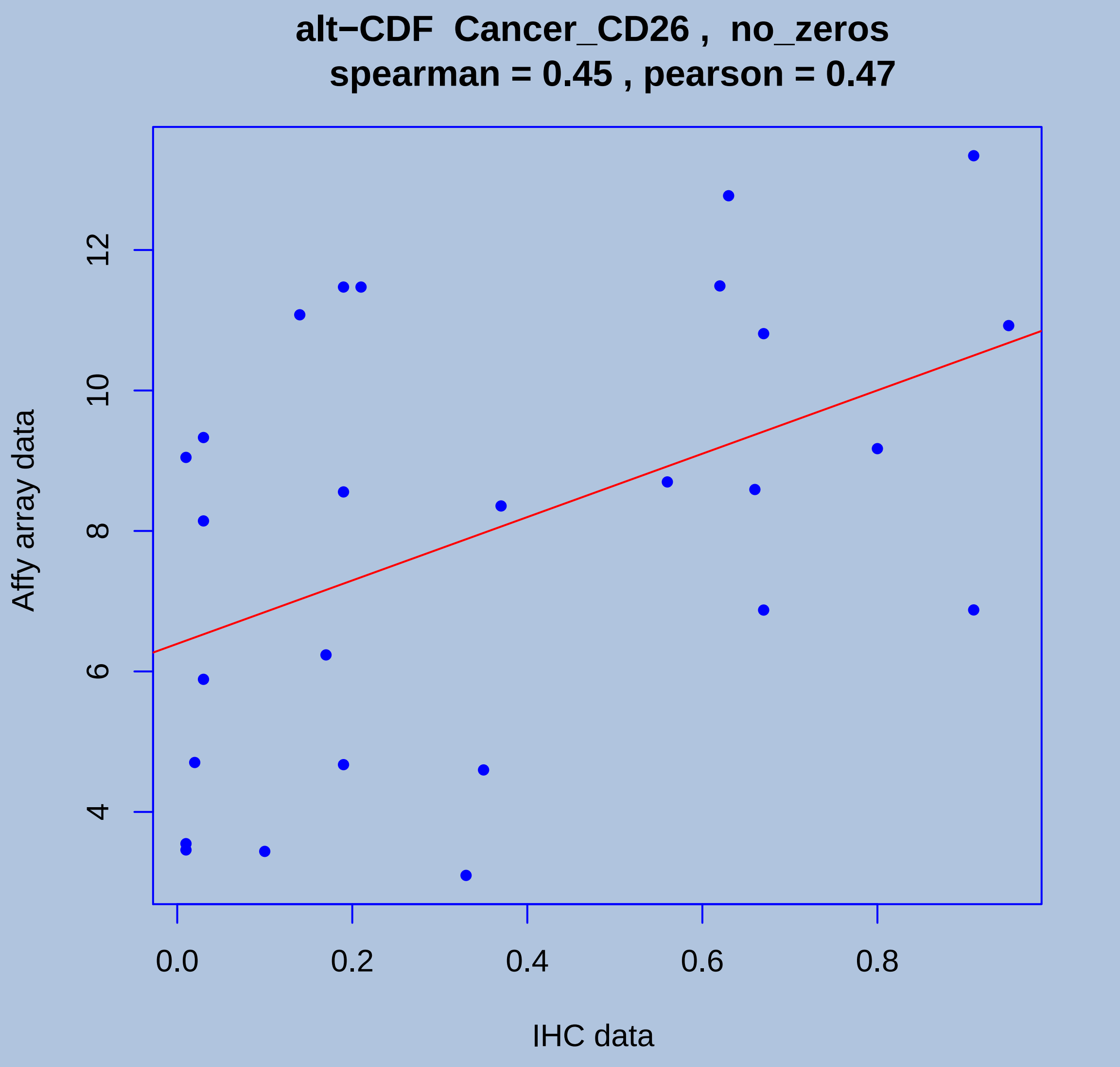

alt−CDF Cancer_CD26 , no_zeros
spearman = 0.45 , pearson = 0.47
Affy array data
IHC data
4
6
8
10
12
0.0
0.2
0.4
0.6
0.8

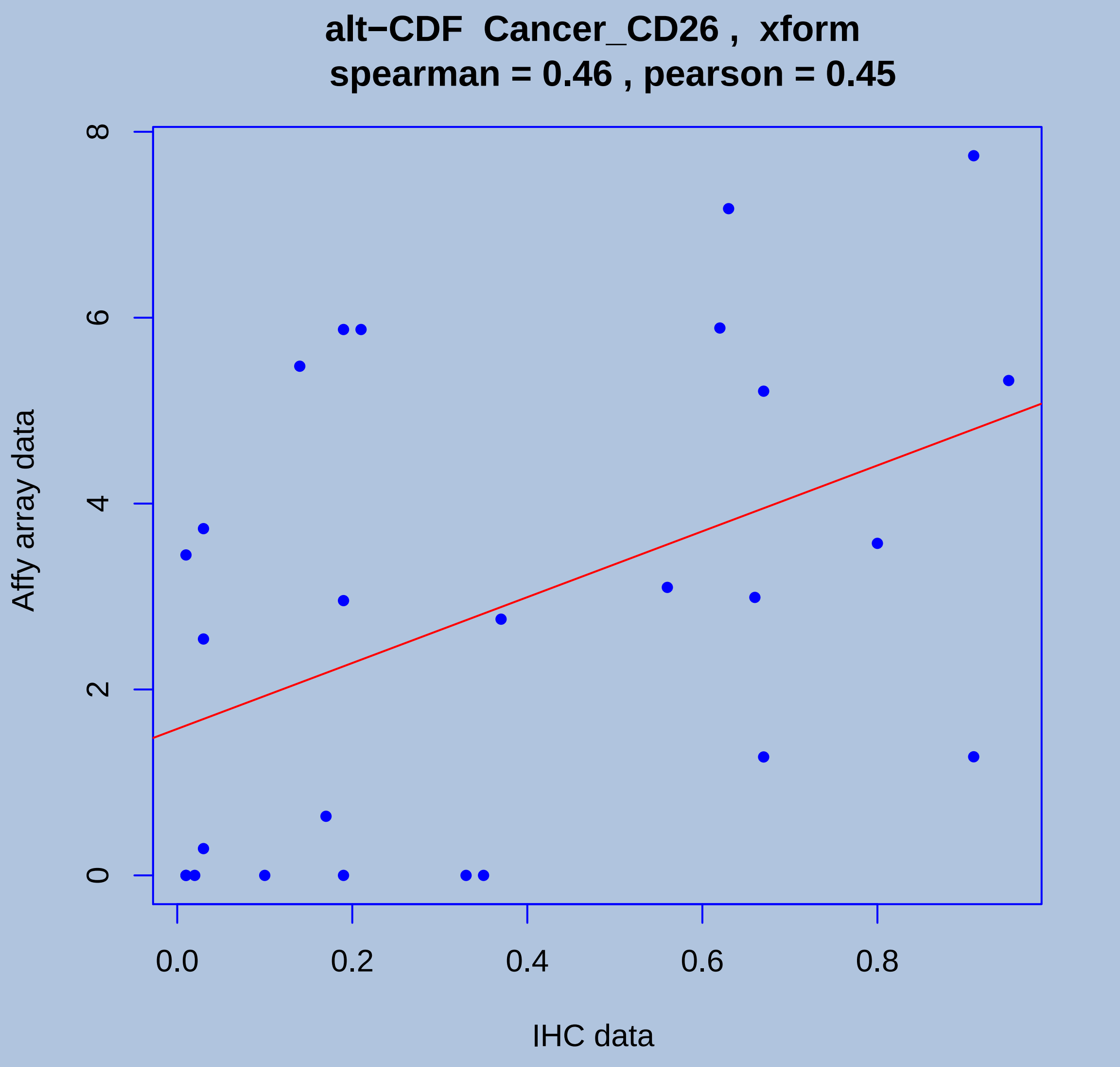
alt−CDF Cancer_CD26 , xform
spearman = 0.46 , pearson = 0.45
Affy array data
0
2
4
6
8
0.0
0.2
0.4
0.6
0.8
IHC data

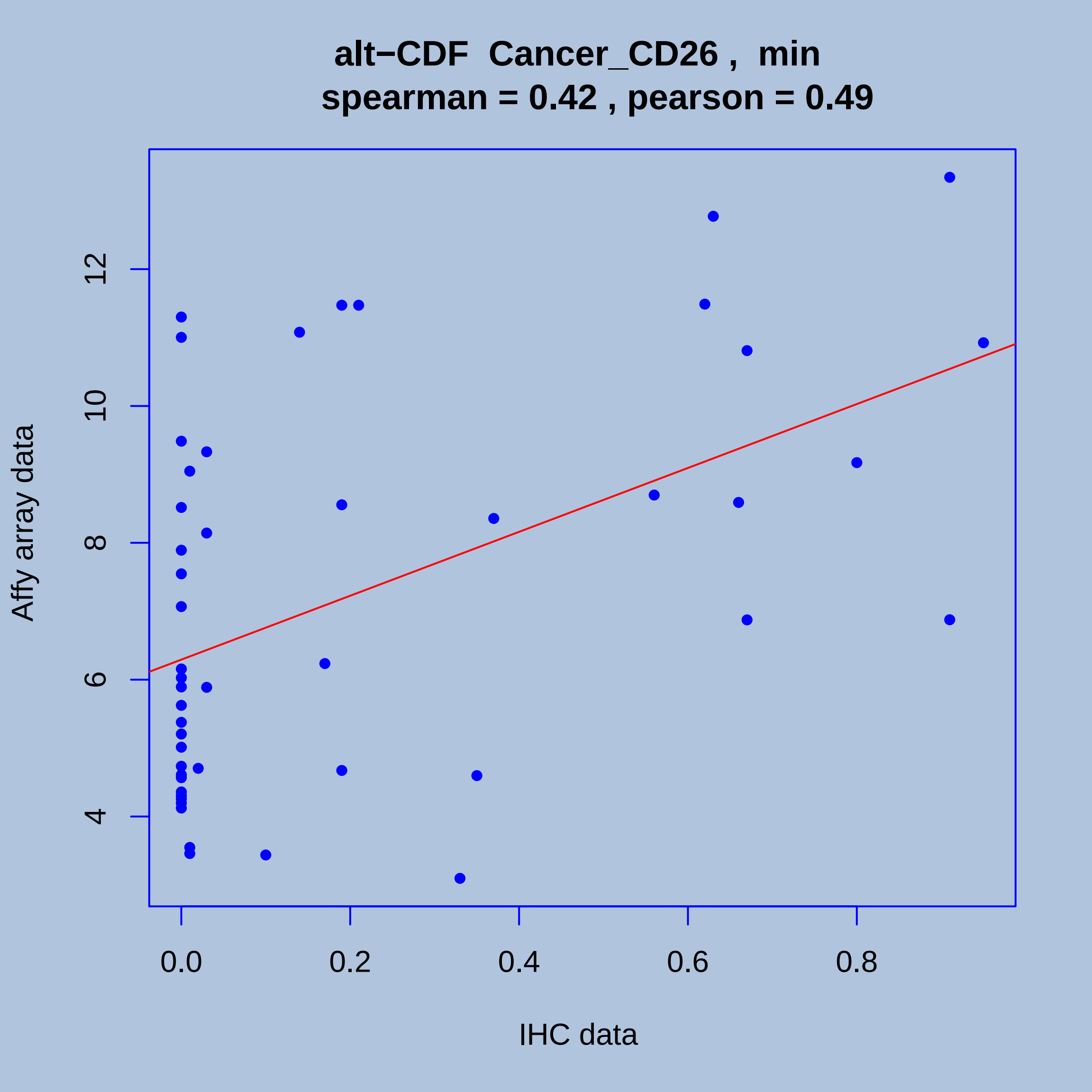
alt−CDF Cancer_CD26 , min
spearman = 0.42 , pearson = 0.49
Affy array data
IHC data
4
6
8
10
12
0.0
0.2
0.4
0.6
0.8

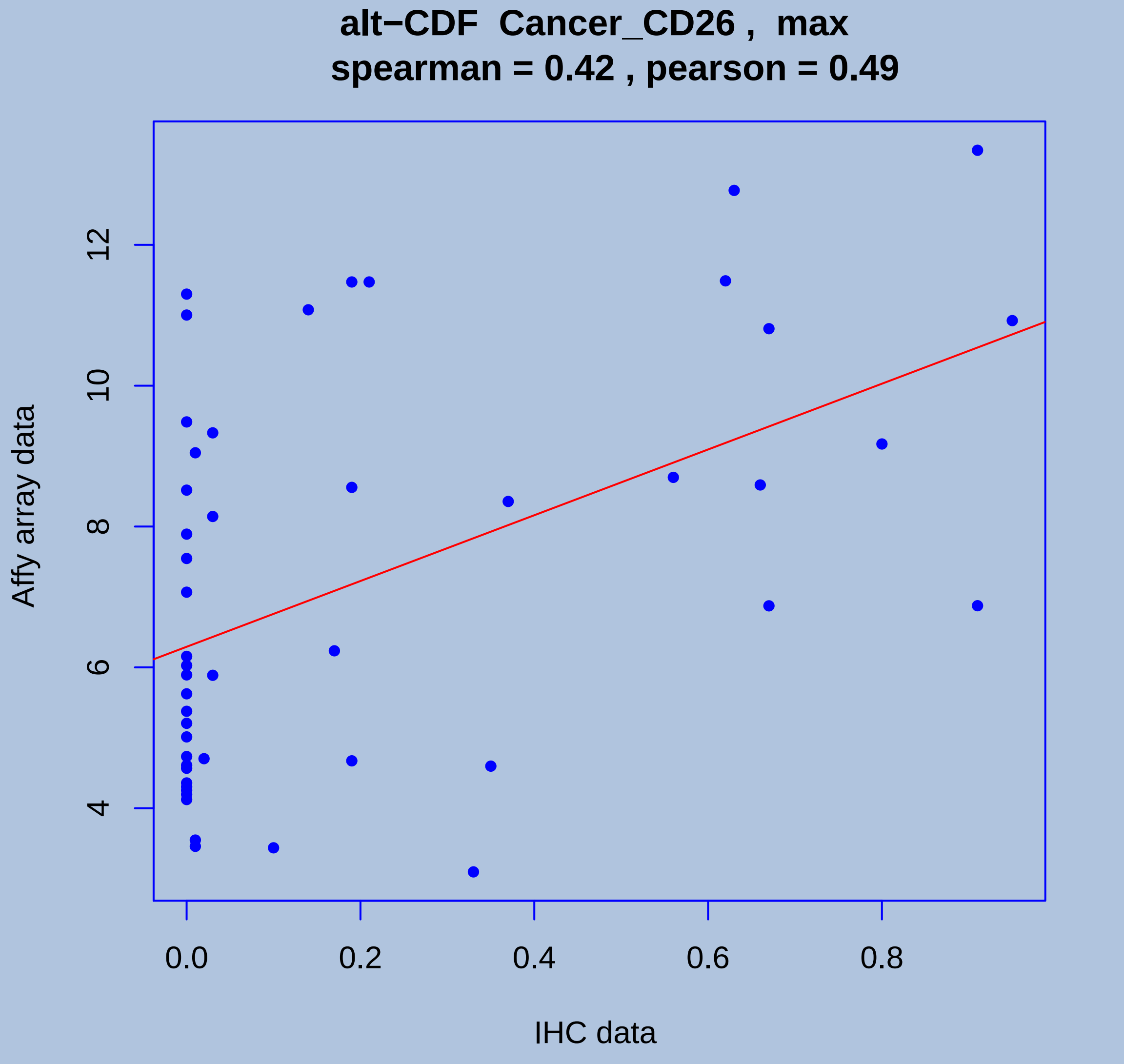

alt−CDF Cancer_CD26 , max
spearman = 0.42 , pearson = 0.49
Affy array data
IHC data
4
6
8
10
12
0.0
0.2
0.4
0.6
0.8

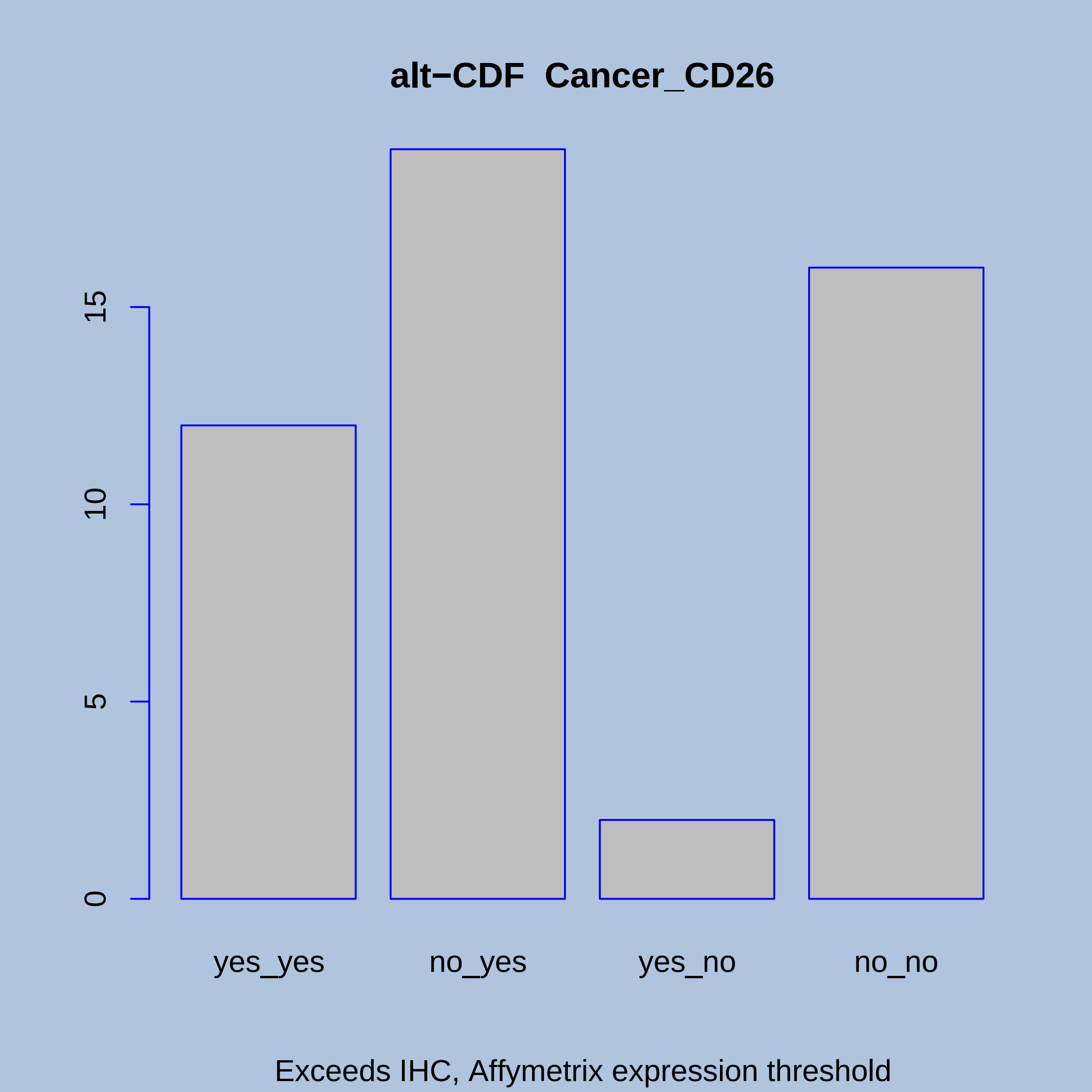
alt−CDF Cancer_CD26
0
5
10
15
yes_yes
no_yes
yes_no
no_no
Exceeds IHC, Affymetrix expression threshold
