# Supplementary material for: Correlation of mRNA and protein levels: Cell type-specific gene expression of cluster designation antigens in the prostate
Source: BMC Genomics. 2008 May 23;9:246. doi: 10.1186/1471-2164-9-246 (PMC2413246; doi:10.1186/1471-2164-9-246)
Supplement: Additional file 3 — Scatter plot of IHC staining intensity and gene expression levels determined by Affymetrix and Agilent array for LCM cells. Statistical Pearson correlation ranged from 0.26 – 0.57 and Spearman Coefficients ranged from 0.25 – 0.43. There was no positive correlation of immunolocalization data with array data for LCM stromal cells assayed by either Agilent or Affymetrix. Pearson correlation for LCM luminal cells was 0.57 and Spearman coefficient was 0.43, which were the same values as that for MACS-sorted luminal cells using updated probe set definitions. Pearson correlation between LCM stromal cell data and MACS-sorted stromal cell data assayed by Affymetrix array was 0.68, Spearman coefficient was 0.78. Pearson correlation between LCM stromal cell data analyzed by Agilent array and Affymetrix MACS-sorted stromal cell data was 0.65, Spearman coefficient was 0.55. Pearson correlation between LCM stromal cell data analyzed by Agilent array and Affymetrix LCM stromal cell data was 0.33, Spearman coefficient was 0.59. [file 1471-2164-9-246-S3.pdf]

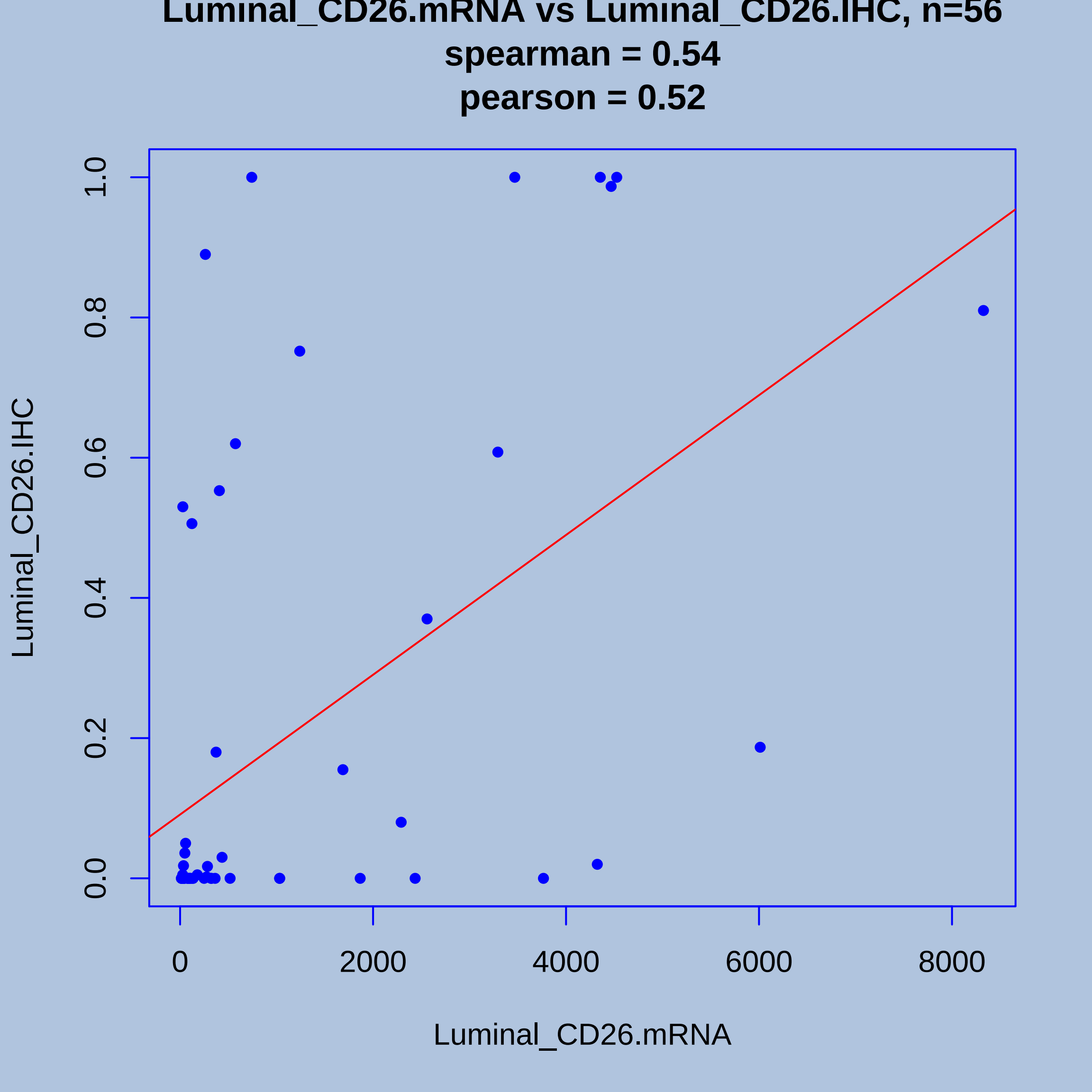

Luminal_CD26.mRNA vs Luminal_CD26.IHC, n=56
spearman = 0.54
pearson = 0.52
Luminal_CD26.IHC
0.0
0.2
0.4
0.6
0.8
1.0
0
2000
4000
6000
8000
Luminal_CD26.mRNA

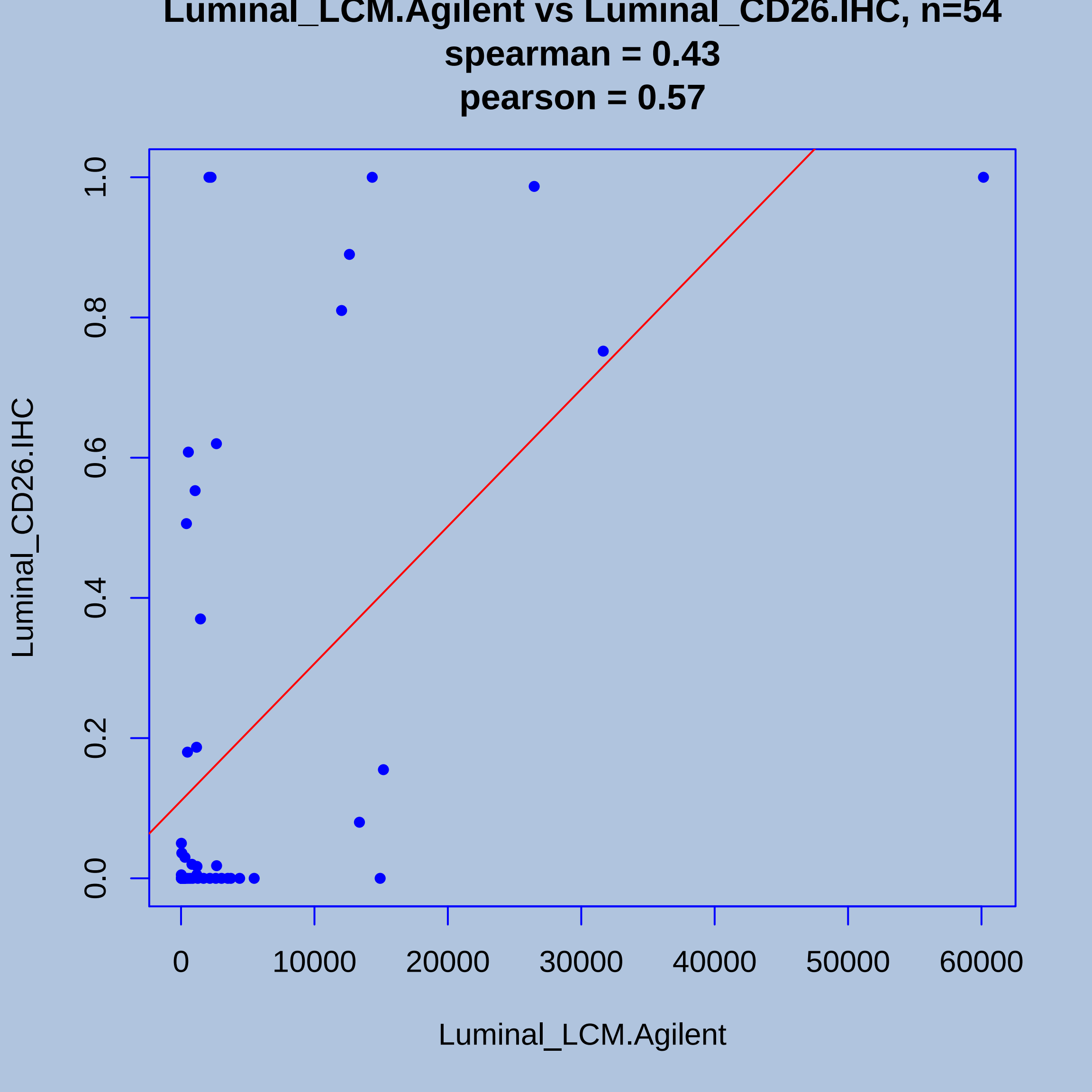
Luminal_LCM.Agilent vs Luminal_CD26.IHC, n=54
spearman = 0.43
pearson = 0.57
Luminal_CD26.IHC
0.0
0.2
0.4
0.6
0.8
1.0
0
10000
20000
30000
40000
50000
60000
Luminal_LCM.Agilent

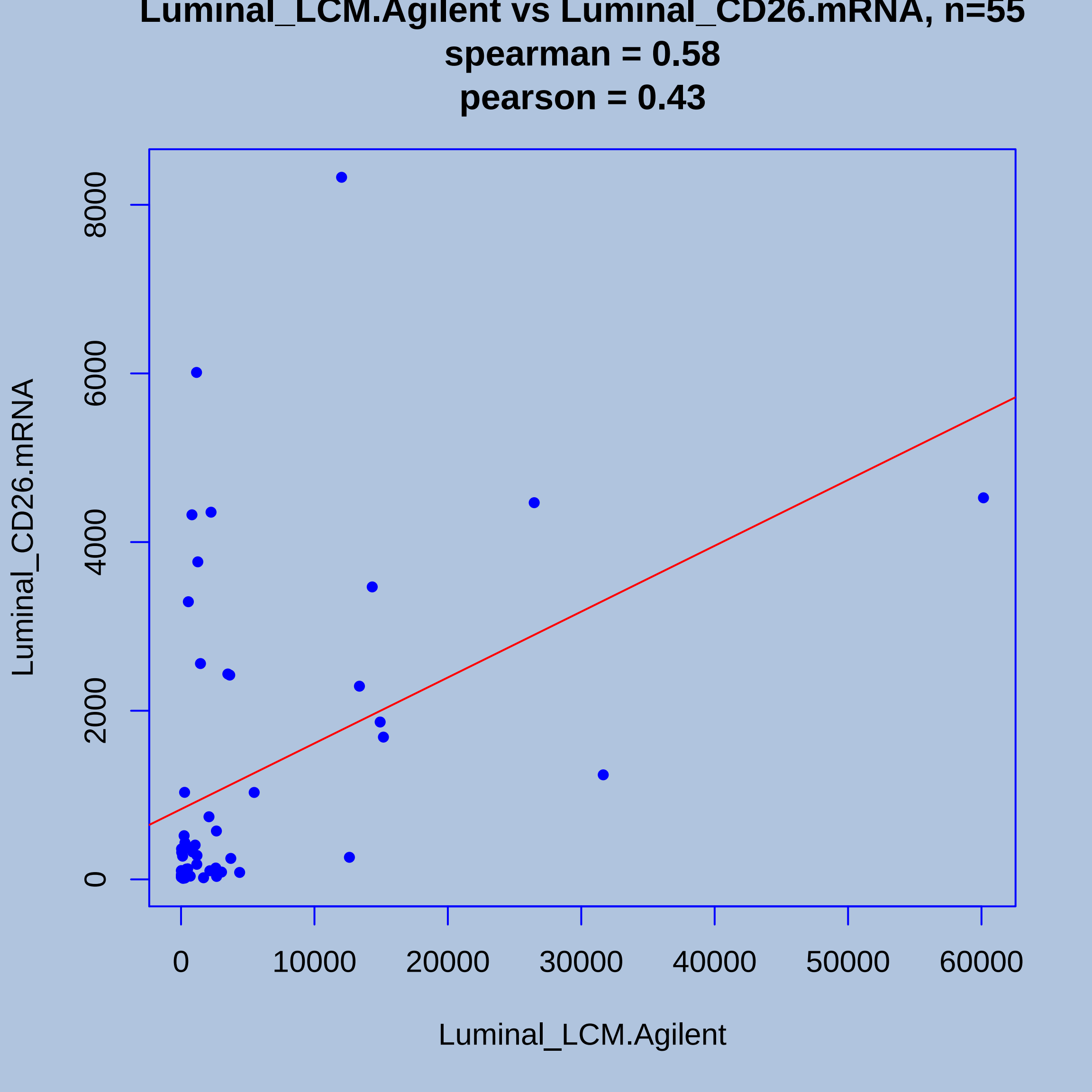
Luminal_LCM.Agilent vs Luminal_CD26.mRNA, n=55
spearman = 0.58
pearson = 0.43
Luminal_CD26.mRNA
0
2000
4000
6000
8000
0
10000
20000
30000
40000
50000
60000
Luminal_LCM.Agilent

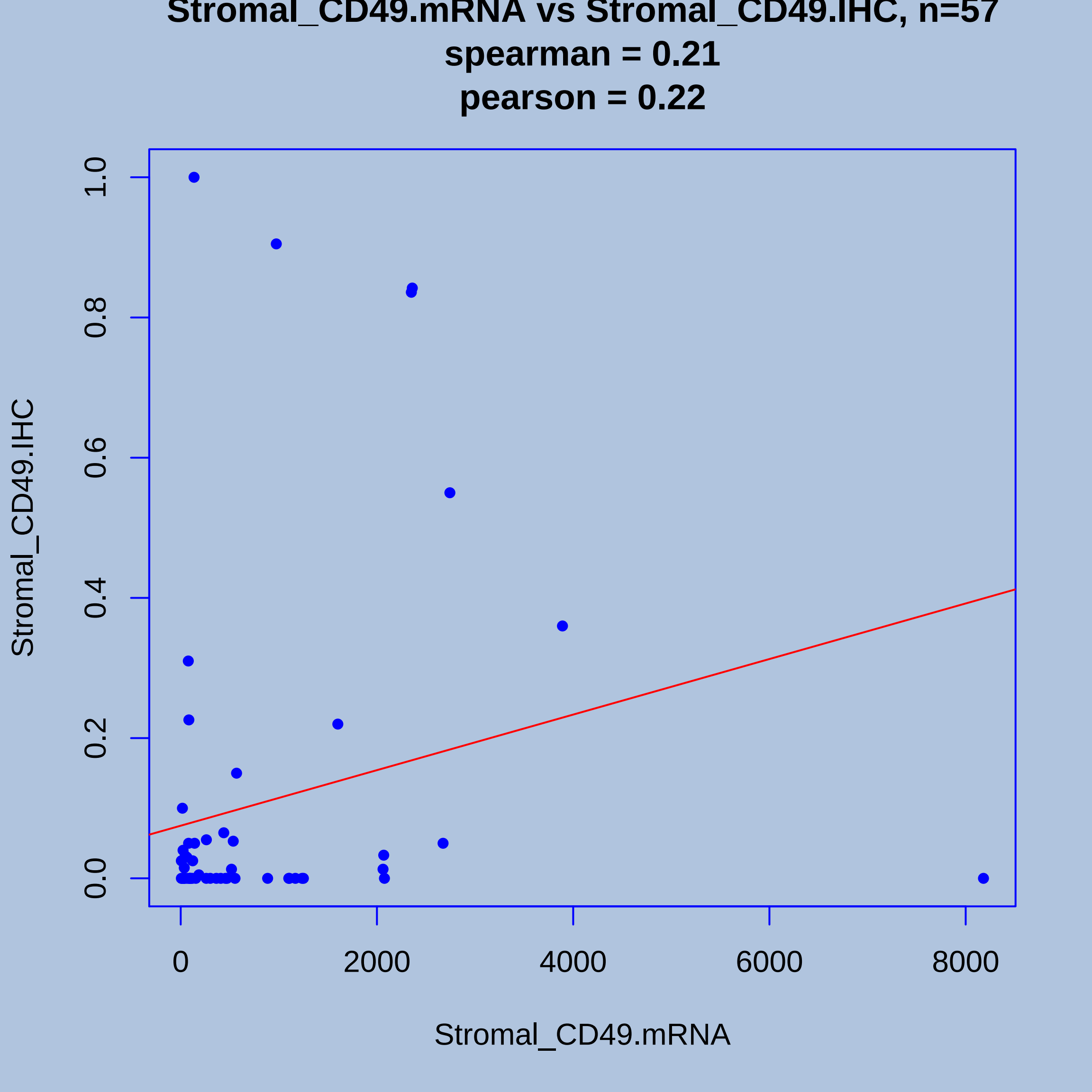
Stromal_CD49.mRNA vs Stromal_CD49.IHC, n=57
spearman = 0.21
pearson = 0.22
Stromal_CD49.IHC
0.0
0.2
0.4
0.6
0.8
1.0
0
2000
4000
6000
8000
Stromal_CD49.mRNA

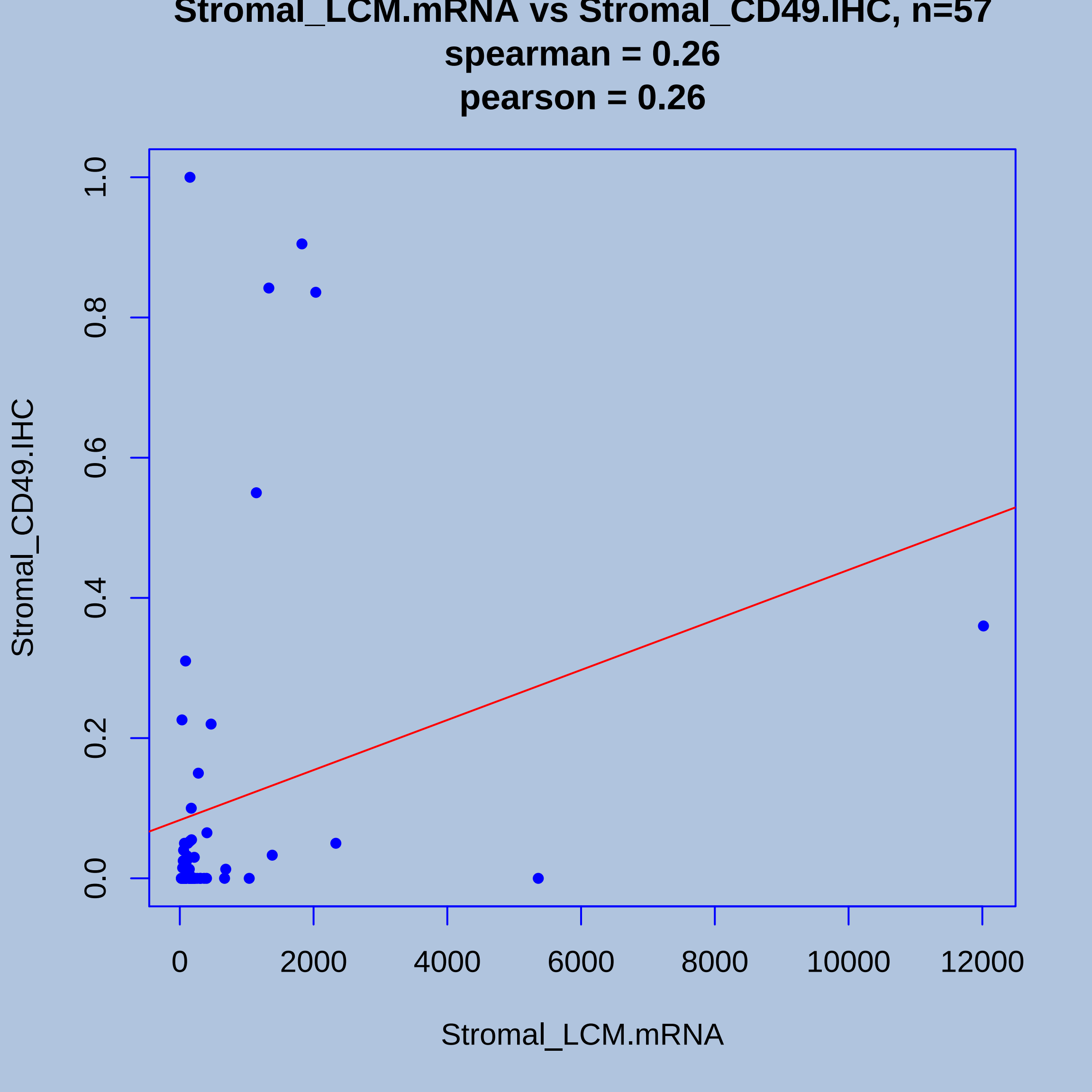
Stromal_LCM.mRNA vs Stromal_CD49.IHC, n=57
spearman = 0.26
pearson = 0.26
Stromal_CD49.IHC
Stromal_LCM.mRNA
0.0
0.2
0.4
0.6
0.8
1.0
0
2000
4000
6000
8000
10000
12000

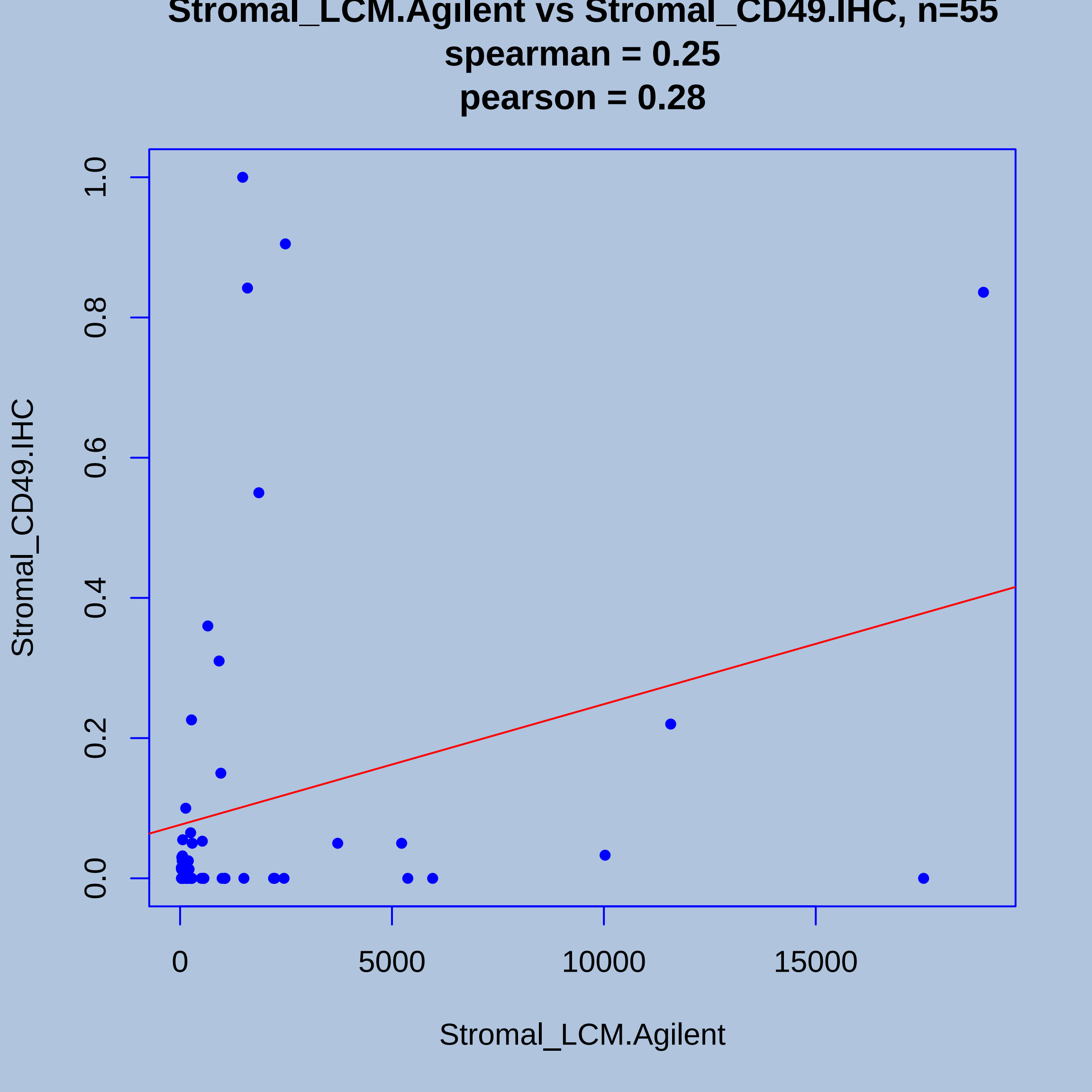
Stromal_LCM.Agilent vs Stromal_CD49.IHC, n=55
spearman = 0.25
pearson = 0.28
Stromal_CD49.IHC
0.0
0.2
0.4
0.6
0.8
1.0
0
5000
10000
15000
Stromal_LCM.Agilent

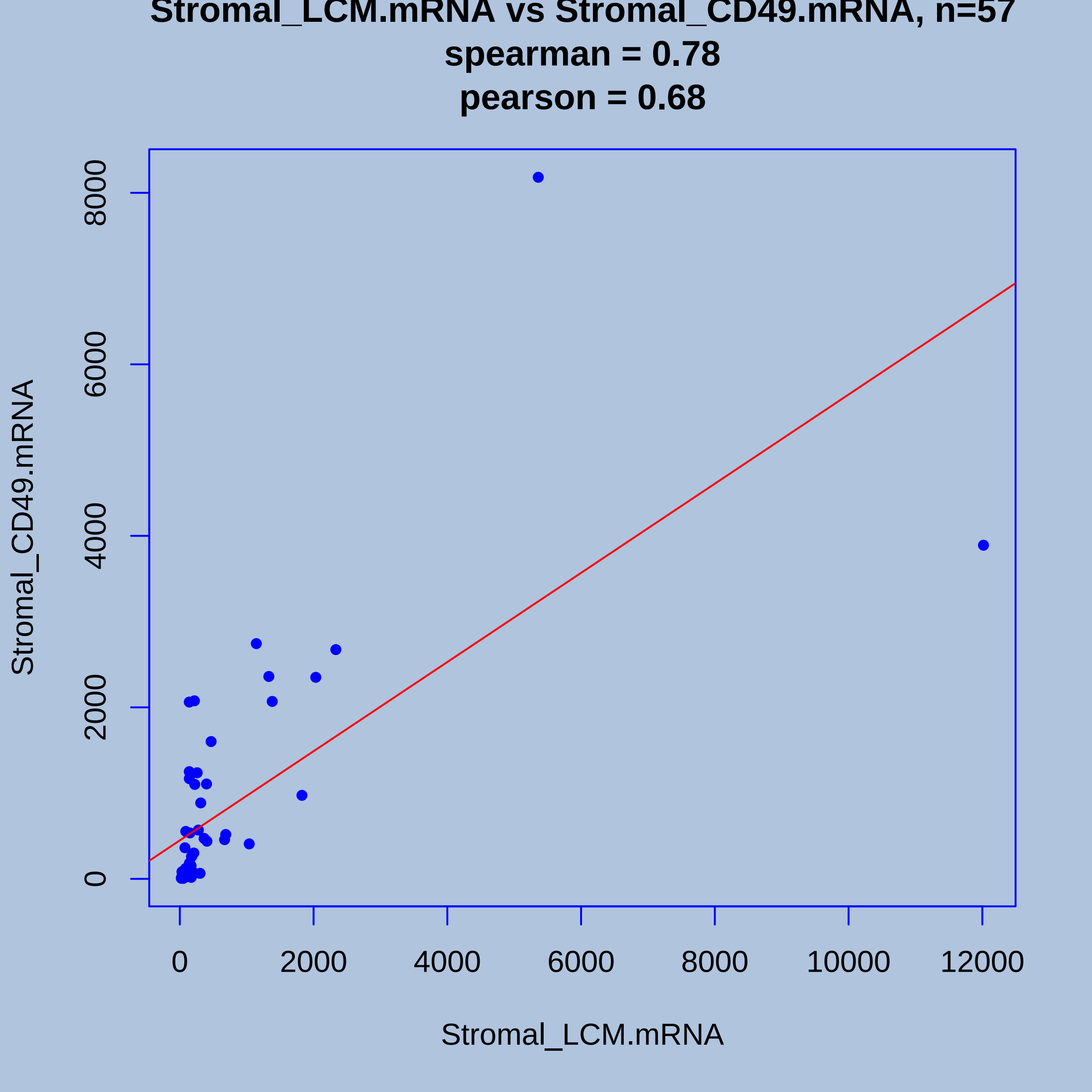

Stromal_LCM.mRNA vs Stromal_CD49.mRNA, n=57
spearman = 0.78
pearson = 0.68
Stromal_CD49.mRNA
Stromal_LCM.mRNA
0
2000
4000
6000
8000
0
2000
4000
6000
8000
10000
12000

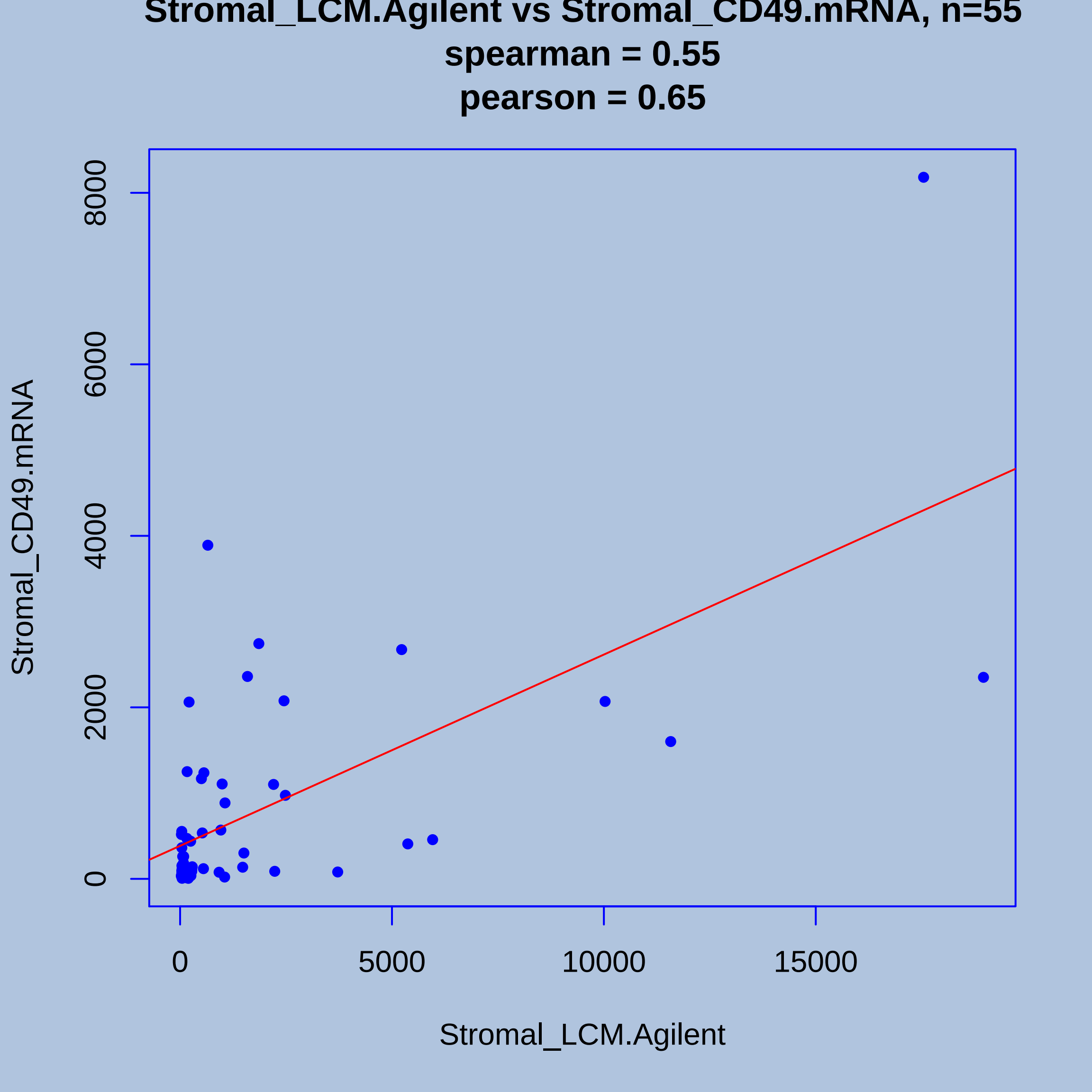
Stromal_LCM.Agilent vs Stromal_CD49.mRNA, n=55
spearman = 0.55
pearson = 0.65
Stromal_CD49.mRNA
0
2000
4000
6000
8000
0
5000
10000
15000
Stromal_LCM.Agilent

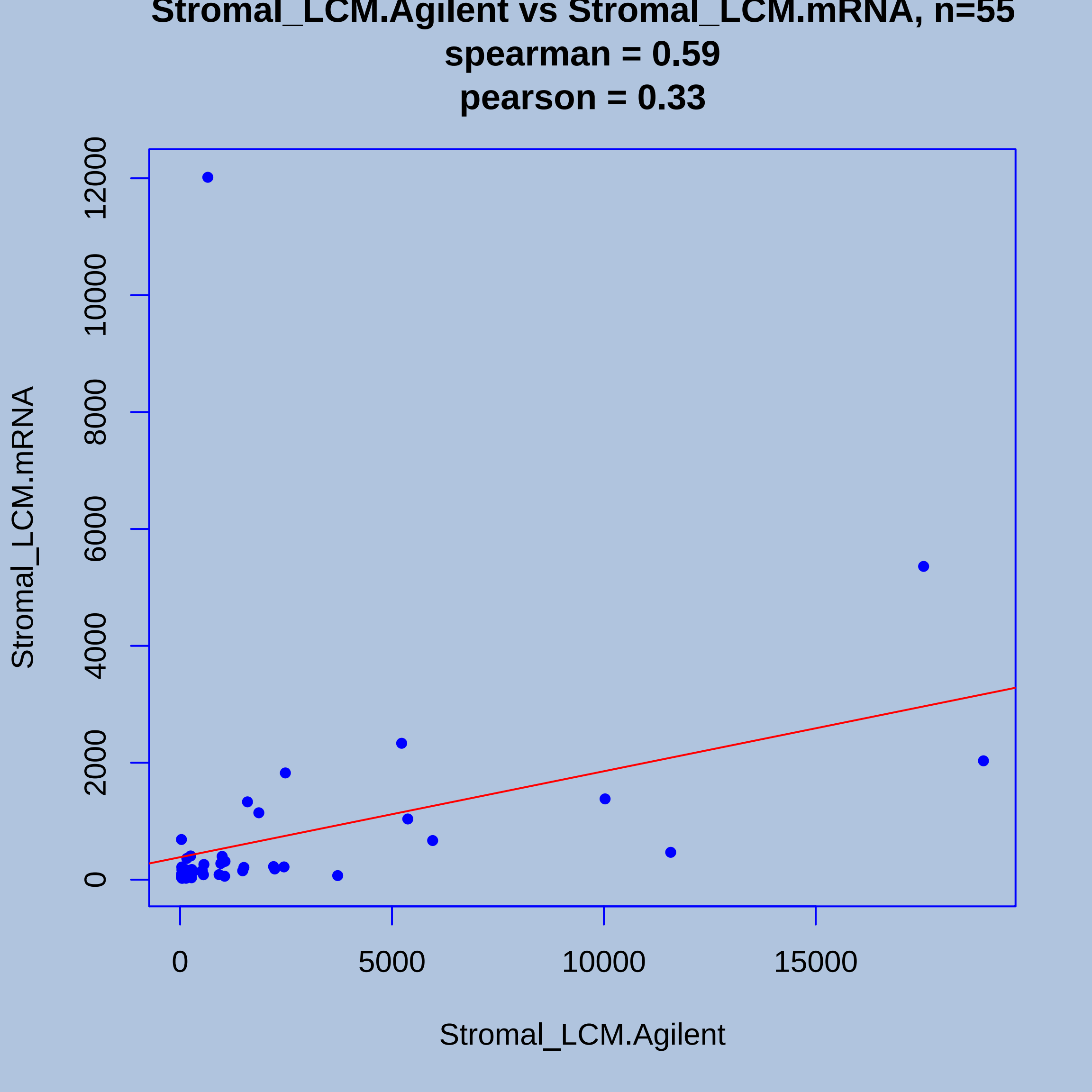

Stromal_LCM.Agilent vs Stromal_LCM.mRNA, n=55
spearman = 0.59
pearson = 0.33
Stromal_LCM.mRNA
0
2000
4000
6000
8000
10000
12000
0
5000
10000
15000
Stromal_LCM.Agilent
